# Design, Synthesis, and Evaluation of Novel Adenine-Based Hydrazones as Potential Anticancer Agents: Cytotoxicity Against A549 and MRC-5 Cell Lines with In Silico Insights

**DOI:** 10.3390/ph19101652

**Published:** 2026-10-19

**Authors:** Emre Menteşe, Nedime Çalışkan, Mustafa Emirik, Adem Güner

**Affiliations:** 1 Department of Chemistry, Faculty of Art and Sciences, Recep Tayyip Erdogan University, 53100 Rize, Türkiye; 2 Department of Medical Biology, Faculty of Medicine, Ağrı İbrahim Çeçen University, 04100 Ağrı, Türkiye

**Keywords:** adenine, hydrazone, isatin, A549 (lung cancer), molecular docking

## Abstract

**Background/Objectives:** Lung cancer remains a leading cause of global cancer-related mortality, necessitating the development of new therapeutic candidates. In this study, we aimed to design, synthesize, and biologically evaluate novel adenine-based hydrazone derivatives as potential anticancer candidates. **Methods:** A series of novel adenine–hydrazones were synthesized via the condensation of 2-(6-amino-9H-purin-9-yl)acetohydrazide with various aldehydes and isatin derivatives. Their antiproliferative activities against human lung adenocarcinoma (A549) and normal lung fibroblast (MRC-5) cell lines were evaluated using the MTT assay. Mechanistic investigations—including cell membrane damage (LDH release), oxidative stress (TOS, iROS), mitochondrial membrane potential (MMP, ΔΨm), cell cycle distribution, and apoptosis (Annexin V/PI)—were conducted for the lead derivatives, complemented by multi-target in silico molecular docking against PLK1, TrxR1, and MDM2. **Results:** Compounds **3d**, **3c**, and **4j** showed the strongest antiproliferative activity within the synthesized series against A549 cells, with IC_50_ values of 37.2 ± 1.8 µM, 41.8 ± 1.9 µM, and 42.9 ± 1.7 µM, respectively, together with comparatively more favorable A549/MRC-5 activity profiles than several other derivatives. These compounds induced oxidative stress-associated cellular injury, accompanied by mitochondrial depolarization, G2/M phase accumulation, and Annexin V-confirmed apoptotic cell death. Complementary multi-target in silico molecular docking provided theoretical insights into potential binding interactions with PLK1, TrxR1, and MDM2. **Conclusions:** Adenine–hydrazone hybrids, particularly 5-halogenated isatin derivatives such as compound **3d**, provide preliminary lead scaffolds with moderate in vitro cytotoxicity against lung cancer cells, serving as a platform for future structural optimization to enhance potency and selectivity.

## 1. Introduction

According to 2024 data from the World Health Organization (WHO), there were an estimated 20.6 million new cancer cases and 9.8 million associated deaths worldwide. These figures indicate that one in five people develops cancer during their lifetime, while one in nine men and one in 13 women lose their lives to the disease. Lung cancer is the most frequently diagnosed cancer type, with approximately 2.6 million new cases (12.8%), followed by breast (11.8%), colorectal (9.9%), prostate (7.5%), and stomach (4.7%) cancers [1,2,3]. Despite notable advances in screening and therapeutic modalities, the escalating incidence and mortality rates of cancer continue to pose a formidable challenge to global healthcare systems.

Among various malignancies, lung cancer is particularly devastating, accounting for approximately 12.8% of all newly diagnosed cases (about 2.6 million cases) and for 18–19% of all cancer-related deaths worldwide. A critical clinical hurdle is that only a small fraction (approximately 17–18%) of these cases are detected at an early localized stage, whereas the vast majority are diagnosed only after regional or distant metastasis has occurred [4,5,6].

Pathologically, cancer is a debilitating disease characterized by aberrant, uncontrolled cellular proliferation [7]. A multitude of factors contribute to this global health threat, including genetic predisposition which plays a pivotal role in malignancies such as breast cancer and a diverse array of environmental and lifestyle risk factors [8,9]. These include tobacco consumption, alcohol abuse, exposure to ionizing radiation, excessive X-ray exposure, poor dietary habits, widespread environmental pollution, and an aging global population, collectively cementing cancer as a critical threat to human longevity worldwide.

Epidemiological data indicate distinct distribution patterns of cancer types across genders; breast, colorectal, lung, uterine, and thyroid cancers represent the most prevalent malignancies in women, while lung, colorectal, stomach, prostate, and liver cancers are most frequently diagnosed in men [10,11,12]. This relentless rise in cancer-related mortality has intensified global research efforts aimed at identifying novel and selective therapeutic agents [13].

From a biochemical perspective, adenine is an endogenous purine derivative synthesized primarily in the liver in humans, but it is also dietary, occurring in brewer’s yeast and various vegetables. It serves as a fundamental building block for critical biomolecules essential to cell physiology, such as adenosine triphosphate (ATP) in cellular bioenergetics and deoxyribonucleic acid (DNA) in genomic replication and protein synthesis. Furthermore, adenine constitutes an integral part of metabolic coenzymes such as nicotinamide adenine dinucleotide (NAD) and flavin adenine dinucleotide (FAD). Beyond these physiological roles, adenine exhibits compelling pharmacological properties; recent investigations have shown that it can suppress IgE-mediated mast cell activation [10] and attenuate lipopolysaccharide-induced inflammatory reactions [13]. Additionally, it exhibits neuroprotective effects, increasing the survival of rat cerebellar Purkinje cells [14,15], and has been shown to improve erythrocyte storage stability in whole blood [16]. Crucially for oncology, adenine is increasingly recognized for its intrinsic anticancer potential across multiple tumor cell lines, where it triggers cell cycle arrest and apoptotic cell death [17,18,19].

Purine scaffolds are ubiquitously distributed among both natural products and synthetic molecules [20,21,22]. This structural versatility has positioned purine-based compounds as highly attractive targets in drug discovery. For instance, purine derivatives have been developed as potent interferon inducers for treating chronic hepatitis C [23,24,25], as well as cytostatic antitumor agents that disrupt cellular division through robust microtubule-degrading activities [26]. Furthermore, these scaffolds act as heat shock protein 90 (Hsp90) inhibitors, promoting the proteasomal degradation of oncogenic client proteins [27]. In particular, adenine derivatives have been identified as inhibitors of the cysteine protease cathepsin K for osteoporosis therapy [28,29], and as highly selective phosphodiesterase inhibitors for gastrointestinal and neurological pathologies [30]. Consequently, purine and adenine congeners continue to be extensively explored for their broad-spectrum anticancer, antiviral, and antimicrobial activities [31,32]. Specifically, their capacity to impair DNA replication and disrupt cell cycle checkpoints highlights their potential as versatile scaffolds for targeted oncology drug design.

**Figure 1:**
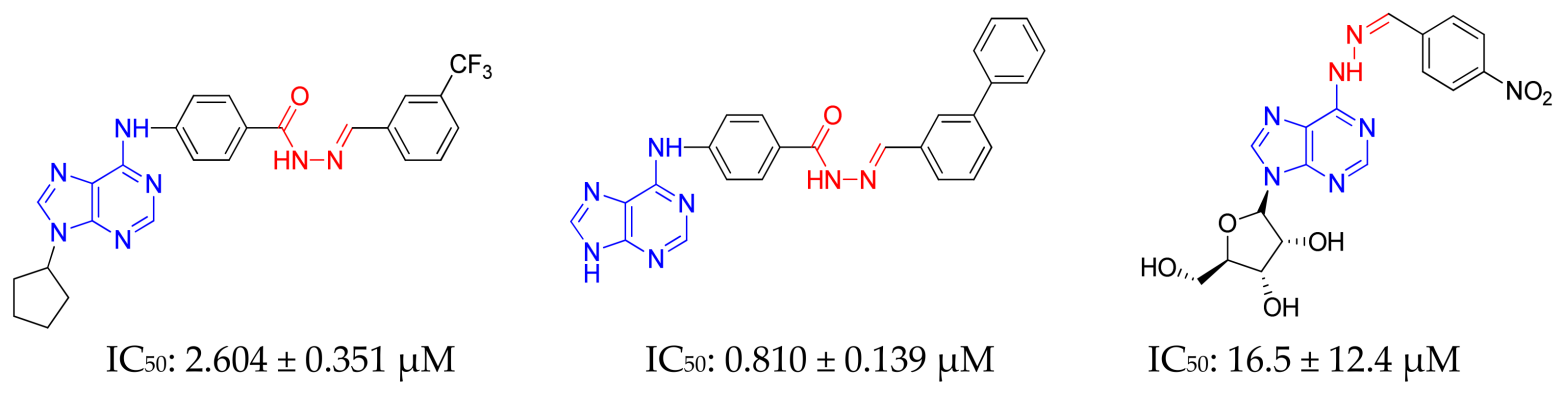
Some highly effective purine base derivatives against lung cancer.

To date, several purine-based drugs have shown high efficacy against lung cancer, as highlighted in Figure 1 [33,34]. Unlike known hydrazone derivatives, purine hydrazones form hybrid systems with enhanced hydrazone binding potential and extended conjugation by combining a purine pharmacophore with a hydrazine. The adenine unit additionally provides several hydrogen-bond donor and acceptor sites together with π-stacking capability, structural features that are characteristic of nucleotide-recognizing binding pockets. No cellular uptake or transport experiments were performed in the present study, and no specific uptake or transport mechanism is implied. Hydrazones possess various biological activities, although most of them do not have the exclusive activities of purines [35,36,37,38].

Purine bases—particularly adenine and heterocyclic derivatives containing 1,2,3-triazole rings—hold great importance in medicinal chemistry; furthermore, studies have shown them to possess promising activity against various types of cancer, including colon carcinoma, breast cancer, and prostate cancer [39,40,41,42], Furthermore, these structures are found in many drugs targeting various diseases. For example, mercaptopurine, thioguanine, and cladribine are used in the treatment of various types of leukemia. Triazole rings feature prominently in antifungal drugs such as fluconazole and ravuconazole [43,44,45].

Heterocyclic scaffolds, particularly benzimidazole-, triazole-, and coumarin-based hybrid systems, constitute a privileged class of compounds in medicinal chemistry owing to their broad and tunable spectrum of biological activities, including acetylcholinesterase, urease, and lipase inhibition as well as antibacterial and anticancer properties [46,47,48,49,50,51,52,53,54,55,56]. Our research group has been actively engaged in the rational design, synthesis, and biological evaluation of such heterocyclic hybrids, with particular emphasis on benzimidazole-, benzoxazolone-, and triazole–coumarin-based derivatives as candidate anti-Alzheimer’s, antimicrobial, and enzyme-inhibitory agents [46,47,48,49,50,51,52,53]. More recently, these efforts have been extended toward benzimidazole- and triazole–coumarin-based hybrids exhibiting potent activity against breast cancer, further underscoring the versatility of heterocyclic frameworks as anticancer pharmacophores [54,55,56]. Building on this accumulated experience in heterocyclic hybrid chemistry, the present study extends our group’s research interest to exploring purine (adenine)-based hydrazone hybrids as a novel class of anticancer agents against lung adenocarcinoma.

In the present design, each structural fragment was selected for a defined purpose. The adenine unit was retained as the molecular recognition element: it supplies hydrogen-bond donor and acceptor sites and π-stacking capability that are characteristic of nucleotide-binding pockets, and adenine itself has been reported to induce cell cycle arrest and apoptosis in tumor cells [17,18,19]. The acetohydrazide bridge was introduced as a conformationally flexible linker that carries an additional amide NH/C=O pair, extends conjugation across the molecule and offers further hydrogen-bonding opportunities. The terminal fragment was then varied systematically. Isatin (1H-indole-2,3-dione) derivatives were selected because this scaffold is an established antiproliferative pharmacophore associated with cell cycle perturbation and apoptosis induction, and because its 5-position tolerates halogen, methyl, methoxy and nitro substituents, allowing the electronic contribution of this position to be probed directly (compounds **3a–g**). Aromatic and heteroaromatic aldehydes were used to explore a wider steric and electronic space at the same position of the scaffold (compound **4a–p**). The expected added value of this combination was therefore to merge a nucleobase recognition motif with two complementary classes of hydrazone terminus, and to establish which electronic and steric character at this terminus is associated with antiproliferative activity in A549 cells.

## 2. Results and Discussion

### 2.1. Chemistry

**Scheme 1:**
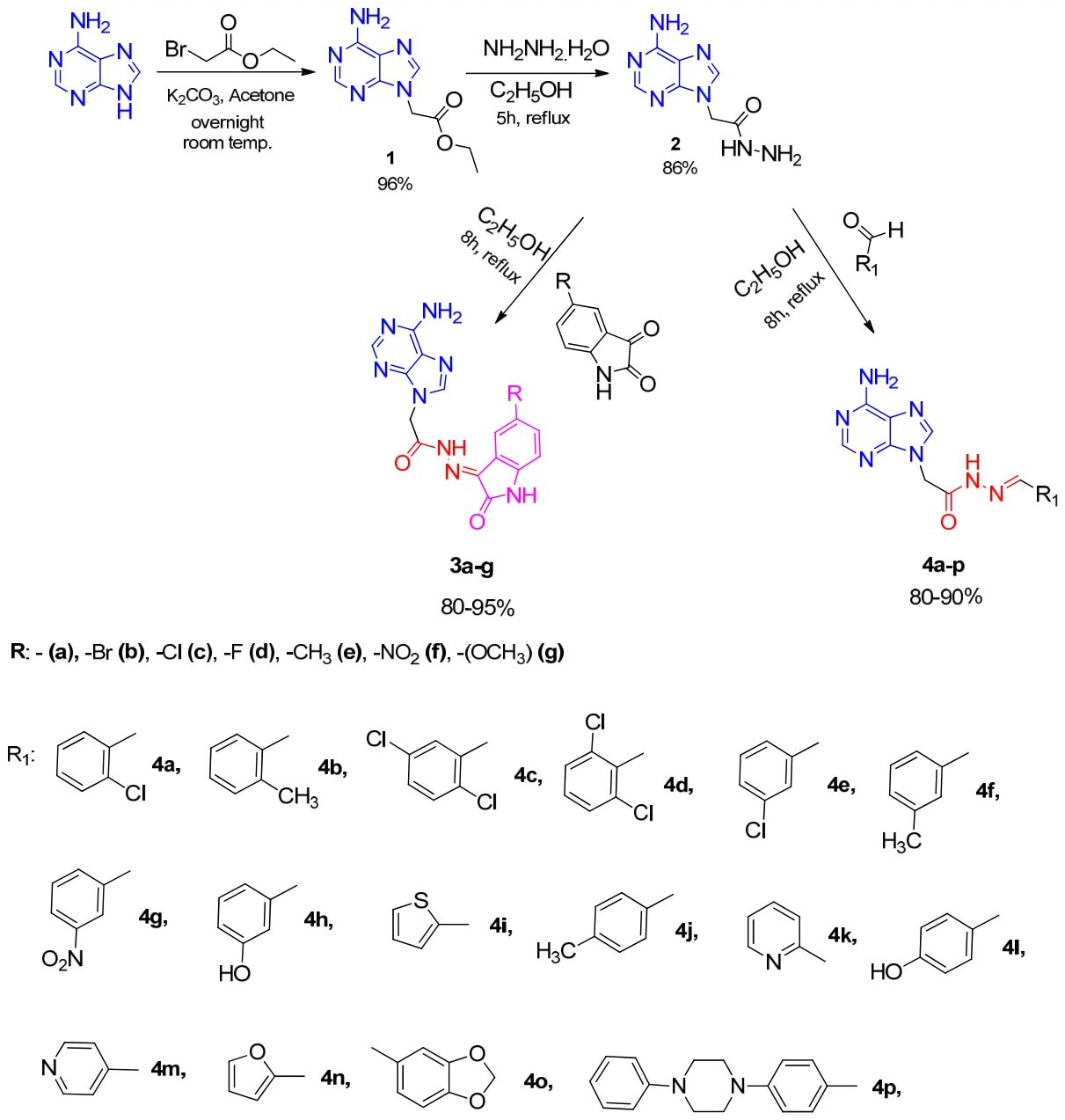
Synthetic route for the preparation of target adenine–hydrazone derivatives.

First, the intermediate ethyl 2-(6-amino-9H-purin-9-yl)acetate (**1**) was synthesized by the alkylation of adenine with ethyl bromoacetate in the presence of potassium carbonate in acetone, following literature procedures [34]. This ester was subsequently reacted with hydrazine monohydrate under reflux to yield 2-(6-amino-9H-purin-9-yl)acetohydrazide (**2**) in 86% yield [34]. Finally, the condensation of hydrazide **2** with various substituted isatins and aldehydes in refluxing ethanol with catalytic acetic acid successfully delivered the target Schiff bases (**3a–g** and **4a–p**) in good to excellent yields (82–93%) (Scheme 1).

All synthesized compounds were analyzed for purity by thin-layer chromatography (TLC) before structural and biological evaluation. The spectral data (IR, ^1^H NMR, and ^13^C NMR) of these novel compounds (**3a–g** and **4a–p**) were consistent with the proposed chemical structures. In the IR spectra, characteristic absorption bands were observed at approximately 3330 and 3140 cm^−1^ corresponding to NH-NH_2_ stretching, 1690 cm^−1^ for the carbonyl (C=O) groups, and 1590 cm^−1^ for the imine (C=N) bonds. In the ^1^H NMR spectra, the primary amine (NH_2_) protons of the adenine core appeared as a singlet near 7.20 ppm, while the hydrazide NH and imine CH (N=CH) signals resonated at approximately 11.80 and 8.20 ppm, respectively. Due to *cis/trans* amide conformer isomerism (typically in an 80:20 ratio), the aliphatic NCH_2_ methylene protons resolved as two distinct singlets at approximately 5.40 and 5.00 ppm. In the ^13^C NMR spectra, the amide C=O carbon was identified at 168 ppm, and the C=N imine carbon resonated between 156 and 160 ppm. The adenine C_2_ and C_6_ carbons were observed at approximately 142 and 152 ppm, respectively, whereas the aliphatic NCH_2_ carbon resonated at approximately 44 ppm.

In the ^1^H-NMR spectra of compound **4a–p**, several proton signals were observed as two distinct sets at different chemical shifts. This duplication arises from two distinct phenomena inherent to the arylidene-hydrazide scaffold: *E*/*Z* geometrical isomerism about the C=N double bond, and *cis/trans* conformational isomerism about the CO–NH amide bond, arising from restricted rotation around this partially double-bond-character linkage. These two effects originate from different structural features—configurational (C=N) versus conformational (amide C–N)—and should not be conflated, although both contribute to the observed signal splitting.

**Scheme 2:**
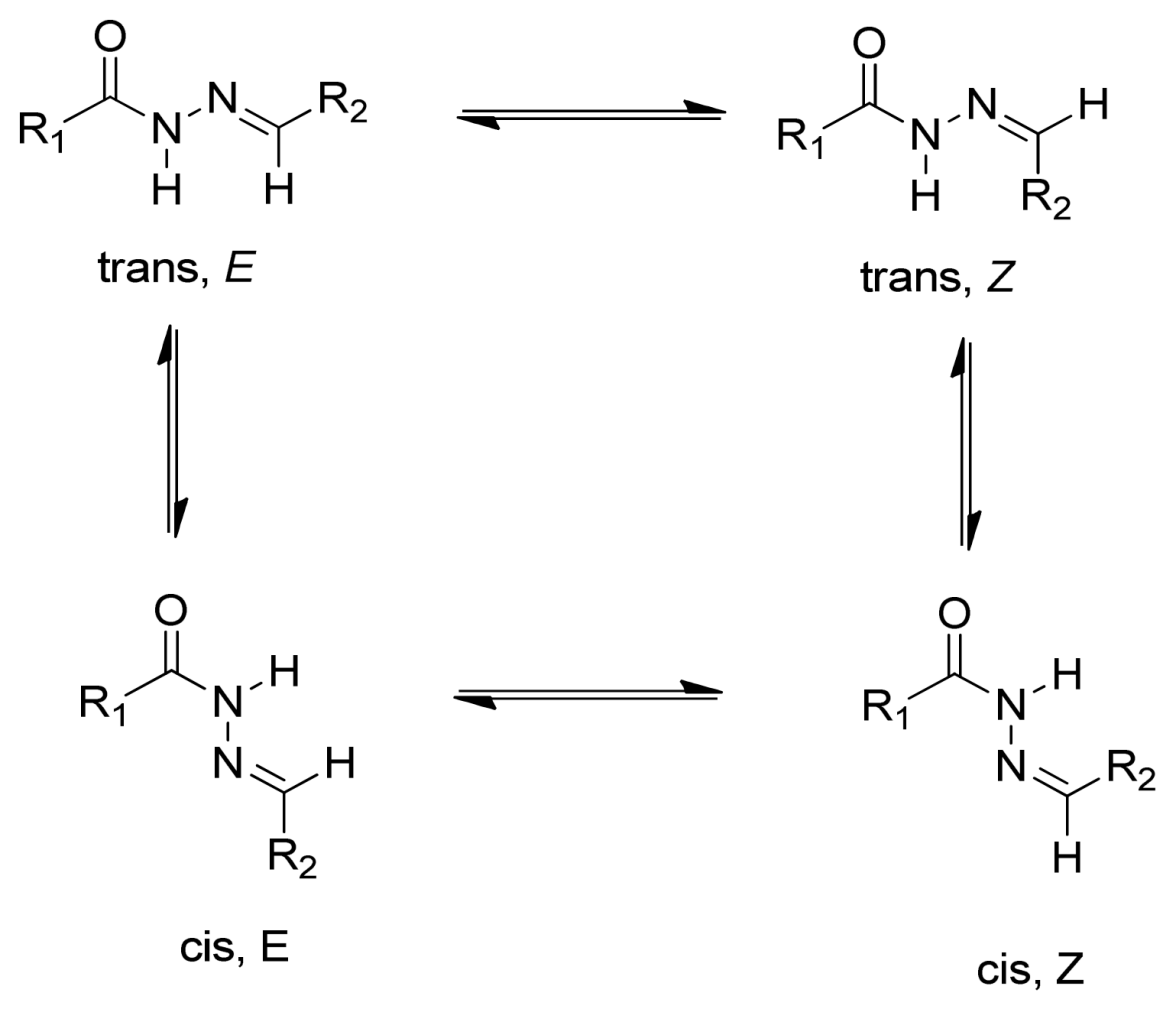
*E*/*Z* geometrical isomers and *cis/trans* amide conformer of compound **4a–p**.

According to the literature, hydrazone derivatives bearing a C=N bond preferentially adopt the *E* configuration in polar solvents such as DMSO-d_6_, whereas the *Z* isomer may become more populated in less polar media [57,58,59,60,61,62,63,64]. Independently of this configurational preference, slow rotation about the CO–NH amide bond gives rise to *cis/trans* amide rotamers, which accounts for the duplicated N–CH_2_ and N–H signals observed in the spectra (Scheme 2).

The relative population of each species was determined by integration of the corresponding, well-resolved ^1^H-NMR signals. In practice, the ratios were obtained from the integrals of the two baseline-resolved N–CH_2_ singlets (≈5.4 and 5.0 ppm) and, where sufficiently separated, from the duplicated N=CH singlets, using identical integration limits and automatic baseline correction. The values are reported to the nearest 5% and carry an estimated uncertainty of about ±2%, which accounts for the small differences between the ratios derived from different signals of the same sample. The *E*/*Z* geometrical isomers and *cis/trans* amide conformers of compound **4a-p** are illustrated in Scheme 2. The predominance of the *E*-configured, amide–rotamer mixture in DMSO-d_6_ is consistent with previously reported hydrazone derivatives in polar solvents [57,58,59,60,61,62,63,64]. Accordingly, the duplication of the NCH_2_ and NH signals is attributed to the combined, independent contributions of C=N geometrical isomerism and restricted amide bond rotation, rather than a single isomerization process.

**Figure 2:**
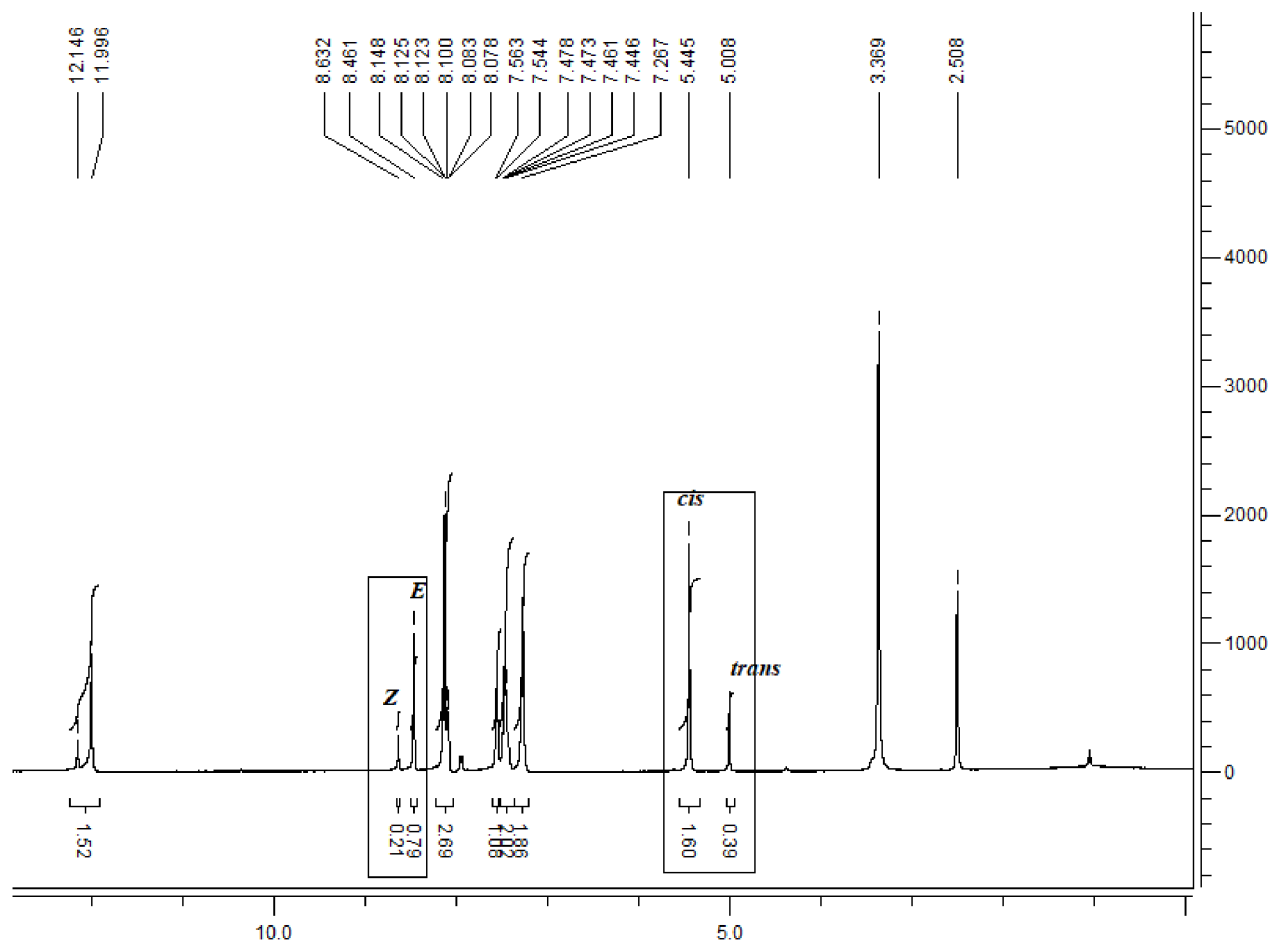
^1^H-NMR spectra of compound **4a**.

The observed ratios (75:25–80:20) are consistent with values reported for related *N*-acylhydrazones and reflect the partial double-bond character of the CO–NH amide bond, which arises from conjugation of the nitrogen lone pair with the carbonyl π-system. This conjugation raises the rotational barrier about the amide C–N bond to a range (typically ≈ 16–19 kcal/mol for related aroylhydrazides [57,58,59,60,61,62,63,64]) at which interconversion of the *cis* and *trans* rotamers is slow on the NMR timescale at room temperature, so that the two species are observed as separate, sharp signals rather than as a single time-averaged signal (Figure 2). Because this barrier originates mainly from the amide linkage itself, which is common to all members of the series, the rotamer ratio was found to lie within a narrow range (75:25–80:20) irrespective of whether the arylidene substituent was electron-donating (e.g., **4b**, **4f**, **4g**) or electron-withdrawing (e.g., **4a**, **4c**, **4e**), indicating that the remote substituent modulates the equilibrium only marginally; steric bulk close to the amide nitrogen and the polarity of the solvent are expected to be the dominant factors, consistent with the single, well-defined ratio observed for each compound in DMSO–d_6_. Variable-temperature NMR, which would allow the rotational barrier to be quantified directly from the coalescence temperature, was beyond the scope of the present study.

The condensation was chemoselective for the terminal hydrazide nitrogen and did not involve the exocyclic C6-NH_2_ group of adenine. In the ^1^H-NMR spectra of all products, the adenine NH_2_ singlet is retained at 7.19–7.31 ppm (2H), whereas the NH_2_ resonance of the hydrazide group of 2 is no longer observed and is replaced by the N=CH singlet (7.95–8.63 ppm) together with the amide NH signal (11.16–13.17 ppm); in the ^13^C-NMR spectra the adenine C6 resonance remains essentially unchanged (≈156 ppm), as expected for an unmodified exocyclic amino group. This selectivity is consistent with the greater nucleophilicity of the hydrazide terminus relative to the exocyclic amino group of adenine, whose lone pair is delocalized over the electron-deficient purine ring.

### 2.2. Cell Viability (Antiproliferative Activity)

**Figure 3:**
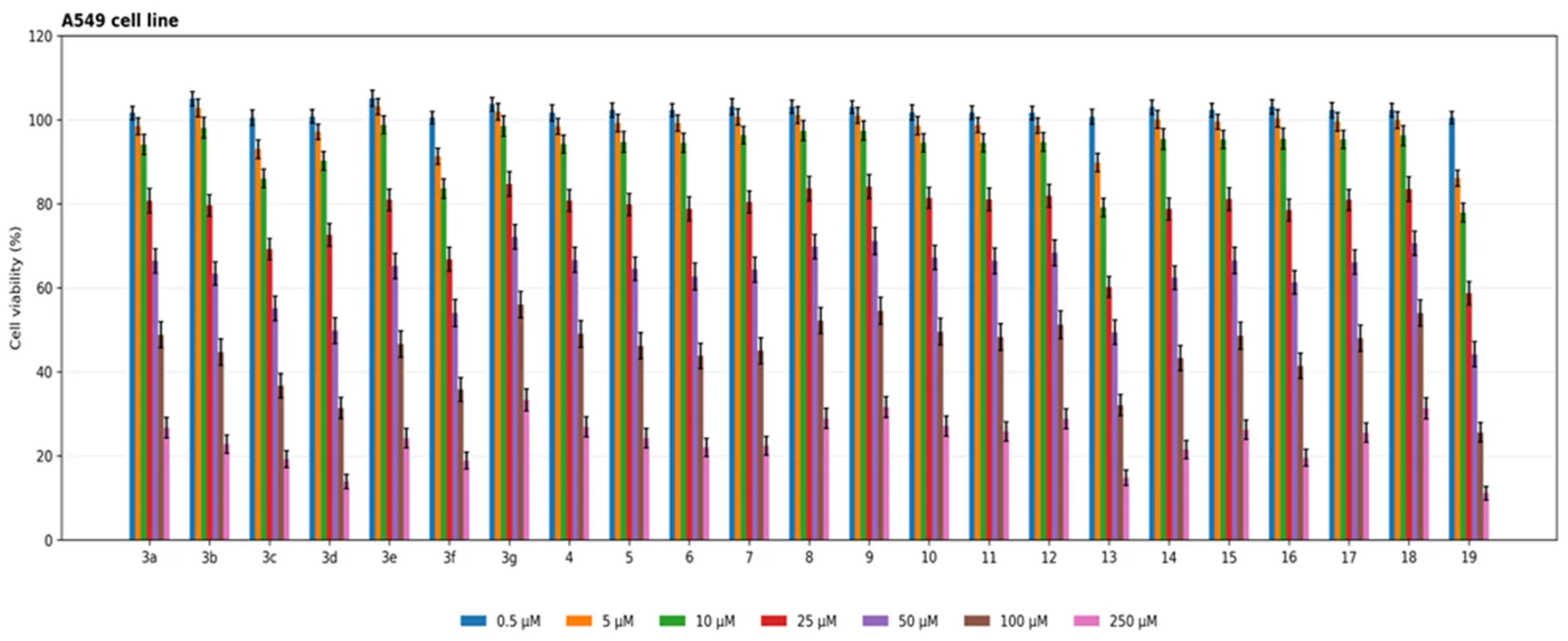

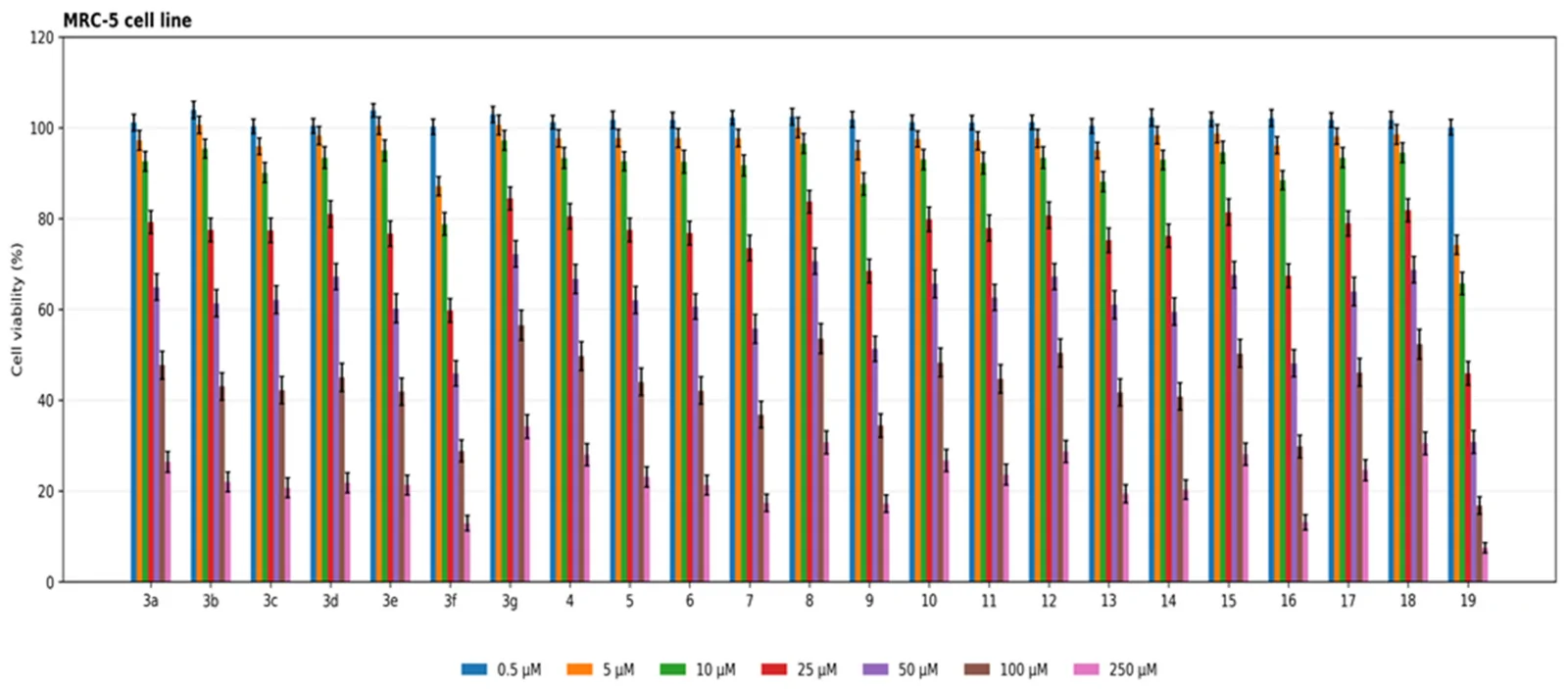
Cell viability (%) of A549 human lung adenocarcinoma and MRC-5 normal lung fibroblast cell lines after 48 h treatment with novel synthesized adenine–hydrazone compounds at various concentrations (0.5–250 µM).

The antiproliferative activities of the synthesized adenine–hydrazone derivatives (**3a–g** and **4a–p**) were evaluated against human lung adenocarcinoma (A549) and normal human lung fibroblast (MRC-5) cell lines using the MTT assay after 48 h of exposure. Doxorubicin, a clinically established chemotherapeutic agent, was included as a positive control. The concentration-dependent cell viability curves are presented in Figure 3, and the calculated half-maximal inhibitory concentration (IC_50_) values are summarized in Table 1.

Among the newly synthesized derivatives, compounds **3c**, **3d**, and **4j** showed the strongest antiproliferative activity within the series and displayed comparatively more favorable A549/MRC-5 activity profiles than several other derivatives (Figure 5, Table 1). Compound **3d** (the 5-fluoro isatin derivative) exhibited the strongest antiproliferative activity within the series against A549 cells, with an IC_50_ value of 37.2 ± 1.8 µM, while displaying a significantly higher IC_50_ of 73.3 ± 2.2 µM in MRC-5 cells, yielding a selectivity index (SI) of 1.97. Compound **3c** (the 5-chloro analogue) maintained a closely comparable profile, with an IC_50_ of 41.8 ± 1.9 µM in A549 cells and 80.4 ± 2.8 µM in MRC-5 cells (SI = 1.92). In the aldehyde-derived series, compound **4j** (bearing a para-methyl substituent) represented the most active analogue, with an IC_50_ value of 42.9 ± 1.7 µM in A549 cells and 63.8 ± 2.1 µM in MRC-5 cells (SI = 1.49).

Doxorubicin was included as an established cytotoxic positive control to confirm assay responsiveness rather than as a structurally or mechanistically matched comparator for the adenine–hydrazone derivatives. Doxorubicin exhibited IC_50_ values of 1.84 ± 0.15 µM in A549 cells and 5.24 ± 0.42 µM in MRC-5 cells. Accordingly, direct structure–activity or mechanistic comparisons between doxorubicin and the synthesized adenine–hydrazone derivatives were not made. The relative activities of compounds **3c**, **3d**, and **4j** were interpreted within the synthesized series. It should also be noted that the selectivity indices calculated for **3c**, **3d** and **4j** (SI = 1.49–1.97) remain below the SI > 3 value that is generally regarded as indicative of selective antitumor behavior, and were lower than that obtained for doxorubicin under the present experimental conditions (SI = 2.85). These derivatives are therefore described as compounds showing comparatively more favorable A549/MRC-5 activity profiles within the synthesized series, and not as selective anticancer agents.

Since the present biological evaluation was limited to the A549 lung adenocarcinoma model, the observed antiproliferative and mechanistic findings should be interpreted within this cellular context, and confirmation across additional lung cancer subtypes will be required to establish broader applicability.

**Figure 4:**
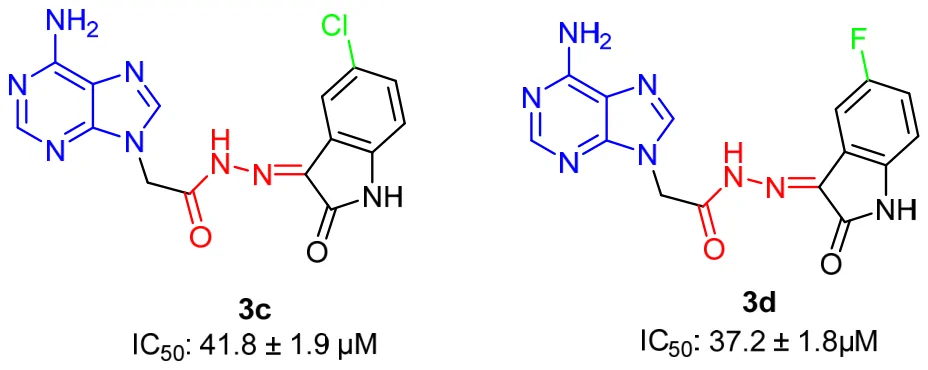
Synthetic compounds with high activity at the 5-position (**3c**, **3d**).

**Figure 5:**
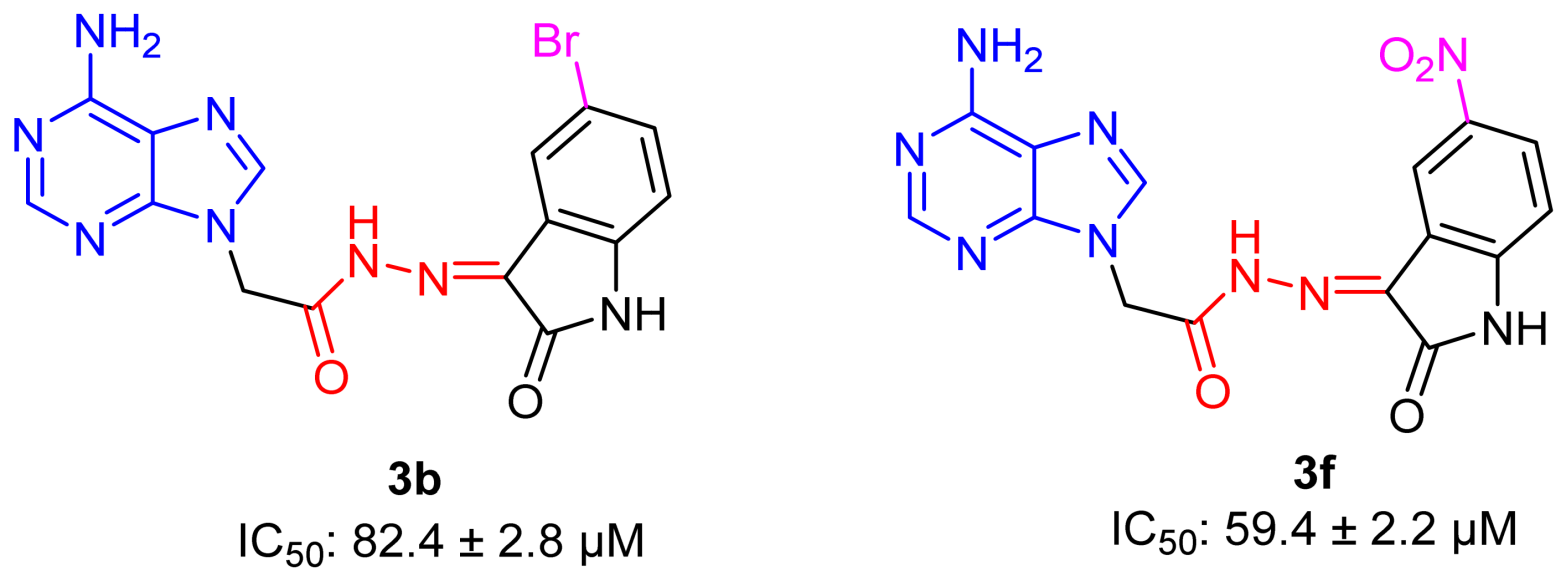
Biologically active but less selective compounds.

A clear structure–activity relationship (SAR) was observed based on the substitution patterns of the derivatives. Within the isatin-containing subgroup (**3a–g**), substitution at the 5-position of the oxindole ring strongly influenced the biological profile. The 5-halogenated derivatives demonstrated superior profiles; the 5-fluoro derivative **3d** showed the most favorable A549/MRC-5 activity profile within this subgroup, followed closely by the 5-chloro analogue **3c** (Figure 4). The 5-bromo derivative **3b** showed intermediate activity (IC_50_ = 82.4 ± 2.8 µM against A549), indicating that smaller halogens are preferred at this position. In contrast, the 5-nitro derivative **3f**, despite retaining measurable antiproliferative activity (IC_50_ = 59.4 ± 2.2 µM in A549), displayed higher toxicity toward normal MRC-5 cells (IC_50_ = 47.6 ± 2.1 µM, SI = 0.80), indicating that strong electron-withdrawing nitro groups compromise therapeutic selectivity (Figure 5). Furthermore, the introduction of electron-donating groups at the 5-position, such as methyl (**3e**) or methoxy (**3g**), dramatically reduced antiproliferative potency (IC_50_ of 88.5 ± 2.9 µM and 126.8 ± 3.8 µM in A549 cells, respectively). The unsubstituted isatin derivative **3a** showed only moderate activity (IC_50_ = 95.7 ± 3.1 µM in A549), further confirming the critical role of appropriate halogen substitution in optimizing the biological response.

Among the aldehyde-derived hydrazones (**4a–p**), most compounds showed weak to moderate cytotoxic effects, with the notable exception of the para-methyl derivative **4j**. Bulkier polar substituents or patterns reactive to normal cells, such as the 2,6-dichloro derivative **4d** and the *N*-phenylpiperazine derivative **4p**, resulted in poor therapeutic windows due to either diminished activity in A549 cells or severe toxicity toward normal MRC-5 cells (e.g., compound **4p** showed an IC_50_ of 39.4 ± 1.8 µM against MRC-5 cells). Other derivatives, including **4f**, **4i**, **4l**, and **4o**, remained relatively weak, indicating that not all structural modifications within the aldehyde series are beneficial. These findings indicate that the cytotoxic behavior of these adenine-based derivatives is highly sensitive to the electronic and steric nature of the aromatic substituents.

### 2.3. Selection of Compounds for Mechanistic Studies

Compounds selected for further biochemical and mechanistic investigations were prioritized not only on the basis of their antiproliferative activity in A549 cells, but also according to their ability to represent chemically meaningful trends within the series. Since the primary aim of the follow-up experiments was to determine whether the observed loss of viability was associated with cell membrane damage, oxidative imbalance, mitochondrial dysfunction, and regulated cell death-related events, the selected compounds were chosen to provide both biological relevance and structure–activity interpretability.

Accordingly, compound **3d** was selected as the principal lead candidate of the series, as it displayed one of the most favorable profiles, combining comparatively strong antiproliferative activity within the series with better preservation of MRC-5 viability. This compound also represented the most favorable structural direction identified within the isatin-containing subgroup, indicating that 5-fluoro substitution was associated with the most favorable A549/MRC-5 activity profile within this subset. Compound **3c** was included as a closely related analogue to enable direct comparison within the same 5-substituted 1H-indole-2,3-dione series (**3a–g**) and to determine whether the favorable behavior of **3d** extended to another halogenated counterpart with a distinct electronic character. The inclusion of both compounds therefore anchored the mechanistic analysis to the most favorable substitution trend identified within the isatin framework.

In addition, compound **4j** was selected as a representative of a distinct substitution class outside the isatin subgroup. Although its structural context differed from that of the halogenated isatin derivatives, it showed favorable activity within the series together with a comparatively favorable A549/MRC-5 profile. Its inclusion therefore allowed us to determine whether the mechanistic effects observed for the halogenated isatin derivatives would also be retained in a structurally different but biologically active analogue. Collectively, this selection strategy enabled a focused and chemically interpretable investigation centered on compounds **3c**, **3d**, and **4j**, with **3c** and **3d** representing a favorable halogenated isatin trend and **4j** serving as a structurally distinct comparator outside that subgroup.

### 2.4. Membrane Damage and Oxidative Stress-Associated Redox Imbalance

To determine whether the decrease in cell viability was accompanied by biochemical cellular damage, LDH release, total oxidant status (TOS), and the intracellular GSH/GSSG ratio were evaluated in A549 cells following 48 h treatment with the selected compounds at their respective IC_50_ concentrations (Table 2). Basal LDH activity in untreated control cells was 6.4 ± 0.5 U/L. Treatment with all selected compounds significantly increased LDH release, indicating cell membrane damage. The strongest elevation was observed with the lead compound **3d**, which increased LDH to 15.9 ± 1.0 U/L, corresponding to a 2.48-fold increase relative to control (*p* < 0.001). Compound **4j** also markedly elevated LDH release to 13.8 ± 0.9 U/L (2.16-fold increase, *p* < 0.01), whereas compound **3c** increased LDH leakage to 12.6 ± 0.8 U/L (1.97-fold increase, *p* < 0.01). The extent of LDH release was consistent with the viability results, supporting that the reduction in cell viability was associated with biochemical evidence of cellular injury rather than only metabolic suppression.

To examine whether oxidative stress contributed to this injury, intracellular oxidant burden was assessed by TOS measurement and expressed as µM H_2_O_2_ equivalent/L. Control cells showed a basal TOS level of 14.8 ± 1.1 µM H_2_O_2_ Eq/L. Among the tested compounds, compound **3d** produced the greatest increase, elevating TOS to 27.6 ± 1.9 µM H_2_O_2_ Eq/L, corresponding to a 1.86-fold increase over control (*p* < 0.001). Compound **4j** increased TOS to 24.3 ± 1.7 µM H_2_O_2_ Eq/L (1.64-fold, *p* < 0.01), while compound **3c** increased oxidant levels to 22.0 ± 1.5 µM H_2_O_2_ Eq/L (1.49-fold, *p* < 0.01). The parallel increase in LDH leakage and TOS suggests that membrane injury was accompanied by oxidative stress-related processes.

Because glutathione is a principal intracellular antioxidant system, the GSH/GSSG ratio was measured to evaluate redox homeostasis. Control cells showed a healthy ratio of 8.7 ± 0.6. Treatment with all compounds significantly reduced this value, indicating disruption of intracellular redox balance. The strongest decrease was again observed with compound **3d**, which reduced the GSH/GSSG ratio to 4.9 ± 0.4, corresponding to a 43.7% decrease relative to control (*p* < 0.001). Compound **4j** reduced the ratio to 5.5 ± 0.4 (36.8% decrease, *p* < 0.01), whereas compound **3c** lowered it to 6.2 ± 0.4 (28.7% decrease, *p* < 0.01). Since the GSH/GSSG ratio reflects intracellular redox status rather than absolute glutathione content, these results should be interpreted as evidence of altered redox homeostasis rather than direct depletion of total glutathione.

Taken together, the coordinated increase in LDH release, elevation in oxidant burden, and reduction in the GSH/GSSG ratio demonstrate that compounds **3c**, **3d**, and **4j** induced oxidative stress-associated cellular damage in A549 cells. Among them, compound **3d** showed the most pronounced effect, while compounds **3c** and **4j** produced moderate but still highly significant disturbances in membrane integrity and redox balance.

### 2.5. Intracellular Reactive Oxygen Species (iROS) Generation

**Figure 6:**
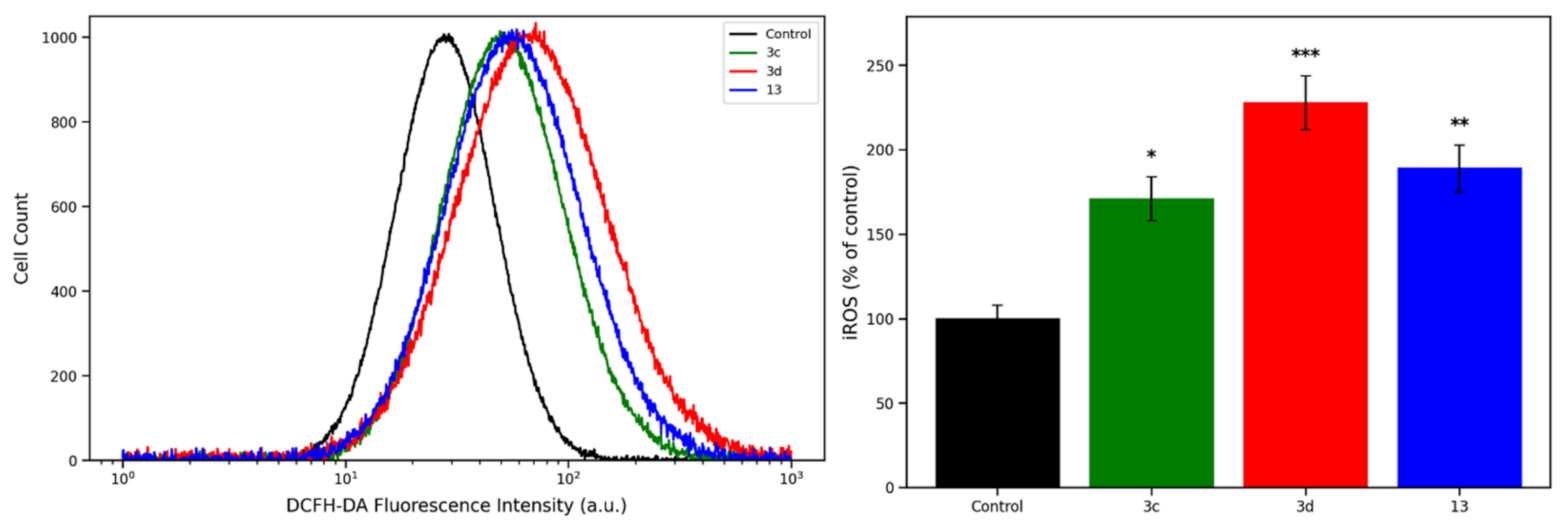
Quantification of intracellular reactive oxygen species (iROS) generated in A549 lung cancer cells following treatment with representative compounds. Cellular iROS accumulation was measured using DCFH-DA dye via flow cytometry after a 48-h incubation at respective IC50 values. Values represent the percentage relative to untreated control cells and are displayed as mean ± SD from three distinct biological replicates (n = 3). Significance was determined by one-way ANOVA with Tukey’s post hoc comparison (* *p* < 0.05, ** *p* < 0.01, *** *p* < 0.001 vs. control).

To further determine whether the oxidative imbalance observed in A549 cells was associated with increased intracellular oxidant production, reactive oxygen species (ROS) generation was evaluated by flow cytometry using DCFH-DA staining following 48 h treatment with the selected compounds at their respective IC_50_ concentrations (Figure 6). Exposure to all selected compounds significantly increased intracellular ROS levels compared with control cells. The most pronounced increase was observed in cells treated with compound **3d**, which elevated DCF fluorescence intensity to 228 ± 16% of control (*p* < 0.001). Compound **4j** also produced a marked increase in intracellular ROS generation, reaching 189 ± 14% of control (*p* < 0.01), whereas compound **3c** induced a moderate but still highly significant elevation to 171 ± 13% of control (*p* < 0.05).

Notably, the pattern of intracellular ROS production was perfectly consistent with the increase in TOS levels and the reduction in the GSH/GSSG ratio observed in the preceding biochemical assays. In particular, compound **3d**, which produced the strongest elevation in TOS and the greatest reduction in glutathione redox balance, also showed the highest intracellular ROS accumulation. Compounds **3c** and **4j** followed the same general trend, although with slightly lower magnitudes. This close concordance supports the view that the redox imbalance induced by these derivatives is accompanied by enhanced intracellular oxidant generation rather than reflecting only extracellular or secondary metabolic changes. Collectively, these findings indicate that intracellular ROS overproduction is a prominent component of the cellular response to compounds **3c**, **3d**, and **4j**, and further support the involvement of oxidative stress-associated mitochondrial dysfunction and regulated apoptotic cell death.

### 2.6. Mitochondrial Membrane Potential (ΔΨm)

**Figure 7:**
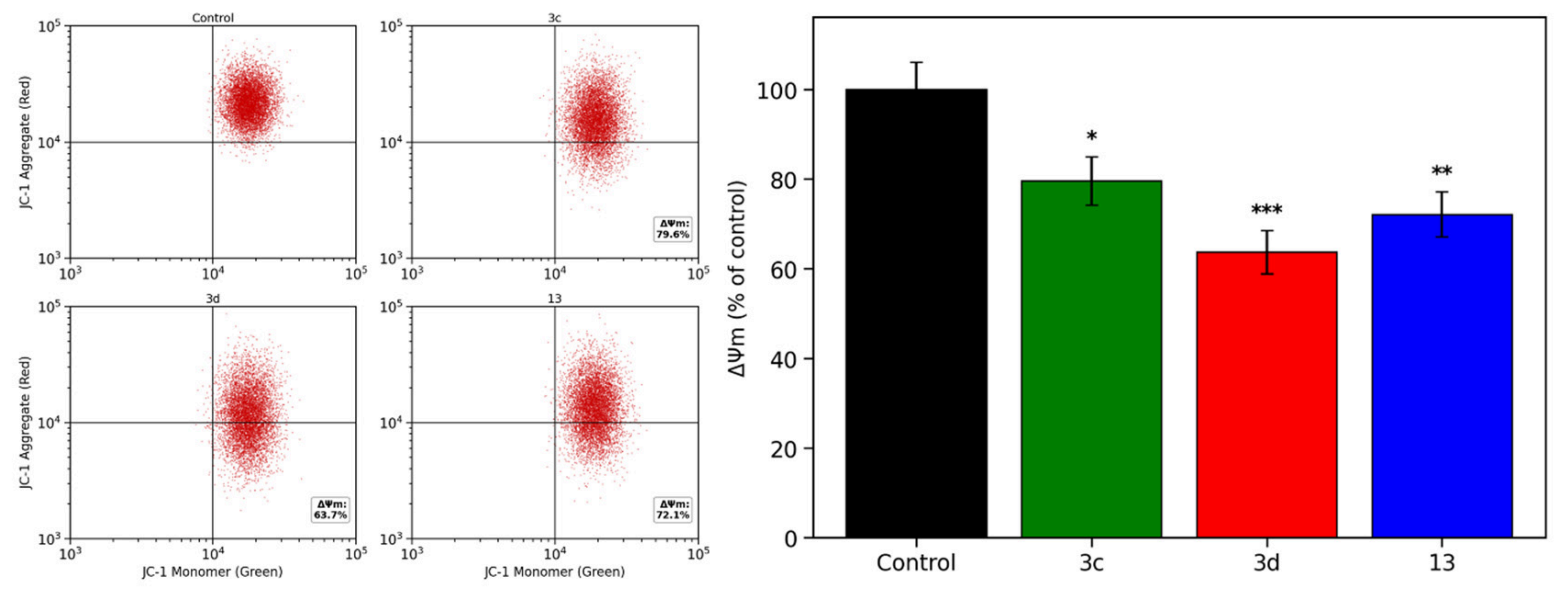
Representative flow cytometric JC-1 dot plots illustrating mitochondrial membrane potential (ΔΨm) alterations in A549 cells after 48 h treatment with compounds **3c**, **3d**, and **4j** at their respective IC_50_ doses. Exposure to the test compounds induces a marked transition from polarized (J-aggregates) to depolarized (J-monomers) mitochondrial states relative to untreated controls. Statistical significance versus the untreated control was assessed using one-way ANOVA followed by Tukey’s post hoc test. * *p* < 0.05, ** *p* < 0.01, and *** *p* < 0.001.

To determine whether oxidative stress-associated cytotoxicity was accompanied by mitochondrial dysfunction, mitochondrial membrane potential (ΔΨm) was evaluated after 48 h exposure to the selected compounds in A549 cells using JC-1 dye (Figure 7). Treatment with compound **3c** caused a moderate decrease in ΔΨm, reducing mitochondrial membrane potential to 79.6 ± 5.4% of control (*p* < 0.05), whereas compound **4j** produced a more pronounced reduction to 72.1 ± 5.0% of control (*p* < 0.01). The strongest depolarization was observed with compound **3d**, which reduced mitochondrial membrane potential to 63.7 ± 4.8% of control (*p* < 0.001).

Importantly, the magnitude of mitochondrial depolarization paralleled the pattern observed in the preceding assays. Compound **3c**, showing the weakest apoptosis-associated and oxidative effects, caused only limited loss of ΔΨm, whereas compound **4j** and especially **3d** induced progressively greater mitochondrial depolarization. This graded relationship suggests that mitochondrial dysfunction is functionally associated with the broader oxidative and apoptotic response elicited by these derivatives. Mitochondrial membrane potential is essential for cellular bioenergetic balance, and its reduction reflects impairment of mitochondrial function. Since ΔΨm was evaluated at a single time point, the present data do not establish the temporal sequence between oxidative stress, mitochondrial depolarization, and cell death. Rather, the results demonstrate that mitochondrial dysfunction accompanies the cytotoxic effects induced by the selected compounds in A549 cells.

### 2.7. Cell Cycle Distribution

**Figure 8:**
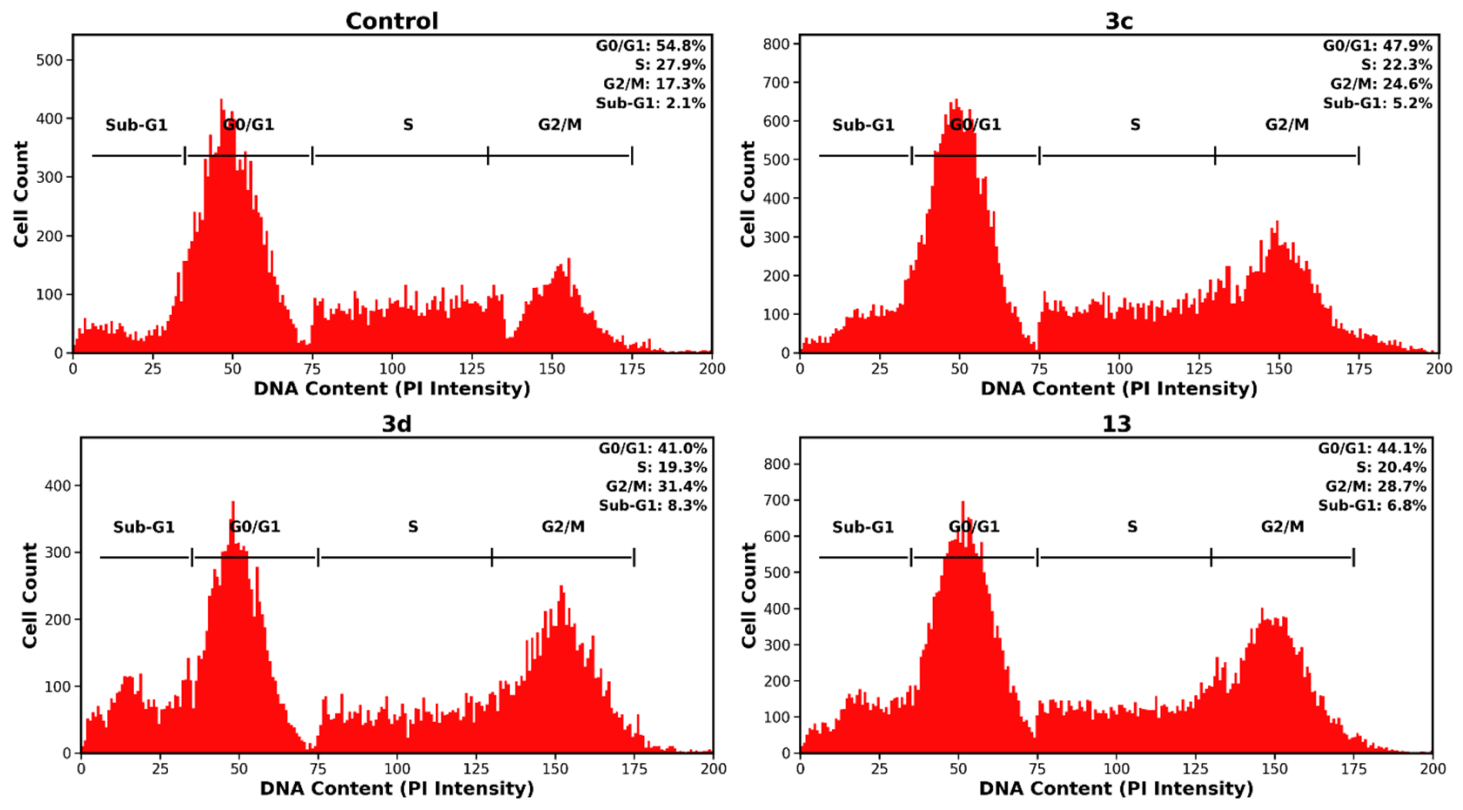
Flow cytometric profiling of cell cycle dynamics in human A549 adenocarcinoma cells treated with active derivatives. Representative DNA-content histograms depict the Sub-G1, G0/G1, S, and G2/M cell populations after a 48 h incubation with compounds **3c**, **3d**, and **4j** at IC_50_ concentrations followed by propidium iodide (PI) fluorometric staining.

To evaluate the impact of adenine–hydrazone derivatives on cell cycle progression, DNA content in A549 cells was quantified using propidium iodide (PI) staining and flow cytometry (Figure 8). In untreated control cultures, the majority of cells resided in the G0/G1 phase (54.8%), with 27.9% in S phase, 17.3% in G2/M phase, and a minimal Sub-G1 fraction (2.1%), reflecting a healthy and proliferating cell population. Exposure to compound **3c** induced a clear redistribution of the cell cycle profile, reducing the G0/G1 compartment to 47.9% while expanding the G2/M (24.6%) and Sub-G1 (5.2%) proportions. This effect was further pronounced following treatment with compound **4j**, which lowered the G0/G1 fraction to 44.1% alongside a marked elevation in both G2/M (28.7%) and Sub-G1 (6.8%) populations.

The most evident redistribution was detected following exposure to compound **3d**. In these cells, the G0/G1 fraction decreased to 41.0%, whereas the G2/M population increased to 31.4% and the Sub-G1 fraction reached 8.3%. This shift was accompanied by a relative reduction in the S phase (19.3%), indicating that the loss of proliferative cells was associated with cell cycle perturbation and accumulation of cells at the G2/M checkpoint. In sum, treatment with the selected synthesized derivatives was associated with cell cycle perturbation characterized by partial G2/M phase accumulation and an increased Sub-G1 population. The rise in the Sub-G1 fraction, which reflects cells with reduced DNA content, is compatible with DNA fragmentation occurring during regulated cell death. Importantly, these findings are consistent with the oxidative stress and redox imbalance data and support, rather than independently establish, the apoptosis-associated cytotoxic response.

### 2.8. Annexin V/PI Apoptosis Analysis

**Figure 9:**
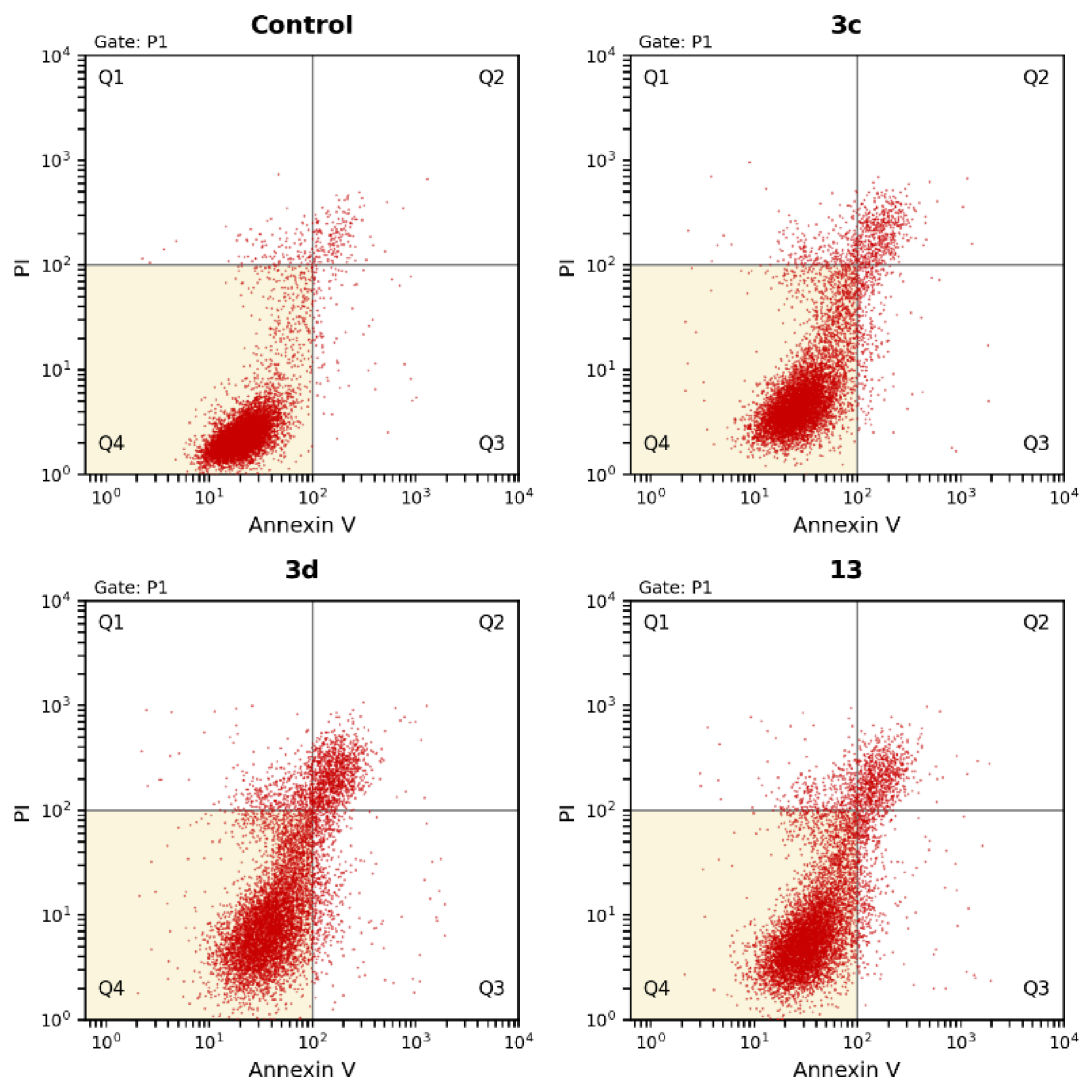
Flow cytometric Annexin V–FITC/propidium iodide (PI) double-staining dot plots illustrating apoptotic and necrotic cell distribution in A549 cells after treatment with compounds **3c**, **3d**, and **4j**. Cellular populations are categorized across four quadrants: viable (Annexin V^−^/PI^−^, **lower-left**), early apoptotic (Annexin V^+^/PI^−^, **lower-right**), late apoptotic (Annexin V^+^/PI^+^, **upper-right**), and necrotic (Annexin V^−^/PI^+^, **upper-left**). Relative to untreated controls, exposure to test derivatives induced a notable population transition from the viable quadrant into Annexin V-positive regions, with the predominant shift observed toward late apoptosis.

To further determine whether the loss of viability induced by the selected compounds was associated with apoptotic cell death, Annexin V/PI staining was performed in A549 cells following 48 h treatment at the respective IC_50_ concentrations (Figure 9). In control cells, the majority of the population remained viable (92.1 ± 2.6%), with only small fractions detected in the early apoptotic (3.2 ± 0.6%), late apoptotic/dead (2.9 ± 0.7%), and necrotic (1.8 ± 0.4%) quadrants. Treatment with compound 3c reduced the viable cell population to 76.9 ± 3.0% (*p* < 0.05) and increased both the early apoptotic (9.8 ± 1.0%, *p* < 0.05) and late apoptotic/dead (8.9 ± 1.0%, *p* < 0.01) fractions, while the necrotic population rose modestly to 4.4 ± 0.6% (*p* < 0.05).

A more pronounced apoptotic shift was observed following exposure to compound **4j**, which decreased the live-cell population to 68.7 ± 3.1% (*p* < 0.01) and increased early apoptotic and late apoptotic/dead cells to 12.5 ± 1.2% (*p* < 0.01) and 13.2 ± 1.3% (*p* < 0.01), respectively. The necrotic fraction also increased, reaching 5.6 ± 0.7% (*p* < 0.05). The most evident effect was detected with compound **3d**, where viable cells declined to 59.6 ± 2.8% (*p* < 0.001), while early apoptotic and late apoptotic/dead populations rose to 14.7 ± 1.3% (*p* < 0.01) and 18.9 ± 1.5% (*p* < 0.001), respectively. In the same group, the necrotic fraction increased to 6.8 ± 0.8% (*p* < 0.01).

Collectively, the tested synthesized analogues induced a marked redistribution from the viable quadrant toward predominantly early and late apoptotic populations, with only a moderate increase in necrotic cells. This pattern indicates that the cytotoxic effects of compounds **3c**, **3d**, and **4j** are driven primarily by apoptosis rather than primary necrotic injury. These findings align directly with the elevated Sub-G1 population, intracellular ROS generation, and redox imbalance observed in the preceding experiments.

### 2.9. In Silico Molecular Docking Studies

To provide a complementary structural framework for interpreting the in vitro biological activities of the synthesized derivatives, molecular docking simulations were performed for all newly synthesized compounds. The in vitro mechanistic assays revealed a dual-mode of action for the lead compounds, characterized by prominent G2/M cell cycle arrest and oxidative stress-mediated intrinsic apoptosis. Consequently, a multi-target in silico approach was systematically designed to explore potential theoretical interaction mechanisms underlying these phenotypic observations. Polo-like kinase 1 (PLK1, PDB: 2RKU), a master regulator of the G2/M transition, was selected to investigate the cell cycle arrest. To rationalize the profound intracellular redox imbalance and subsequent mitochondrial apoptotic cell death, Thioredoxin Reductase 1 (TrxR1, PDB: 2ZZC) and the MDM2-p53 complex (PDB: 4HG7) were evaluated as critical complementary targets. The binding affinities (docking scores, kcal/mol) of all compounds against these targets are summarized in Table 3.

Flow cytometric analysis demonstrated that treatment with compound **3d** led to a significant accumulation of A549 cells in the G2/M phase (31.4%). This experimentally observed G2/M accumulation was considered alongside the predicted PLK1 interactions. The most active derivatives within the series, **3d**, **3c**, and **4j**, showed favorable predicted PLK1 binding scores of −11.67, −11.66, and −11.78 kcal/mol, respectively (Table 3). Notably, compound **3f** displayed the most favorable calculated PLK1 docking score in the series (−13.20 kcal/mol), indicating strong predicted interactions within the kinase binding site. However, its antiproliferative activity in A549 cells was not the strongest among the tested derivatives, highlighting that docking affinity alone does not directly determine the cellular response. In contrast, the comparatively weak activity of compound **3g** was accompanied by a markedly less favorable PLK1 docking score (−6.48 kcal/mol). Taken together, these findings suggest that the docking results may provide useful structural context for the observed activity trends, while cellular potency is likely governed by multiple physicochemical and biological factors beyond predicted target affinity. Compound **4p** further illustrates this distinction, as its relatively favorable predicted binding scores were not accompanied by strong A549 antiproliferative activity or a favorable A549/MRC-5 selectivity profile, further emphasizing that docking scores should not be interpreted as direct surrogates of cellular potency or selectivity. Notably, compound **3f** displayed the highest theoretical affinity in the series (−13.20 kcal/mol) against PLK1. However, its moderate antiproliferative activity (IC50 = 59.4 ± 2.2 µM) demonstrates that high calculated binding affinity to an isolated target does not linearly translate to phenotypic cellular cytotoxicity. Such discrepancies highlight that molecular docking evaluates thermodynamic binding within a rigid, isolated protein pocket, whereas in vitro cytotoxicity is governed by complex cellular factors including aqueous solubility, membrane permeability, and intracellular drug accumulation.

Compound **3d** produced the highest intracellular ROS generation (228 ± 16% of control) together with the greatest disruption of the GSH/GSSG ratio (43.7% decrease), indicating pronounced oxidative and redox imbalance. Docking simulations targeting TrxR1 provided a plausible structural framework for interpreting these experimentally observed effects. Compound **4j** (−10.81 kcal/mol) and the 5-fluoro isatin derivative **3d** (−9.39 kcal/mol) showed favorable predicted accommodation within the TrxR1 active site. These predicted interactions provide a plausible structural context for the experimentally observed redox imbalance, although direct TrxR1 inhibition was not assessed experimentally.

To further explore the structural basis of the observed pro-apoptotic phenotype, docking against MDM2 (4HG7), a key negative regulator of p53, was performed. Compounds **3d** (−12.19 kcal/mol) and **3c** (−11.37 kcal/mol) showed favorable predicted binding profiles within the MDM2 binding site. These findings provide complementary structural information that may be relevant to apoptosis-associated pathways, although direct MDM2 inhibition or p53 stabilization was not experimentally assessed in the present study.

The docking profiles showed partial agreement with some experimental activity trends, particularly for compound **3g**, which exhibited both weak A549 activity and relatively unfavorable predicted binding scores across the evaluated targets. However, the discrepancies observed for compounds **3f** and **4p** indicate that predicted binding affinity does not directly translate into cellular antiproliferative activity or selectivity. Accordingly, the docking results are interpreted as complementary structural information within a broader physicochemical and biological context rather than as direct confirmation of a multi-target mechanism of action.

**Figure 10:**
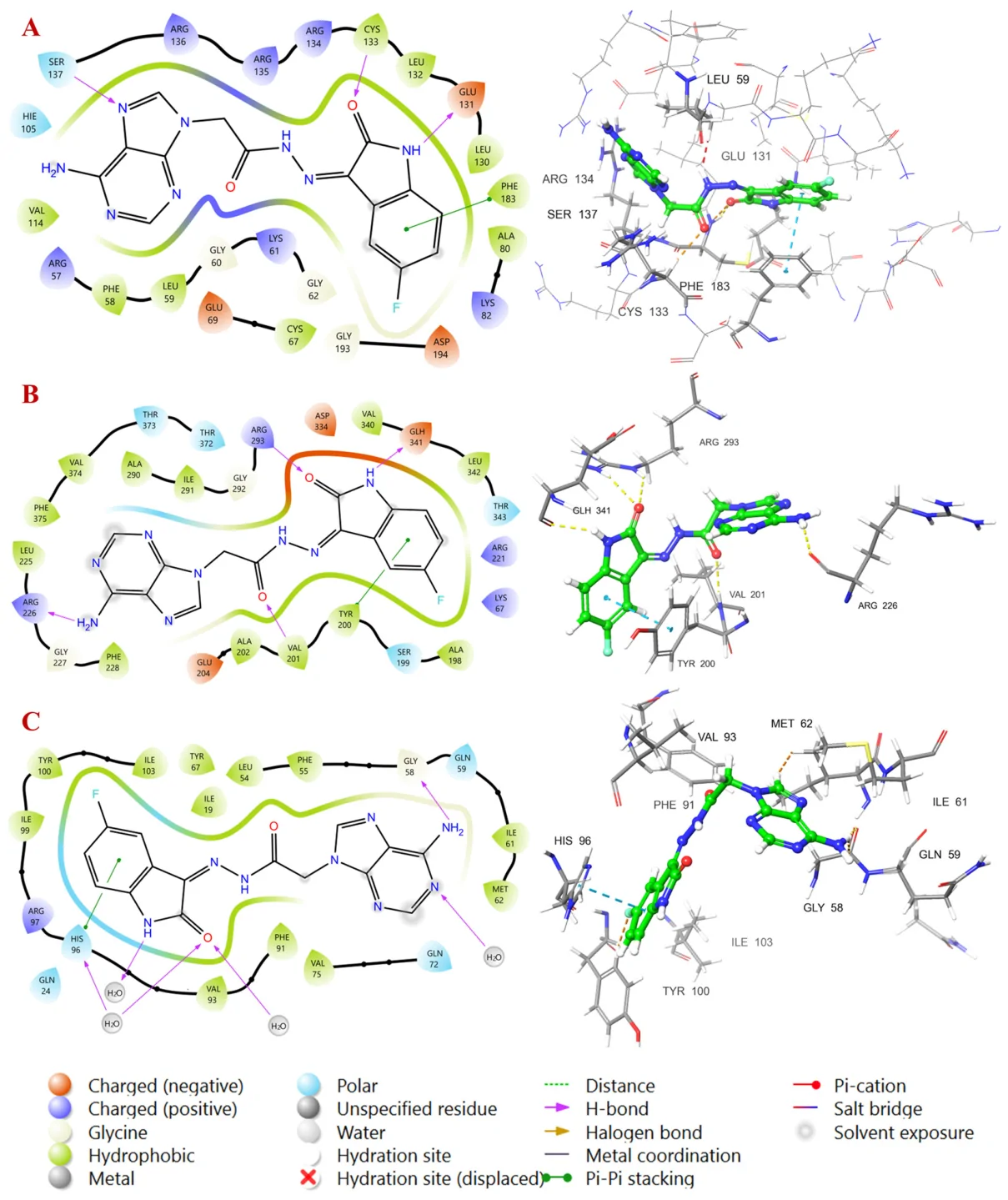
2D and 3D interaction diagrams of the compound **3d** within the active sites of the target proteins. (**A**) Binding pose in the ATP-binding pocket of PLK1. (**B**) Binding pose in the active site of TrxR1. (**C**) Binding pose in the p53-binding cleft of MDM2.

To gain further structural insight into the predicted binding modes of the synthesized series, the binding pose of the most active derivative, compound **3d**, was evaluated through a detailed visual analysis as the representative lead molecule. The two-dimensional (2D) and three-dimensional (3D) binding interaction maps of compound **3d** within the active sites of PLK1 (2RKU), TrxR1 (2ZZC), and MDM2 (4HG7) are illustrated in Figure 10. The binding orientation of compound **3d** within the PLK1 active site (Figure 10A) reveals a well-accommodated binding orientation, consistent with favorable occupancy of the kinase binding pocket. The adenine core deeply penetrates the hinge region, establishing two critical hydrogen bonds, one between the adenine amine group and the backbone of SER 137, and another between the adenine nitrogen and CYS 133. Furthermore, the isatin moiety is stabilized by a strong hydrogen bond formed between its NH group and the GLU 131 residue. The predicted binding mode is further supported by a pi-pi stacking interaction between the isatin aromatic ring and the PHE 183 residue. This network of polar and hydrophobic contacts is consistent with the calculated docking score and provides a possible structural context for the experimentally observed G2/M accumulation.

In the active site of the antioxidant enzyme TrxR1 (Figure 10B), compound **3d** adopts an extended conformation that effectively spans the binding pocket, positioned within the catalytic region of the enzyme. The hydrazone linker acts as a crucial structural bridge, participating in a robust hydrogen-bonding network with VAL 201 and protonated glutamic acid (GLH 341). Additionally, the isatin carbonyl oxygen acts as a hydrogen-bond acceptor for ARG 293, while the NH_2_ group of the adenine core interacts with ARG 226 as a hydrogen-bond donor. The isatin core is further anchored via a pi-pi stacking interaction with the TYR 200 residue. This extensive array of polar contacts provides structural context for the experimentally observed oxidative response.

The interaction map within the deeply hydrophobic p53-binding cleft of MDM2 (Figure 10C) highlights the structural adaptability of compound **3d**. The isatin core fits snugly into the sub-pocket, engaging in a crucial pi-pi stacking interaction with the HIS 96 residue, a known key contact point for p53 competitive inhibitors. The structural conformation is tightly held by a water-bridged hydrogen bond between the hydrazone carbonyl and the HIS 96 backbone. On the opposite end of the molecule, the adenine amine establishes a stable hydrogen bond with GLY 58. Furthermore, a complex water-bridged hydrogen bond network is observed surrounding the isatin and hydrazone domains, facilitating additional bridging interactions with the surrounding residues. This interaction network is consistent with the calculated binding affinity. It should be emphasized, however, that neither MDM2 binding nor p53 stabilization was assessed experimentally, and any contribution of this pathway to the apoptotic response observed in A549 cells remains hypothetical.

### 2.10. MD Simulation Analysis

To rigorously assess the time-dependent thermodynamic stability, conformational flexibility, and persistence of the predicted binding modes under physiological conditions, 100 ns Molecular Dynamics (MD) simulations were conducted for lead compound **3d** in complex with its three oncogenic target proteins: PLK1 (PDB ID: 2RKU), TrxR1 (PDB ID: 2ZZC), and MDM2 (PDB ID: 4HG7) using the Schrödinger Desmond platform. The resulting root-mean-square deviation (RMSD), 2D binding site topologies, and protein–ligand contact fraction histograms over the 100 ns trajectory are illustrated in Figure 10.

**Figure 11:**
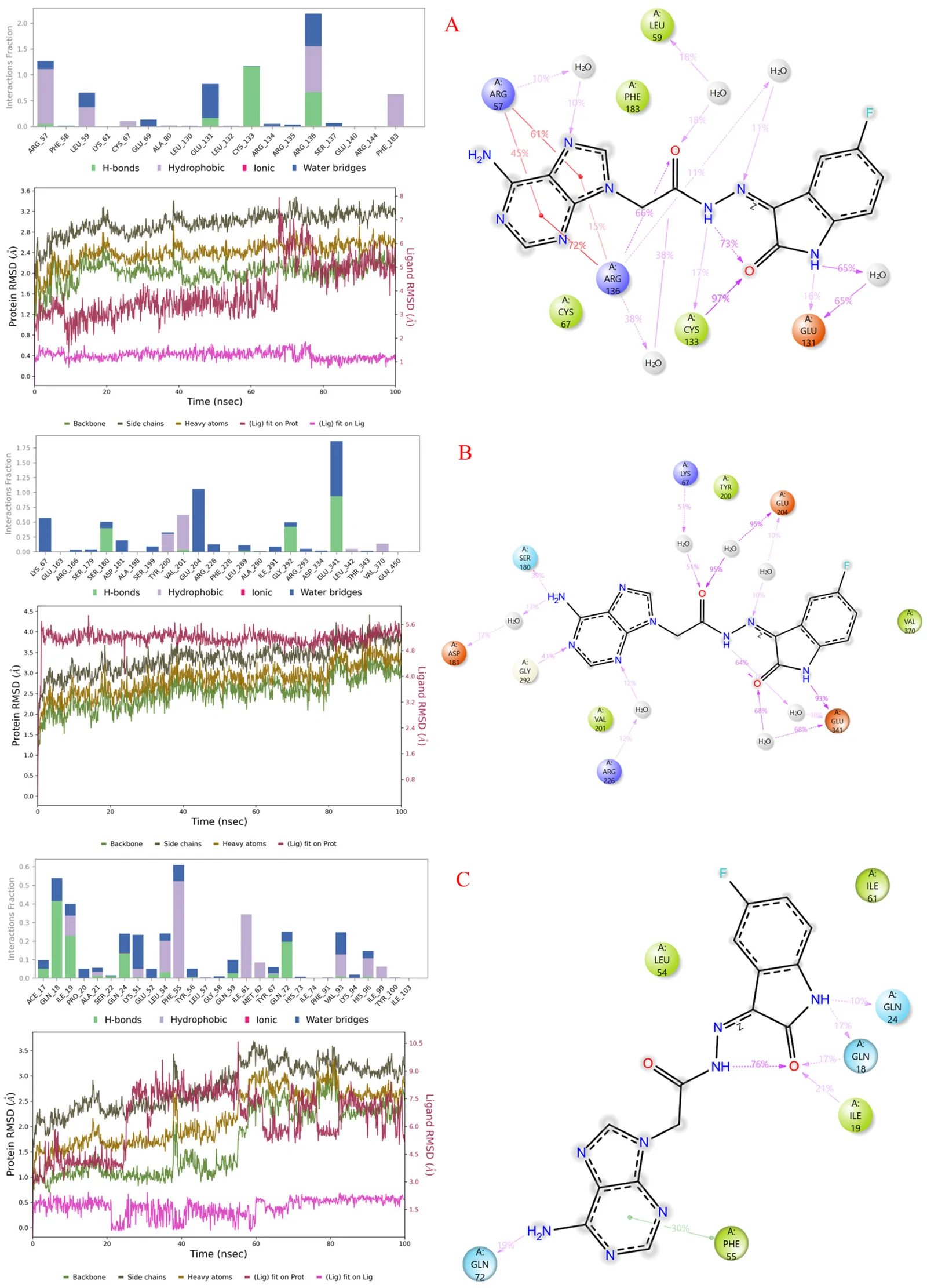
Molecular Dynamics (MD) simulation trajectory analysis of lead compound **3d** bound to PLK1 (**A**), TrxR1 (**B**), and MDM2 (**C**). For each panel: Left-up: Protein–ligand interaction fraction histograms categorizing hydrogen bonds (green), hydrophobic contacts (purple) and water bridges (blue) over the 100 ns simulation time. Right: 2D interaction schematics depicting key active-site amino acid contacts maintaining >10% trajectory fraction. Left-down: RMSD vs. time trajectory plots showing protein backbone (black/brown), side-chain (green), and ligand fit-on-protein (pink/purple) deviations in Angstroms (Å).

As depicted in Figure 11A, the compound **3d**–PLK1 complex exhibited rapid thermal equilibration within the initial 15 ns of the trajectory. The protein backbone RMSD plateaued tightly between 1.8 Å and 2.4 Å, while the ligand fit-on-protein RMSD remained exceptionally steady at 2.0–2.5 Å. Furthermore, the internal ligand heavy-atom RMSD (fit on Lig) maintained a near-constant trajectory at ~0.4 Å, confirming that compound **3d** undergoes negligible internal strain or structural distortion once bound inside the ATP-binding pocket. Interaction fraction profiling revealed that key hydrogen-bonding networks with hinge-region residues Cys133 and Arg136 were preserved for >70% of the total simulation time. These primary anchoring interactions were further stabilized by persistent water-mediated hydrogen bonds involving Glu131, Arg136, and Arg57, alongside π–cation interactions with Arg136, and Arg57.

For the compound **3d**–TrxR1 complex (Figure 11B), the protein backbone RMSD converged within 2.5–3.8 Å, whereas the ligand RMSD (fit on Prot) exhibited remarkable stability, remaining below 1.8 Å throughout the entire 100 ns simulation time. Contact fraction analysis highlighted an intense interaction network with Glu341 (exceeding a 150% cumulative contact fraction due to multi-atom ionic and hydrogen-bonding engagements), complemented by reliable hydrogen bonding and water bridges with Gly292, Ser180 and Glu204, as well as hydrophobic interactions with Val201 and Tyr200. Similarly, simulation of the compound **3d**–MDM2 complex (Figure 11C) demonstrated robust trajectory convergence, with protein backbone RMSD stabilizing between 1.5 Å and 3.0 Å and ligand position RMSD maintaining a steady plateau at 1.5–2.0 Å. Compound **3d** maintained stable polar interactions with key p53-binding pocket residues, including Gln18, Ile19, Gln24, and Gln72, reinforced by hydrophobic contacts with Phe55, Ile61, Val93, and His96. Collectively, the absence of ligand displacement or significant RMSD fluctuations (<2.5 Å) across all three targets confirms that compound **3d** forms thermodynamically stable, long-lived complexes.

### 2.11. In Silico ADMET and Druglikeness Profiling

To systematically evaluate the pharmacokinetic feasibility, membrane permeability, and safety profiles of the synthesized adenine–hydrazone derivatives, an in silico ADMET and druglikeness evaluation was performed using the Schrödinger QikProp module. The calculated descriptors alongside the recommended reference parameter ranges applicable to 95% of known oral drugs are summarized in Table 4.

As detailed in Table 4, all synthesized hybrids exhibited excellent overall druglikeness. The vast majority of the series completely satisfied Lipinski’s rule of five (RoF) with zero violations, whereas only three derivatives (**3f**, **3g**, and **4g**) displayed a single violation (RoF = 1), well within the acceptable drug-like threshold (RoF ≤ 1). The predicted octanol/water partition coefficients (logPo/w) ranged from 0.127 to 1.774, falling squarely within the recommended window (−2.0 to 6.5) and demonstrating optimal hydrophobic–hydrophilic equilibrium. Aqueous solubility predictions (logS) spanned from −5.640 to −3.148 mol/dm^3^, comfortably exceeding the critical insolvency threshold (−6.5 mol/dm^3^) and confirming appropriate thermodynamic solubility for systemic administration. Membrane transport and absorption profiling revealed significant structural differentiation between the two series. The arylidene-hydrazone series (**4–18**) demonstrated high gut–blood barrier permeability (PCaco = 154.2–199.6 nm/s, recommended: >500 nm/s high, 25–500 nm/s medium), translating into excellent predicted human oral absorption (PHO = 69.1–78.3%). In comparison, the lead 5-halo isatin derivatives **3c** (5-Cl) and **3d** (5-F) exhibited moderate Caco-2 permeability (PCaco = 48.87 nm/s) and solid oral absorption values of 64.86% and 63.44%, respectively (recommended: >80% high, 25–80% moderate). MDCK cell permeability values (PMDCK), serving as an in silico model for trans-epithelial transport, further supported this trend (PMDCK = 34.12–317.30 nm/s for bioactive derivatives). Crucially, all compounds displayed strongly negative blood–brain barrier partition coefficients (logBB ranging from −3.076 to −1.005, recommended: −3.0 to 1.2), indicating low central nervous system (CNS) penetration and minimal risk of neurotoxic off-target effects. Serum albumin binding predictions (logKhsa = −0.540 to −0.198, recommended: −1.5 to 1.5) confirmed appropriate free plasma fraction availability. Furthermore, cardiac potassium channel inhibition risks (logHERG) were within acceptable safety boundaries, particularly for the arylidene derivatives (−3.927 to −4.486). In summary, lead candidates **3c**, **3d**, and **4j** combine favorable oral bioavailability profiles with favorable ADMET safety margins, reinforcing their potential for further drug optimization.

## 3. Materials and Methods

### 3.1. Chemistry

All reagents, chemicals, and solvents utilized in this study were of analytical grade and purchased from commercial suppliers (Sigma-Aldrich, St. Louis, MO, USA; Merck, Darmstadt, Germany; and Fluka, Buchs, Switzerland) and utilized without further purification. Melting points were determined using an automated capillary melting point apparatus (Gallenkamp, London, UK) and are uncorrected. Fourier Transform Infrared (FT-IR) spectra were recorded on a PerkinElmer Spectrum 100 FT-IR spectrometer (PerkinElmer, Waltham, MA, USA) utilizing attenuated total reflectance (ATR) sampling. Nuclear Magnetic Resonance (^1^H-NMR and ^13^C-NMR) spectra were recorded on a Bruker 400 MHz spectrometer (Bruker, Billerica, MA, USA) in DMSO-*d*_6_ with tetramethylsilane (TMS) as the internal standard. Chemical shifts (δ) are reported in parts per million (ppm) and coupling constants (J) in Hertz (Hz). Elemental analyses (C, H, N, S) were carried out using a Thermo Scientific FLASH 2000 CHNS analyzer (Thermo Fisher Scientific, Waltham, MA, USA).

#### 3.1.1. Synthesis of 2-(6-Amino-9H-purin-9-yl)-*N*′-(2-oxoindolin-3-ylidene)acetohydrazide Derivatives (**3a–g**)

Compound 2 (2-(6-amino-9H-purin-9-yl)acetohydrazide, 0.01 mol) was continuously stirred in dry ethanol (20 mL) for 15 min at room temperature. Then, the appropriate isatin derivative (0.01 mol) and 5 drops of glacial acetic acid, acting as a catalyst, were added to the mixture. The reaction mixture was heated to reflux and boiled for 8 h. After completion (monitored by TLC), the mixture was cooled to room temperature and poured into ice-water (100 mL). The resulting precipitate was collected by filtration, washed thoroughly with cold ethanol and water, and dried in a vacuum desiccator to yield the corresponding pure product.

2-(6-Amino-9*H*-purin-9-yl)-*N*′-(2-oxoindolin-3-ylidene)acetohydrazide (**3a**)

Yield: 90%, mp.: 302–303 °C, IR (νmax/cm^−1^): 3303, 3269 (NH–NH_2_), 1688, 1663 (C=O), 1607, 1575 (C=N). ^1^H-NMR (400 MHz, DMSO-*d*_6_), δ, ppm: 10.84 (1H, s, NH), 9.44 (1H, s, NH), 8.14–8.05 (4H, m, ArH), 7.27 (2H, s, NH_2_), 7.04 (1H, s, ArH), 6.90 (1H, s, ArH), 4.77 (2H, s, NCH_2_). ^13^C APT (100MHz, DMSO-*d*_6_), δ, ppm: 44.07 (NCH_2_), 111.04, 115.28, 118.48, 122.32, 126.55, 141.02, 144.89 (Ar–C), 150.21 (C=N), 152.89 (Ar–C), 156.56 (C=N), 166.11, 170.35 (C=O). Anal. Calcd. for C_15_H_12_N_8_O_2_: C, 53.57%; H, 3.60%; N, 33.32%; Found: C, 53.65%; H, 3.66%; N, 33.39%.

2-(6-Amino-9*H*-purin-9-yl)-*N*′-(5-bromo-2-oxoindolin-3-ylidene)acetohydrazide (**3b**)

Yield: 91%, mp.: 315–317 °C, IR (νmax/cm^−1^): 3332, 3139 (NH–NH_2_), 1697, 1680 (C=O), 1607 (C=N). ^1^H-NMR (400 MHz, DMSO-*d*_6_), δ, ppm: 13.08 and 12.59 (1H, s, NH), 11.43 (1H, s, NH), 8.14 (2H, d, *J* = 12.0 Hz, ArH), 7.81 (1H, s, ArH), 7.57 (1H, s, ArH), 7.31 (2H, s, NH_2_), 6.94 (1H, s, ArH), 5.90 + 5.26 (2H, s, NCH_2_, *cis/trans* amide conformers, 80/20 ratio). ^13^C APT (100 MHz, DMSO-*d*_6_), δ, ppm: 44.08 (NCH_2_), 113.63, 114.89, 118.63, 122.02, 123.77, 134.21, 142.04 (Ar–C), 150.16 (C=N), 152.89 (N=CH), 156.27 (C=N), 162.38, 169.53 (C=O). Anal. Calcd. for C_15_H_11_BrN_8_O_2_: C, 43.39%; H, 2.67%; N, 26.99%; Found: C, 43.50%; H, 2.70%; N, 27.08%.

2-(6-Amino-9*H*-purin-9-yl)-*N*′-(5-chloro-2-oxoindolin-3-ylidene)acetohydrazide (**3c**)

Yield: 90%, mp.: 313–314 °C, IR (νmax/cm^−1^): 3332, 3146 (NH–NH_2_), 1722, 1701 (C=O), 1611 (C=N). ^1^H-NMR (400 MHz, DMSO-*d*_6_), δ, ppm: 13.05 and 12.60 (1H, s, NH), 11.44 (1H, s, NH), 8.14 (2H, d, *J* = 8.0 Hz, ArH), 7.71 (1H, s, ArH), 7.45 (1H, s, ArH), 7.30 (2H, s, NH_2_), 7.00 (1H, s, ArH), 5.60 + 5.24 (2H, s, NCH_2_, *cis/trans* amide conformers, 79.5/20.5 ratio). ^13^C APT (100 MHz, DMSO-*d*_6_), δ, ppm: 44.10 (NCH_2_), 113.23, 118.62, 120.67, 121.74, 127.44, 131.51, 134.90, 141.36 (Ar–C), 150.19 (C=N), 153.26 (N=CH), 156.27 (C=N), 162.75, 169.82 (C=O). Anal. Calcd. for C_15_H_11_ClN_8_O_2_: C, 48.59%; H, 2.99%; N, 30.22%; Found: C, 48.70%; H, 3.03%; N, 30.30%.

2-(6-Amino-9*H*-purin-9-yl)-*N*′-(5-fluoro-2-oxoindolin-3-ylidene)acetohydrazide (**3d**)

Yield: 91%, mp.: 313–314 °C, IR (νmax/cm^−1^): 3329, 3138 (NH–NH_2_), 1682, 1621 (C=O), 1597 (C=N). ^1^H-NMR (400 MHz, DMSO-*d*_6_), δ, ppm: 13.11 and 12.66 (1H, s, NH), 11.34 (1H, s, NH), 8.14 (2H, d, *J* = 8.0 Hz, ArH), 7.51 (1H, s, ArH), 7.30 (3H, s, ArH and NH_2_), 6.98 (1H, s, ArH), 5.63 + 5.27 (2H, s, NCH_2_, *cis/trans* amide conformers, 80/20 ratio). ^13^C APT (100 MHz, DMSO-*d*_6_), δ, ppm: 43.79 (NCH_2_), 108.55 (d, *J =* 26 Hz), 112.81 (d, *J =* 8 Hz), 118.69 (d, *J =* 25), 118.75, 135.50, 139.41, 142.10 (Ar–C), 150.131 (C=N), 153.01 (N=CH), 156.40 (C=N), 158.78 (CF, d, *J =* 237 Hz), 162.99, 169.49 (C=O). ^19^F NMR (376 MHz, DMSO-*d*_6_), δ, ppm: −120.45. Anal. Calcd. for C_15_H_11_FN_8_O_2_: C, 50.85%; H, 3.13%; N, 31.63%; Found: C, 50.98%; H, 3.17%; N, 31.71%.

2-(6-Amino-9H-purin-9-yl)-*N*′-(5-methyl-2-oxoindolin-3-ylidene)acetohydrazide (**3e**)

Yield: 93%, mp.: 305–308 °C, IR (νmax/cm^−1^): 3330, 3137 (NH–NH_2_), 1718, 1681 (C=O), 1603 (C=N). ^1^H-NMR (400 MHz, DMSO-*d*_6_), δ, ppm: 13.11 and 12.70 (1H, s, NH), 11.24 (1H, s, NH), 8.14 (2H, d, *J* = 8.0 Hz, ArH), 7.47 (1H, s, ArH), 7.30 (2H, s, NH_2_), 7.22 (1H, s, ArH), 6.86 (1H, s, ArH), 5.98 + 5.21 (2H, s, NCH_2_, *cis/trans* amide conformers, 75/25 ratio), 2.32 (3H, s, CH_3_). ^13^C APT (100 MHz, DMSO-*d*_6_), δ, ppm: 21.02 (CH_3_), 43.78 (NCH_2_), 111.50, 118.63, 119.99, 121.05, 131.88, 132.85, 141.06 (Ar–C), 150.18 (C=N), 152.88 (N=CH), 156.26 (C=N), 163.03, 169.53 (C=O). Anal. Calcd. for C_16_H_14_N_8_O_2_: C, 54.85%; H, 4.03%; N, 31.98%; Found: C, 54.98%; H, 4.07%; N, 32.07%.

2-(6-Amino-9*H*-purin-9-yl)-*N*′-(5-nitro-2-oxoindolin-3-ylidene)acetohydrazide (**3f**)

Yield: 85%, mp.: 274–276 °C, IR (νmax/cm^−1^): 3333, 3136 (NH–NH_2_), 1697 (C=O), 1602 (C=N). ^1^H-NMR (400 MHz, DMSO-*d*_6_), δ, ppm: 12.48 (1H, s, NH), 11.91 (1H, s, NH), 8.44–8.31 (2H, m, ArH), 8.14 (1H, s, ArH), 8.11 (1H, s, ArH), 7.25 (2H, s, NH_2_), 7.15 (1H, d, *J* = 4.0 Hz, ArH), 5.63 (2H, s, NCH_2_). ^13^C APT (100 MHz, DMSO-*d*_6_), δ, ppm: 40.85 (NCH_2_), 112.14, 116.37, 118.78, 120.60, 127.97, 143.48, 147.79 (Ar–C), 150.21 (C=N), 156.56 (C=N), 165.73, 170.34 (C=O). Anal. Calcd. for C_15_H_11_N_9_O_4_: C, 47.25%; H, 2.91%; N, 33.06%; Found: C, 47.36%; H, 2.95%; N, 33.13%.

2-(6-Amino-9*H*-purin-9-yl)-*N*′-(5-methoxy-2-oxoindolin-3-ylidene)acetohydrazide (**3g**)

Yield: 87%, mp.: 319–321 °C, IR (νmax/cm^−1^): 3332, 3138 (NH–NH_2_), 1698 (C=O), 1605 (C=N). ^1^H-NMR (400 MHz, DMSO-*d*_6_), δ, ppm: 13.17 and 12.73 (1H, s, NH), 11.16 (1H, s, NH), 8.17 (2H, d, *J* = 12.0 Hz, ArH), 7.47 (2H, s, NH_2_), 7.23 (1H, s, ArH), 6.98 (1H, s, ArH), 6.90 (1H, s, ArH), 5.61 + 5.27 (2H, s, NCH_2_, *cis/trans* amide conformers, 75/25 ratio), 3.78 (3H, s, OCH_3_). ^13^C APT (100 MHz, DMSO-*d*_6_), δ, ppm: 43.78 (NCH_2_), 55.99 (OCH_3_), 112.57, 114.74, 118.56, 127.74, 137.31, 140.45, 145.60 (Ar–C), 150.18 (C=N), 152.47 (N=CH), 155.99 (C=N), 163.56, 169.41 (C=O). Anal. Calcd. for C_16_H_14_N_8_O_3_: C, 52.46%; H, 3.85%; N, 30.59%; Found: C, 52.59%; H, 3.89%; N, 30.65%.

#### 3.1.2. Synthesis of Aldehyde-Derived Hydrazones (**4a–p**)

Compound 2 (0.01 mol) was continuously stirred in dry ethanol (20 mL) for 15 min at room temperature. Then, the appropriate aldehyde derivative (0.01 mol) and 5 drops of glacial acetic acid catalyst were added. The reaction mixture was boiled under reflux for 8 h. After completion (monitored by TLC), the mixture was cooled to room temperature and poured into water (100 mL). The resulting precipitate was collected by filtration, washed with cold ethanol, and dried in a vacuum desiccator to yield the corresponding Schiff base.

2-(6-Amino-9*H*-purin-9-yl)-*N*′-(2-chlorobenzylidene)acetohydrazide (**4a**)

Yield: 89%, m.p.: 306–307 °C, IR (νmax, cm^−1^): 3276, 3111 (NH–NH_2_), 1674 (C=O), 1602, 1573 (C=N). ^1^H-NMR (400 MHz, DMSO-*d*_6_), δ, ppm: 12.15 and 12.00 (1H, s, NH), 8.63 + 8.45 (1H, s, N=CH, *E*/*Z* amide conformer, 79/21 ratio), 8.12 (3H, s, ArH), 7.55 (3H, d, *J* = 8.0 Hz, ArH), 7.46 (2H, t, *J* = 8.0 Hz, ArH), 7.27 (2H, s, NH_2_), 5.45 + 5.01 (2H, s, NCH_2_, *cis/trans* amide conformers, 80/20 ratio). ^13^C APT (100 MHz, DMSO-*d*_6_), δ, ppm: 44.45 (NCH_2_), 119.01, 127.42, 130.55, 131.44, 132.00, 133.24, 141.07 (Ar–C), 143.78 (N=CH), 150.55 (C=N), 152.92 (N=CH), 156.25 (C=N), 168.83 (C=O). Anal. Calcd. for C_14_H_12_ClN_7_O: C, 50.99%; H, 3.67%; N, 29.73%; Found: C, 51.12%; H, 3.70%; N, 29.80%.

2-(6-Amino-9*H*-purin-9-yl)-*N*′-(2-methylbenzylidene)acetohydrazide (**4b**)

Yield: 91%, m.p.: 288–289 °C, IR (νmax/cm^−1^): 3274, 3106 (NH–NH_2_), 1674 (C=O), 1606, 1573 (C=N). ^1^H-NMR (400 MHz, DMSO-*d*_6_), δ, ppm: 11.87 and 11.72 (1H, s, NH), 8.48 + 8.31 (1H, s, N=CH, *E*/*Z* amide conformer, 75/25 ratio), 8.10 (2H, s, ArH), 7.80 (1H, d, *J* = 8.0 Hz, ArH), 7.24 (5H, s, ArH and NH_2_), 5.38 + 4.97 (2H, s, NCH_2_, *cis/trans* amide conformers, 75/25 ratio). ^13^C APT (100 MHz, DMSO-*d*_6_), δ, ppm: 19.71 (CH_3_), 44.41 (NCH_2_), 118.80, 126.94, 130.09, 131.46, 132.20, 137.13, 143.87 (Ar–C), 146.30 (N=CH), 150.20 (C=N), 152.72 (N=CH), 156.25 (C=N), 168.23 (C=O). Anal. Calcd. for C_15_H_15_N_7_O: C, 58.24%; H, 4.89%; N, 31.70%; Found: C, 58.37%; H, 4.93%; N, 31.80%.

2-(6-Amino-9*H*-purin-9-yl)-*N*′-(2,5-dichlorobenzylidene)acetohydrazide (**4c**)

Yield: 87%, m.p.: 319–320 °C, IR (νmax/cm^−1^): 3283, 3079 (NH–NH_2_), 1683 (C=O), 1646, 1597, 1577 (C=N). ^1^H-NMR (400 MHz, DMSO-*d*_6_), δ, ppm: 12.25 and 12.06 (1H, s, NH), 8.57 + 8.39 (1H, s, N=CH, *E*/*Z* amide conformer, 80/20 ratio), 8.12 (3H, s, ArH), 7.61–7.52 (2H, m, ArH), 7.26 (2H, s, NH_2_), 5.48 + 5.02 (2H, s, NCH_2_, *cis/trans* amide conformers, 80/20 ratio). ^13^C APT (100 MHz, DMSO-*d*_6_), δ, ppm: 44.46 (NCH_2_), 119.01, 126.46, 131.49, 132.06, 132.15, 133.04, 133.36 (Ar–C), 139.33 (N=CH), 150.56 (C=N), 152.91 (N=CH), 156.27 (C=N), 169.14 (C=O). Anal. Calcd. for C_14_H_11_Cl_2_N_7_O: C, 46.17%; H, 3.04%; N, 26.92%; Found: C, 46.29%; H, 3.08%; N, 26.99%.

2-(6-Amino-9*H*-purin-9-yl)-*N*′-(2,6-dichlorobenzylidene)acetohydrazide (**4d**)

Yield: 85%, m.p.: 312–313 °C, IR (νmax/cm^−1^): 3319, 3208 (NH–NH_2_), 1682 (C=O), 1643, 1599 (C=N). ^1^H-NMR (400 MHz, DMSO- *d*_6_), δ, ppm: 12.20 and 12.08 (1H, s, NH), 8.42 + 8.33 (1H, s, N=CH, *E*/*Z* amide conformer, 80/20 ratio), 8.11 (2H, s, ArH), 7.60 (2H, d, *J* = 12.0 Hz, ArH), 7.46 (1H, t, *J* = 12.0 Hz, ArH), 7.25 (2H, s, NH_2_), 5.34 + 5.03 (2H, s, NCH_2_, *cis/trans* amide conformers, 80/20 ratio). ^13^C APT (100 MHz, DMSO-*d*_6_), δ, ppm: 44.09 (NCH_2_), 118.64, 130.13, 131.87, 134.57 (Ar–C), 139.95 (N=CH), 150.55 (C=N), 152.87 (N=CH), 156.64 (C=N), 168.84 (C=O). Anal. Calcd. for C_14_H_11_Cl_2_N_7_O: C, 46.17%; H, 3.04%; N, 26.92%; Found: C, 46.28%; H, 3.07%; N, 27.01%.

2-(6-Amino-9*H*-purin-9-yl)-*N*′-(3-chlorobenzylidene)acetohydrazide (**4e**)

Yield: 89%, m.p.: 292–293 °C, IR (νmax/cm^−1^): 3332, 3161 (NH–NH_2_), 1692 (C=O), 1606, 1573 (C=N). ^1^H-NMR (400 MHz, DMSO-*d*_6_), δ, ppm: 12.05 and 11.92 (1H, s, NH), 8.23 + 8.06 (1H, s, N=CH, *E*/*Z* amide conformer, 80/20 ratio), 8.12 (2H, s, ArH), 7.87 (1H, s, ArH), 7.71 (1H, s, ArH), 7.50 (2H, s, ArH), 7.26 (2H, s, NH_2_), 5.45 + 5.01 (2H, s, NCH_2_, *cis/trans* amide conformers, 80/20 ratio). ^13^C APT (100 MHz, DMSO-*d*_6_), δ, ppm: 44.90 (NCH_2_), 118.62, 126.35, 126.52, 130.21, 131.20, 134.20, 136.59 (Ar–C), 143.40 (N=CH), 150.55 (C=N), 152.90 (N=CH), 156.27 (C=N), 168.87 (C=O). Anal. Calcd. for C_14_H_12_ClN_7_O: C, 50.99%; H, 3.67%; N, 29.73%; Found: C, 51.11%; H, 3.70%; N, 29.81%.

2-(6-Amino-9*H*-purin-9-yl)-*N*′-(3-methylbenzylidene)acetohydrazide (**4f**)

Yield: 88%, m.p.: 301–303 °C, IR (νmax/cm^−1^): 3333, 3161 (NH–NH_2_), 1689 (C=O), 1609, 1573 (C=N). ^1^H-NMR (400 MHz, DMSO-*d*_6_), δ, ppm: 11.90 and 11.79 (1H, s, NH), 8.18 + 8.04 (1H, s, N=CH, *E*/*Z* amide conformer, 75/25 ratio), 8.12 (2H, s, ArH), 7.59 (1H, s, ArH), 7.54 (1H, d, *J* = 8.0 Hz, ArH), 7.35 (1H, t, *J* = 12.0 Hz, ArH), 7.26 (3H, s, ArH and NH_2_), 5.42 + 4.99 (2H, s, NCH_2_, *cis/trans* amide conformers, 75/25 ratio), 2.34 (3H, s, CH_3_). ^13^C APT (100 MHz, DMSO-*d*_6_), δ, ppm: 21.36 (CH_3_), 44.45 (NCH_2_), 118.65, 124.75, 127.74, 129.23, 131.30, 134.21, 138.61, 145.13 (Ar–C), 148.11 (N=CH), 150.56 (C=N), 152.89 (N=CH), 156.66 (C=N), 168.85 (C=O). Anal. Calcd. for C_15_H_15_N_7_O: C, 58.24%; H, 4.89%; N, 31.70%; Found: C, 58.39%; H, 4.92%; N, 31.80%.

2-(6-Amino-9*H*-purin-9-yl)-*N*′-(3-nitrobenzylidene)acetohydrazide (**4g**)

Yield: 92%, m.p.: 314–316 °C, IR (νmax/cm^−1^): 3313, 3209 (NH–NH_2_), 1692 (C=O), 1647, 1595 (C=N). ^1^H-NMR (400 MHz, DMSO-*d*_6_), δ, ppm: 12.18 and 12.05 (1H, s, NH), 8.57 (1H, s, ArH), 8.38 + 8.28 (1H, s, N=CH, *E*/*Z* amide conformer, 71/29 ratio), 8.22 (2H, t, *J* = 8.0 Hz, ArH), 8.12 (2H, s, ArH), 7.76 (1H, t, *J* = 12.0 Hz, ArH), 7.26 (2H, s, NH_2_), 5.48 + 5.03 (2H, s, NCH_2_, cis/trans amide conformers, 74/26 ratio).^13^C APT (100 MHz, DMSO-*d*_6_), δ, ppm: 44.47 (NCH_2_), 118.62, 121.35, 124.75, 130.87, 133.23, 135.95, 142.42, 145.43 (Ar–C), 148.52 (N=CH), 150.55 (C=N), 152.92 (N=CH), 156.65 (C=N), 168.85 (C=O). Anal. Calcd. for C_14_H_12_N_8_O_3_: C, 49.41%; H, 3.55%; N, 32.93%; Found: C, 49.55%; H, 3.58%; N, 33.01%. 12.18, 12.05, 8.57, 8.38, 8.28, 8.25, 8.23, 8.20, 8.12, 7.78, 7.76, 7.74, 7.26, 5.48, 5.01.

2-(6-Amino-9*H*-purin-9-yl)-*N*′-(3-hydroxybenzylidene)acetohydrazide (**4h**)

Yield: 91%, m.p.: 309–310 °C, IR (νmax/cm^−1^): 3301, 3145 (NH–NH_2_), 1673 (C=O), 1645, 1594 (C=N). ^1^H-NMR (400 MHz, DMSO-*d*_6_), δ, ppm: 11.87 and 11.77 (1H, s, NH), 9.67 (1H, s, OH), 8.12 (2H, s, N=CH, *E*/*Z* amide conformer and ArH),7.99 (1H, s, ArH), 7.27 (3H, s, ArH and NH_2_), 7.15 (2H, d, *J* = 8.0 Hz, ArH), 6.85 (1H, d, *J* = 8.0 Hz, ArH), 5.41 + 4.99 (2H, s, NCH_2_, *cis/trans* amide conformers, 80/20 ratio). ^13^C-NMR (100 MHz, DMSO-*d*_6_), δ, ppm: 44.09 (NCH_2_), 113.17, 118.01, 118.58, 118.99, 130.52, 136.59, 145.04 (Ar-C), 148.02 (N=CH), 150.36 (C=N), 152.91 (N=CH), 156.40 (C=N), 158.15 (Ar–C), 168.17 (C=O). Anal. Calcd. for C_14_H_13_N_7_O_2_: C, 54.02%; H, 4.21%; N, 31.50%; Found: C, 54.21%; H, 4.25%; N, 31.59%.

2-(6-Amino-9*H*-purin-9-yl)-*N*′-(thiophen-2-ylmethylene)acetohydrazide (**4i**)

Yield: 89%, m.p.: 311–313 °C, IR (νmax/cm^−1^): 3304, 3133 (NH–NH_2_), 1657 (C=O), 1599, 1588 (C=N). ^1^H-NMR (400 MHz, DMSO-*d*_6_), δ, ppm: 9.43 (1H, s, NH), 8.42 + 8.23 (1H, s, N=CH, *E*/*Z* amide conformer, 75/25 ratio), 8.09- 8.05 (3H, d, *J* = 16.0 Hz, ArH), 7.20–7.13 (4H, m, NH_2_ and ArH), 5.30 + 4.94 (2H, s, NCH_2_, *cis/trans* amide conformers, 75/25 ratio). ^13^C APT (100 MHz, DMSO-*d*_6_), δ, ppm: 44.38 (NCH_2_), 118.80, 128.36, 129.06, 131.18, 138.94 (Ar-C), 139.98 (N=CH), 149.50 (C=N), 153.04 (N=CH), 156.56 (C=N), 166.43 (Ar–C), 168.23 (C=O). Anal. Calcd. for C_12_H_11_N_7_OS: C, 47.83%; H, 3.68%; N, 32.54%; S, 10.64%; Found: C, 47.98%; H, 3.71%; N, 32.65%; S, 10.71%.

2-(6-Amino-9*H*-purin-9-yl)-*N*′-(4-methylbenzylidene)acetohydrazide (**4j**)

Yield: 93%, m.p.: 305–307 °C, IR (νmax/cm^−1^): 3334, 3163 (NH–NH_2_), 1675 (C=O), 1603, 1570 (C=N). ^1^H-NMR (400 MHz, DMSO-*d*_6_), δ, ppm: 11.85 and 11.75 (1H, s, NH), 8.12 (2H, s, N=CH, *E*/*Z* amide conformer and ArH), 8.04 (1H, s, ArH), 7.65 (2H, d, *J* = 8.0 Hz, ArH), 7.27 (4H, s, ArH and NH_2_), 5.41 + 4.98 (2H, s, NCH_2_, *cis/trans* amide conformers, 75/25 ratio), 2.35 (3H, s, CH_3_). ^13^C APT (100 MHz, DMSO-*d*_6_), δ, ppm: 21.35 (CH_3_), 44.41 (NCH_2_), 119.00, 127.42, 130.15, 131.50, 140.41, 144.75 (Ar–C), 147.84 (N=CH), 150.56 (C=N), 152.89 (N=CH), 156.28 (C=N), 168.52 (C=O). Anal. Calcd. for C_15_H_15_N_7_O: C, 58.24%; H, 4.89%; N, 31.70%; Found: C, 58.38%; H, 4.93%; N, 31.78%.

2-(6-Amino-9*H*-purin-9-yl)-*N*′-(pyridin-2-ylmethylene)acetohydrazide (**4k**)

Yield: 83%, m.p.: 304–306 °C, IR (νmax/cm^−1^): 3339, 3131 (NH–NH_2_), 1685 (C=O), 1606, 1587 (C=N). ^1^H-NMR (400 MHz, DMSO-*d*_6_), δ, ppm: 12.09 and 11.97 (1H, s, NH), 8.59 + 8.23 (1H, s, N=CH, *E*/*Z* amide conformer, 81/19 ratio), 8.10 (3H, d, *J* = 8.0 Hz, ArH), 8.02 (1H, d, *J* = 8.0 Hz, ArH), 7.87 (1H, d, *J* = 8.0 Hz, ArH), 7.41 (1H, t, *J* = 8.0 Hz, ArH), 7.24 (2H, s, NH_2_), 5.44 + 5.01 (2H, s, NCH_2_, *cis/trans* amide conformers, 80/20 ratio).^13^C-NMR (100 MHz, DMSO-*d*_6_), δ, ppm: 44.39 (NCH_2_), 118.82, 120.21, 124.83, 136.82, 145.26, 148.10 (Ar-C), 149.98 (N=CH), 150.43 (C=N), 152.89 (N=CH), 153.04 (Ar–C), 156.25 (C=N), 168.94 (C=O). Anal. Calcd. for C_13_H_12_N_8_O: C, 52.70%; H, 4.08%; N, 37.82%; Found: C, 52.84%; H, 4.11%; N, 37.91%.

2-(6-Amino-9*H*-purin-9-yl)-*N*′-(4-hydroxybenzylidene)acetohydrazide (**4l**)

Yield: 87%, m.p.: 285–287 °C, IR (νmax/cm^−1^): 3220, 3076 (NH–NH_2_), 1674 (C=O), 1604, 1580 (C=N). ^1^H-NMR (400 MHz, DMSO-*d*_6_), δ, ppm: 11.70 and 11.61 (1H, s, NH), 9.96 (1H, s, H, OH), 8.12 (2H, s, N=CH, *E*/*Z* amide conformer and ArH), 7.97 (1H, s, ArH), 7.59 (1H, d, *J* = 8.0 Hz, ArH), 7.25 (2H, s, NH_2_), 6.84 (2H, d, *J* = 8.0 Hz, ArH), 5.38 + 4.96 (2H, s, NCH_2_, cis/trans amide conformers, 80/20 ratio). ^13^C APT (100 MHz, DMSO-*d*_6_), δ, ppm: 44.46 (NCH_2_), 116.20, 118.88, 125.28, 129.19, 145.11 (Ar–C), 148.26 (N=CH), 150.36 (C=N), 152.89 (N=CH), 156.39 (C=N), 158.82 (Ar-C), 168.25 (C=O). Anal. Calcd. for C_14_H_13_N_7_O_2_: C, 54.02%; H, 4.21%; N, 31.50%; Found: C, 54.19%; H, 4.25%; N, 31.61%.

2-(6-Amino-9*H*-purin-9-yl)-*N*′-(pyridin-4-ylmethylene)acetohydrazide (**4m**)

Yield: 87%, m.p.: 314–316 °C, IR (νmax/cm^−1^): 3363, 3160 (NH–NH_2_), 1683 (C=O), 1593, 1575 (C=N). ^1^H-NMR (400 MHz, DMSO-*d*_6_), δ, ppm: 12.15 and 12.05 (1H, s, NH), 8.63 (2H, d, *J* = 4.0 Hz, ArH), 8.22 + 8.09 (2H, s, N=CH, *E*/*Z* amide conformer, 75/25 ratio and ArH), 8.03 (1H, s, ArH), 7.70 (2H, d, *J* = 8.0 Hz, ArH), 7.22 (2H, s, NH_2_), 5.43 + 5.00 (2H, s, NCH_2_, *cis/trans* amide conformers, 75/25 ratio). ^13^C-NMR (100 MHz, DMSO-*d*_6_), δ, ppm: 44.06 (NCH_2_), 118.49, 121.62, 141.05, 142.07 (Ar–C), 150.21 (N=CH), 150.91 (C=N), 152.72 (N=CH), 156.25 (C=N), 169.25 (C=O). Anal. Calcd. for C_13_H_12_N_8_O: C, 52.70%; H, 4.08%; N, 37.82%; Found: C, 52.88%; H, 4.12%; N, 37.93%.

2-(6-Amino-9*H*-purin-9-yl)-*N*′-(furan-2-ylmethylene)acetohydrazide (**4n**)

Yield: 85%, m.p.: 288–290 °C, IR (νmax/cm^−1^): 3336, 3277 (NH–NH_2_), 1687 (C=O), 1661, 1607, 1575 (C=N). ^1^H-NMR (400 MHz, DMSO-*d*_6_), δ, ppm: 11.80 and 11.71 (1H, s, NH), 8.09 (2H, s, N=CH, *E*/*Z* amide conformer and ArH), 7.93 (1H, s, ArH), 7.83 (1H, s, ArH), 7.19 (2H, s, NH_2_), 6.93 (1H, s, ArH), 6.62 (1H, s, ArH), 5.30 + 4.94 (2H, s, NCH_2_, *cis/trans* amide conformers, 75/25 ratio). ^13^C APT (100 MHz, DMSO-*d*_6_), δ, ppm: 44.06 (NCH_2_), 112.45, 114.56, 118.80, 135.01 (Ar–C), 149.20 (N=CH), 150.21 (C=N), 153.04 (N=CH), 156.56 (C=N), 168.23 (C=O). Anal. Calcd. for C_12_H_11_N_7_O_2_: C, 50.53%; H, 3.89%; N, 34.37%; Found: C, 50.68%; H, 3.92%; N, 34.41%.

2-(6-Amino-9*H*-purin-9-yl)-*N*′-((benzo[*d*][1,3]dioxol-5-yl)methylene)acetohydrazide (**4o**)

Yield: 84%, m.p.: 276–278 °C, IR (νmax/cm^−1^): 3325, 3200 (NH–NH_2_), 1668 (C=O), 1638, 1598 (C=N). ^1^H-NMR (400 MHz, DMSO-*d*_6_), δ, ppm: 11.81 and 11.70 (1H, s, NH), 8.12 (2H, d, ArH and N=CH, *E*/*Z* amide conformer, 75/25 ratio), 7.87 (1H, s, ArH), 7.41 (1H, s, ArH), 7.25 (2H, s, NH_2_), 7.18 (1H, d, *J* = 8.0 Hz, ArH), 7.00 (1H, d, *J* = 8.0 Hz, ArH), 6.10 (2H, s, OCH_2_O), 5.40 + 4.97 (2H, s, NCH_2_, *cis/trans* amide conformers, 80/20 ratio). ^13^C-NMR (100 MHz, DMSO-*d*_6_), δ, ppm: 44.10 (NCH_2_), 102.06 (OCH_2_O), 105.45, 108.83, 119.00, 123.77, 128.79, 144.46, 148.46 (Ar–C), 149.51 (N=CH), 150.47 (C=N), 152.88 (N=CH), 156.28 (C=N), 168.85 (C=O). Anal. Calcd. for C_15_H_13_N_7_O_3_: C, 53.10%; H, 3.86%; N, 28.90%; Found: C, 53.25%; H, 3.90%; N, 29.00%.

2-(6-Amino-9*H*-purin-9-yl)-*N*′-(4-(4-phenylpiperazin-1-yl)benzylidene)acetohydrazide (**4p**)

Yield: 82%, m.p.: 286–287 °C, IR (νmax/cm^−1^): 3325, 3140 (NH–NH_2_), 1672 (C=O), 1657, 1599, 1588 (C=N). ^1^H-NMR (400 MHz, DMSO-*d*_6_), δ, ppm: 11.64 and 11.56 (1H, s, NH), 8.10 (2H, s, ArH), 7.95 (1H, s, N=CH, *E*/*Z* amide conformer), 7.59 (2H, d, *J* = 8.0 Hz, ArH), 7.20 (4H, s, ArH and NH_2_), 7.05–6.97 (4H, m, ArH), 6.79 (1H, t, *J* = 12.0 Hz, ArH), 5.37 + 4.94 (2H, s, NCH_2_, *cis/trans* amide conformers, 80/20 ratio), 3.36 (4H, s, NCH_2_), 3.26 (4H, s, NCH_2_). ^13^C APT (100 MHz, DMSO-*d*_6_), δ, ppm: 44.47 (NCH_2_), 47.56 (NCH_2_), 48.99 (NCH_2_), 115.19, 115.94, 118.51, 119.82, 124.44, 128.79, 129.82 (Ar–C), 145.31 (N=CH), 150.44 (C=N), 151.18, 152.49 (Ar-C), 153.02 (N=CH), 156.33 (C=N), 168.15 (C=O). Anal. Calcd. for C_24_H_25_N_9_O: C, 63.28%; H, 5.53%; N, 27.67%; Found: C, 63.45%; H, 5.58%; N, 27.75%.

### 3.2. Cell Viability Assay (MTT Assay)

Human lung adenocarcinoma A549 cells (ATCC^®^ CCL-185™) and normal human lung fibroblast MRC-5 cells (ATCC^®^ CCL-171™) were obtained from the American Type Culture Collection (Manassas, VA, USA). Cells were propagated in Dulbecco’s modified Eagle’s medium (DMEM) containing 10% fetal bovine serum, 2 mM L-glutamine, 100 U/mL penicillin, and 100 mg/L streptomycin (Biochrom AG, Berlin, Germany) under humidified conditions at 37 °C and 5% CO_2_. Antiproliferative activity was examined by the MTT assay. Cells were dispensed into 96-well plates at an initial density of 1 × 10^5^ cells/mL and exposed for 48 h to the synthesized derivatives at 0.5, 5, 10, 25, 50, 100, or 250 µM. Compound stock solutions were prepared in DMSO and diluted with culture medium immediately before use. The final solvent concentration was maintained below 0.1% (*v*/*v*) in all treatment groups, and an equivalent DMSO concentration was included in vehicle-control wells. No measurable effect of the vehicle on cell viability was observed. Since acylhydrazones with extended aromatic conjugations exhibit high thermodynamic stability against hydrolysis under physiological conditions (pH 7.4), stock solutions diluted in cell culture medium (pH 7.4, ≤0.1% DMSO) demonstrated complete chemical stability during a 48-h biological testing incubation period.

At the end of exposure, the resulting formazan product was solubilized in DMSO and absorbance was recorded at 570 nm using a BMG Labtech microplate reader (Ortenberg, Germany). Doxorubicin at 0.5, 5, and 50 µM served as the cytotoxic positive control. Concentration–response data obtained from three independent experiments were fitted to a four-parameter logistic model using GraphPad Prism 9.5.1 (San Diego, CA, USA), and IC_50_ values were estimated with 95% confidence intervals.

### 3.3. LDH Leakage Assay

Membrane integrity was evaluated by quantifying extracellular lactate dehydrogenase activity using a commercial LDH assay kit (Cat. No. ab102526, Abcam, Cambridge, UK). A549 cells were cultured in 96-well plates at 1 × 10^5^ cells/well and exposed for 48 h to the IC_50_ concentration of each selected compound. At the end of treatment, 100 µL of culture supernatant was collected from each well and mixed with an equal volume of the kit reaction mixture in a separate 96-well plate. Following incubation for 30 min at room temperature in the dark, absorbance was recorded at 490 nm using a BMG Labtech microplate reader (Ortenberg, Germany).

### 3.4. Total Oxidant Status (TOS) Levels

Cellular oxidant burden was determined with a Total Oxidant Status assay kit (Rel Assay Diagnostics^®^, Gaziantep, Türkiye). A549 cells were treated for 48 h with the respective IC_50_ concentrations of the selected derivatives, after which samples were processed according to the kit procedure. The assay is based on oxidation of a ferrous ion–chelator complex to the ferric state by oxidizing species present in the sample. Hydrogen peroxide was used for calibration, and TOS values were reported as µmol H_2_O_2_ equivalents/L. Three independent biological experiments were performed for each condition.

### 3.5. Determination of Intracellular GSH/GSSG Ratio

Intracellular redox status was assessed using the GSH/GSSG Ratio Detection Assay Kit (Cat. No. 703002, Cayman Chemical, Ann Arbor, MI, USA). A549 cells were seeded in 6-well plates at 1 × 10^5^ cells/well and exposed to the IC_50_ concentrations of the selected compounds for 48 h. Cells were then rinsed with ice-cold PBS and lysed in the MES-based buffer supplied with the kit. To minimize artificial oxidation during sample preparation, lysates were immediately deproteinized with metaphosphoric acid and subsequently neutralized. Total glutathione and GSSG were quantified separately using the DTNB/glutathione reductase recycling system, with absorbance measured at 405 nm. Reduced GSH was calculated from total glutathione after correction for twice the measured GSSG concentration, and the resulting GSH/GSSG ratio was used as an indicator of intracellular redox balance.

### 3.6. Intracellular Reactive Oxygen Species (iROS)

Intracellular oxidant generation was quantified by DCFH-DA fluorescence. A549 cells were plated in 6-well plates at 1 × 10^5^ cells/well, allowed to attach for 24 h, and then exposed to the IC_50_ concentration of each selected compound for 48 h. Following treatment, the monolayers were rinsed with warm PBS and loaded with 10 µM DCFH-DA in serum-free medium for 30 min at 37 °C under light-protected conditions. Cells were subsequently washed, detached using an enzyme-free dissociation buffer, and resuspended in ice-cold PBS for immediate flow-cytometric acquisition. Fluorescence was recorded using 488 nm excitation and a 530/30 nm emission filter. After exclusion of debris and non-singlet events, at least 10,000 cells were analyzed per sample. ROS production was expressed relative to the untreated control based on median fluorescence intensity. Three independent biological replicates were evaluated.

### 3.7. Measurement of Mitochondrial Membrane Potential (MMP, ΔΨm)

Mitochondrial membrane potential was examined using JC-1 dye (Cat. No. HY-K0601, MedChemExpress, Monmouth Junction, NJ, USA). A549 cells were seeded in 96-well plates at 1 × 10^5^ cells/well and exposed to the IC_50_ concentrations of the selected derivatives for 48 h. Following treatment, JC-1 staining solution was applied for 20 min at 37 °C in the dark. Cells were then washed with the assay buffer supplied with the kit. Fluorescence was recorded, and mitochondrial polarization was evaluated from the ratio of red JC-1 aggregate fluorescence to green JC-1 monomer fluorescence. Measurements were obtained from three independent biological experiments.

### 3.8. Cell Cycle Analysis

Changes in cell cycle distribution were determined by propidium iodide staining followed by flow cytometry. A549 cells were plated in 6-well plates at 1 × 10^5^ cells/well and left overnight for attachment before being exposed for 48 h to the IC_50_ concentrations of the selected derivatives. Both adherent and non-adherent cells were recovered after treatment to avoid loss of damaged cell populations. Cells were washed twice with cold PBS (Gibco, Thermo Fisher Scientific, Waltham, MA, USA) and fixed overnight at 4 °C in 70% ice-cold ethanol. After fixation, the samples were centrifuged and resuspended in 500 µL of PI/RNase staining solution containing 50 µg/mL propidium iodide and 100 µg/mL RNase A (Sigma-Aldrich, St. Louis, MO, USA). Staining was allowed to proceed for 30 min at 37 °C in the dark. Samples were analyzed using a Muse™ Cell Analyzer (Merck Millipore, Darmstadt, Germany), with a minimum of 10,000 events acquired for each condition. DNA-content histograms were processed using the Muse™ Cell Cycle Analysis software module (version 1.4), and cells were assigned to the Sub-G1, G0/G1, S, and G2/M populations. Three independent biological experiments were performed, and results were summarized as mean ± SD.

### 3.9. Apoptosis Analysis (Annexin V–FITC/PI Flow Cytometry)

Apoptosis-associated changes were examined with the Muse™ Annexin V & Dead Cell Kit (Cat. No. MCH100105, Merck Millipore, Burlington, MA, USA). A549 cells were seeded into 6-well plates at 4 × 10^5^ cells/well and allowed to attach overnight. The selected compounds were subsequently applied at their respective IC_50_ concentrations for 48 h. Following exposure, floating cells and adherent cells were pooled, centrifuged at 1000× *g* for 10 min at 4 °C, and washed with PBS. Cell pellets were resuspended in culture medium, and a 100 µL aliquot of each suspension was combined with an equal volume of Annexin V reagent. Samples were protected from light and incubated for 20 min at room temperature before acquisition on a Muse™ Cell Analyzer. Debris and doublets were excluded using FSC/SSC and FSC-A/FSC-H gating, respectively, and a minimum of 10,000 events were collected per sample. Cells were categorized as viable (Annexin V^−^/PI^−^), early apoptotic (Annexin V^+^/PI^−^), late apoptotic/dead (Annexin V^+^/PI^+^), or necrotic (Annexin V^−^/PI^+^). Data represent the mean ± SD of three independent biological experiments.

### 3.10. Statistical Analysis

Statistical analyses were conducted using SPSS software (version 22.0; SPSS Inc., Chicago, IL, USA). Intergroup variations were evaluated through one-way analysis of variance (ANOVA), and subsequent multiple comparisons were performed using Tukey’s post hoc test. To ensure reproducibility, all experiments were carried out in triplicate using distinct, independently prepared cell cultures on separate days. Data points are presented as the mean ± standard deviation (SD) of three independent biological replicates. Statistical significance was defined as a *p*-value of 0.05 or less (*p* ≤ 0.05).

### 3.11. Computational Methods

#### 3.11.1. Molecular Docking Protocol

All computational modeling and molecular docking simulations were conducted using the Schrödinger software suite (Release 2018-4, Schrödinger, LLC, New York, NY, USA). The Maestro graphical user interface was utilized for the visualization and management of the computational workflows. The high-resolution X-ray crystallographic structures of the target proteins, Polo-like kinase 1 (PLK1, PDB ID: 2RKU), Thioredoxin Reductase 1 (TrxR1, PDB ID: 2ZZC), and the MDM2-p53 complex (PDB ID: 4HG7), were retrieved from the RCSB Protein Data Bank (PDB) [61,62,63]. The raw PDB structures were refined and optimized using the Protein Preparation Wizard [64]. The preparation pipeline included the assignment of correct bond orders, addition of missing hydrogen atoms, and the deletion of co-crystallized solvent and water molecules located beyond 5 Å of the co-crystallized ligands. Missing side chains and loops were filled using the Prime module. The hydrogen-bonding network was optimized via PROPKA at pH 7.0. Finally, a restrained structural minimization was performed using the OPLS3 force field until the root-mean-square deviation (RMSD) of the non-hydrogen atoms converged to 0.30 Å.

The 2D chemical structures of the synthesized adenine-based derivatives were drawn and converted into 3D geometries. Prior to docking, the ligands were prepared using the LigPrep module. This process included the generation of possible tautomeric and stereoisomeric states, with protonation states assigned by Epik at a physiological pH of 7.4 ± 2.0. The generated ligand structures were subsequently energy-minimized using the OPLS3 force field to yield the lowest-energy conformations. To accurately mimic the physiological binding conditions and account for the conformational flexibility of the receptor active sites upon ligand entry, the Induced Fit Docking (IFD) protocol was employed [65]. This advanced methodology integrates Glide docking with Prime structural refinement to overcome the limitations of rigid-receptor docking.

The receptor grid was generated around the centroid of the native co-crystallized ligands for each target. The standard IFD workflow was executed in four sequential stages: (1) initial softened-potential docking of the prepared ligands into the binding pocket using Glide, where the van der Waals radii of non-polar atoms for both the receptor and ligands were scaled down by a factor of 0.5 to allow for initial steric clashes; (2) prime side-chain prediction and structural refinement for all receptor residues located within a 5.0 Å radius of the docked ligand poses; (3) prime energy minimization of the newly generated ligand-receptor complexes; and (4) re-docking of the ligands into the refined, induced-fit receptor conformations utilizing the Glide Extra Precision (XP) scoring function [66]. The final ligand-receptor complexes were ranked based on their Glide XP docking scores (kcal/mol).

#### 3.11.2. Protocol Validation

To validate the reliability and reproducibility of our docking protocol, we performed redocking of the co-crystallized native ligands back into the binding pockets of their respective protein structures. The calculated Heavy-Atom Root-Mean-Square Deviation (RMSD) values between the re-docked poses and the original crystallographic binding geometries were 0.82 Å for PLK1, 1.05 Å for TrxR1, and 0.68 Å for MDM2. All values are significantly below the standard acceptable threshold of 2.0 Å, confirming the robustness and high accuracy of our docking setup.

#### 3.11.3. Molecular Dynamics (MD) Simulations

Time-dependent conformational stability and binding site integrity of lead compound **3d** in complex with PLK1 (PDB ID: 2RKU), TrxR1 (PDB ID: 2ZZC), and MDM2 (PDB ID: 4HG7) were investigated using the Desmond Molecular Dynamics System [67]. The initial coordinates of the protein–ligand complexes were obtained from the top-ranked Induced Fit Docking (IFD) poses. Each complex was minimized and placed in an orthorhombic periodic boundary box with a minimum buffer distance of 10 Å using the OPLS4 force field. The solvation environment was constructed using explicit TIP3P water molecules, and the net system charge was neutralized by adding ions to achieve a physiological ionic strength of 0.15 M NaCl. Prior to production MD runs, all system configurations were relaxed using the standard pre-defined Desmond multi-step equilibration protocol, comprising initial energy minimization followed by short NVT and NPT ensemble equilibrations. Production simulations were performed for 100 ns under the NPT ensemble at a constant temperature of 310 K (maintained via a Nose-Hoover chain thermostat) and an isotropic pressure of 1.01325 bar (controlled using a Martyna–Tobias–Klein barostat). Integration step sizes were set to 1.2 fs, and trajectory frames were recorded every 100 ps, yielding 1000 trajectory frames per simulation run. Simulation Interaction Analysis (SID) tool in Maestro was utilized to extract RMSD, RMSF, and protein–ligand interaction fraction profiles.

#### 3.11.4. In Silico ADMET and Druglikeness Prediction

Pharmacokinetic parameters, absorption, distribution, metabolism, excretion, and toxicity (ADMET) descriptors, as well as druglikeness profiles for all synthesized adenine–hydrazone derivatives (**3a–g** and **4–18**), were calculated using the QikProp (version 5.8) module integrated within the Schrödinger Release (version 2018-4). Prior to ADMET prediction, the three-dimensional (3D) low-energy molecular structures of all derivatives were generated and energy-minimized using the LigPrep module (version 4.4) with the OPLS4 force field at a target physiological pH of 7.4 ± 0.5. All calculated parameters were evaluated against the standard reference ranges applicable to 95% of known oral drugs [68].

## 4. Conclusions

The adenine structure serves as a crucial starting material for the synthesis of potential bioactive compounds, and interest in this compound is growing steadily. Many commercially available substances-including eritadenine, zeatin, puromycin, and various antibiotics, antivirals, and anticholesteremic agents incorporating the adenine moiety into their structures. One of the primary objectives of this study was to address a gap in the literature by synthesizing adenine–hydrazone hybrid compounds and elucidating their structures. A review of the literature revealed no prior studies investigating the activity of such adenine-derived compounds against lung cancer. Furthermore, electron-donating and electron-withdrawing groups were incorporated into the aromatic structure; a key aim of the study was to determine how these groups influence biological activity. Another objective was to elucidate the structure–activity relationship through molecular docking studies.

In this study, a new series of adenine-based hydrazone derivatives were successfully designed, synthesized, and evaluated for their antiproliferative activity in human lung adenocarcinoma (A549) cells and their effects on normal lung fibroblast (MRC-5) cells. Among the tested compounds, **3d**, **3c**, and **4j** exhibited the strongest antiproliferative activity within the series against A549 cells, with IC_50_ values of 37.2 ± 1.8 µM, 41.8 ± 1.9 µM, and 42.9 ± 1.7 µM, respectively, while showing comparatively greater preservation of MRC-5 cell viability than several other derivatives in the series. Mechanistic studies demonstrated that the selected compounds induced oxidative stress-associated cellular injury, characterized by increased intracellular ROS generation, elevated total oxidant status, and disruption of the GSH/GSSG redox balance. These changes were accompanied by mitochondrial membrane depolarization, G2/M phase accumulation, and Annexin V-confirmed apoptotic cell death. Molecular docking analyses provided a complementary structural framework for the experimentally observed cellular responses, revealing favorable predicted interactions of the lead compounds with PLK1, TrxR1, and MDM2. These in silico findings support the biological plausibility of pathways related to cell cycle regulation, redox homeostasis, and apoptosis, while direct target-specific validation will be required to establish the precise contribution of these proteins to the observed effects. Structure–activity relationship analysis indicated that halogen substitution, particularly fluoro and chloro substitution at the 5-position of the isatin ring, was associated with improved antiproliferative activity and a more favorable A549/MRC-5 profile within the series. Overall, these findings support adenine–hydrazone derivatives as a structurally promising platform for further optimization and mechanistic investigation in the A549 lung adenocarcinoma model, while validation across additional lung cancer models will be required before broader anticancer or therapeutic conclusions can be drawn.

## Figures and Tables

**Table 1:** IC_50_ (µM) values of the novel synthesized adenine–hydrazone compounds and positive control (Doxorubicin) on A549 lung adenocarcinoma cells and MRC-5 normal lung fibroblast cells after 48 h of incubation.

Compound	A549 IC_50_ (µM)	MRC-5 IC_50_ (µM)
**3a**	95.7 ± 3.1	91.3 ± 2.9
**3b**	82.4 ± 2.8	76.8 ± 2.6
**3c**	41.8 ± 1.9	80.4 ± 2.8
**3d**	37.2 ± 1.8	73.3 ± 2.2
**3e**	88.5 ± 2.9	73.6 ± 2.5
**3f**	59.4 ± 2.2	47.6 ± 2.1
**3g**	126.8 ± 3.8	129.7 ± 3.9
**4a**	96.4 ± 2.7	98.8 ± 2.9
**4b**	86.9 ± 2.8	79.6 ± 2.7
**4c**	79.8 ± 2.5	74.5 ± 2.4
**4d**	83.9 ± 2.4	61.5 ± 2.1
**4e**	108.6 ± 3.6	114.9 ± 3.7
**4f**	118.9 ± 3.5	52.7 ± 2.0
**4g**	98.5 ± 2.8	93.6 ± 2.7
**4h**	93.8 ± 2.9	81.7 ± 2.5
**4i**	104.7 ± 3.1	101.6 ± 3.0
**4j**	42.9 ± 1.7	63.8 ± 2.1
**4k**	78.6 ± 2.5	71.2 ± 2.4
**4l**	94.8 ± 3.1	101.2 ± 3.3
**4m**	74.2 ± 2.4	46.8 ± 1.9
**4n**	92.7 ± 2.9	86.1 ± 2.5
**4o**	116.8 ± 3.4	109.5 ± 3.2
**4p**	72.8 ± 2.6	39.4 ± 1.8
Doxorubicin *	1.84 ± 0.15	5.24 ± 0.42

* Clinical reference positive control. IC_50_ values represent the mean ± standard deviation (SD) of three independent biological experiments (n = 3).

**Table 2:** Effects of selected adenine–hydrazone derivatives on LDH release, TOS levels, and intracellular GSH/GSSG ratio in A549 cells.

Treatment (48 H, at Ic_50_)	LDH (U/L)	TOS (µM H_2_O_2_ EQ/L)	GSH/GSSG
Control	6.4 ± 0.5	14.8 ± 1.1	8.7 ± 0.6
**H_2_O_2_ (250 µM)** *	26.8 ± 1.9 ***	39.4 ± 2.3 ***	3.1 ± 0.2 ***
**3** **c**	12.6 ± 0.8 **	22.0 ± 1.5 **	6.2 ± 0.4 **
**3** **d**	15.9 ± 1.0 ***	27.6 ± 1.9 ***	4.9 ± 0.4 ***
**4** **j**	13.8 ± 0.9 **	24.3 ± 1.7 **	5.5 ± 0.4 **

Values are expressed as mean ± SD (n = 3). * *p* < 0.05, ** *p* < 0.01, *** *p* < 0.001 compared to the untreated control. Hydrogen peroxide (H_2_O_2_, 250 µM) was utilized as a positive control for oxidative stress induction.

**Table 3:** The molecular docking scores (kcal/mol) of the synthesized adenine-based derivatives against Polo-like kinase 1 (PLK1), Thioredoxin Reductase 1 (TrxR1), and MDM2 target proteins.

Compound	PLK1 (2RKU)	TrxR1 (2ZZC)	MDM2 (4HG7)
**3a**	−9.91	−7.77	−5.96
**3b**	−9.10	−8.70	−10.83
**3c**	−11.66	−8.69	−11.37
**3d**	−11.67	−9.39	−12.19
**3e**	−11.30	−10.84	−8.50
**3f**	−13.20	−9.00	−9.73
**3g**	−6.48	−4.88	−4.88
**4a**	−8.10	−5.92	−8.04
**4b**	−9.16	−8.52	−10.83
**4c**	−9.96	−9.20	−10.83
**4d**	−10.53	−7.66	−9.72
**4e**	−6.59	−6.32	−6.47
**4f**	−5.78	−5.05	−6.34
**4g**	−8.07	−6.25	−7.68
**4h**	−9.94	−8.85	−9.57
**4i**	−6.90	−5.74	−6.57
**4j**	−11.78	−10.81	−11.05
**4k**	−10.50	−9.62	−10.49
**4l**	−10.44	−7.76	−8.71
**4m**	−10.46	−7.84	−11.32
**4n**	−9.85	−8.37	−9.74
**4o**	−6.66	−4.48	−5.73
**4p**	−11.16	−8.86	−11.02

**Table 4:** Predicted in silico ADMET properties and druglikeness parameters.

Compound	logPo/w	logS	logHERG	PCaco	logBB	PMDCK	logKhsa	PHO (%)	RoF
**3a**	0.84	−4.15	−5.67	48.77	−2.09	18.90	−0.31	62.10	0
**3b**	1.38	−5.64	−5.63	48.88	−1.97	50.12	−0.20	65.28	0
**3c**	1.31	−4.79	−5.60	48.87	−1.97	46.60	−0.22	64.86	0
**3d**	1.07	−4.48	−5.55	48.87	−2.00	34.12	−0.28	63.44	0
**3e**	1.10	−4.41	−5.38	52.28	−2.07	20.37	−0.20	64.14	0
**3f**	0.13	−4.64	−5.40	6.38	−3.08	2.10	−0.36	29.14	1
**3g**	0.87	−4.44	−5.31	52.28	−2.10	20.37	−0.32	49.86	1
**4a**	1.40	−4.18	−4.12	165.12	−1.20	148.53	−0.29	74.86	0
**4b**	1.25	−3.80	−4.01	172.46	−1.31	74.01	−0.26	74.32	0
**4c**	1.77	−4.83	−3.98	172.64	−1.06	305.38	−0.22	77.37	0
**4d**	1.75	−4.83	−3.93	199.61	−1.01	317.30	−0.23	78.35	0
**4e**	1.48	−4.18	−4.49	163.62	−1.25	162.89	−0.29	75.26	0
**4f**	1.28	−3.80	−4.41	171.39	−1.38	73.52	−0.27	74.45	0
**4g**	0.45	−4.04	−4.39	29.00	−2.13	10.77	−0.41	42.77	1
**4h**	0.38	−3.54	−4.44	53.08	−1.90	20.71	−0.48	60.02	0
**4i**	0.63	−3.84	−4.36	68.53	−1.89	27.30	−0.44	63.49	0
**4j**	1.28	−3.80	−4.42	163.80	−1.41	70.00	−0.26	74.08	0
**4k**	1.56	−4.52	−4.00	172.20	−1.11	232.23	−0.27	76.08	0
**4l**	0.39	−3.54	−4.43	56.21	−1.87	22.03	−0.48	60.56	0
**4m**	1.36	−4.29	−5.15	154.24	−1.62	65.60	−0.26	74.10	0
**4n**	0.50	−3.15	−4.24	155.44	−1.37	66.15	−0.54	69.10	0
**4o**	0.71	−3.97	−4.04	167.38	−1.32	71.66	−0.45	70.92	0
Ref. Range	−2.0/6.5	−6.5/0.5	>−5.0	25–500	−3.0/1.2	25–500	−1.5/1.5	>80%	≤1

Ref. Range: Recommended parameter ranges for 95% of known oral drugs. logPo/w: predicted octanol/water partition coefficient (−2.0 to 6.5); logS: predicted aqueous solubility (−6.5 to 0.5 mol/dm^3^); logHERG: predicted IC50 for HERG K^+^ channel blockage (concern below −5.0); PCaco: predicted Caco-2 cell permeability (<25 poor, >500 high nm/s); logBB: predicted brain/blood partition coefficient (−3.0 to 1.2); PMDCK: predicted MDCK cell permeability (<25 poor, >500 high nm/s); logKhsa: predicted binding to human serum albumin (−1.5 to 1.5); PHO: predicted percent human oral absorption (>80% high, 25–80% moderate); RoF: number of Lipinski Rule of Five violations (maximum 1).

## Data Availability

The original contributions presented in this study are included in the article/Supplementary Material. Further inquiries can be directed to the corresponding author.

## Supplementary Materials

The following supporting information can be downloaded at https://www.mdpi.com/article/10.3390/ph19101652/s1. Figure S1: FT-IR, 1H-NMR and 13C-NMR spectra of newly synthesized compounds.
