# Supplementary material for: Design, Synthesis, and Evaluation of Novel Adenine-Based Hydrazones as Potential Anticancer Agents: Cytotoxicity Against A549 and MRC-5 Cell Lines with In Silico Insights
Source: Pharmaceuticals (Basel). 2026 Oct 19;19(10):1652. doi: 10.3390/ph19101652 (PMC13708028; doi:10.3390/ph19101652)
Supplement: Supplementary file 1 [file pharmaceuticals-19-01652-s001.zip › pharmaceuticals-4571754-supplementary.pdf]

Design, Synthesis, and Evaluation of Novel Adenine-Based Hydrazones as Potential Anticancer Agents: Cytotoxicity against A549 and MRC-5 Cell Lines with In Silico Insights

Emre Menteşe [1], Nedime Çalışkan [1], Mustafa Emirik [1,*] and Adem Güner [2]

1 Department of Chemistry, Faculty of Art and Sciences, Recep Tayyip Erdogan University, 53100 Rize, Türkiye
2 Department of Occupational Health and Safety, Faculty of Health Sciences, Sinop University, 57000 Sinop, Türkiye
** Correspondence: mustafa.emirik@erdogan.edu.tr (M.E.); Tel.: +90-464-223-6126*

Figure S1. FT-IR, ^{1}H-NMR and ^{13}C-NMR spectra of newly synthesized compounds.

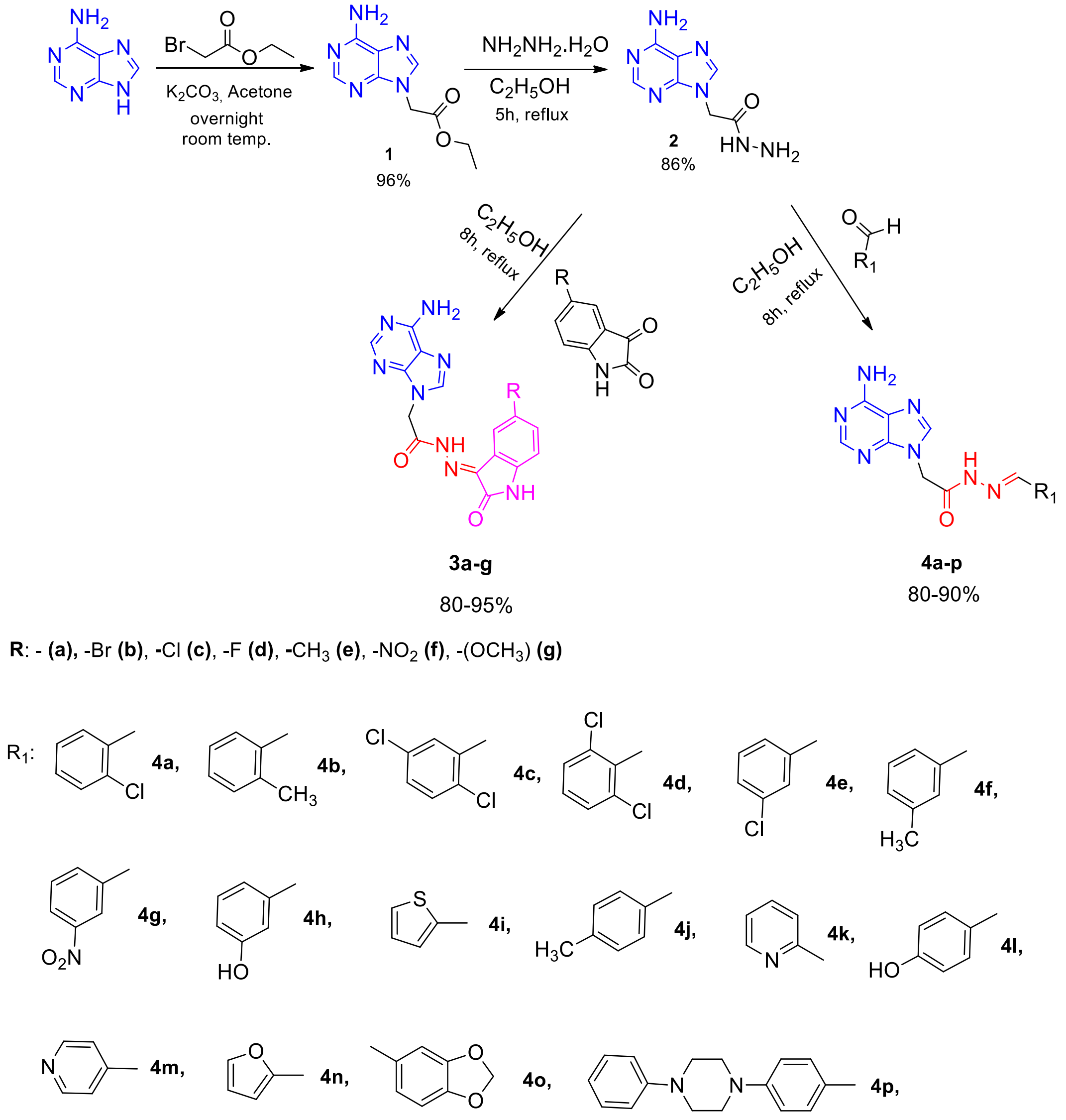

2-(6-Amino-9*H*-purin-9-yl)-*N*'-(2-oxoindolin-3-ylidene)acetohydrazide*(3a)*

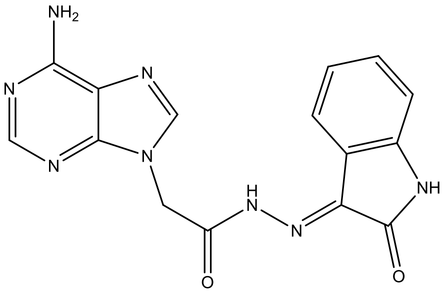

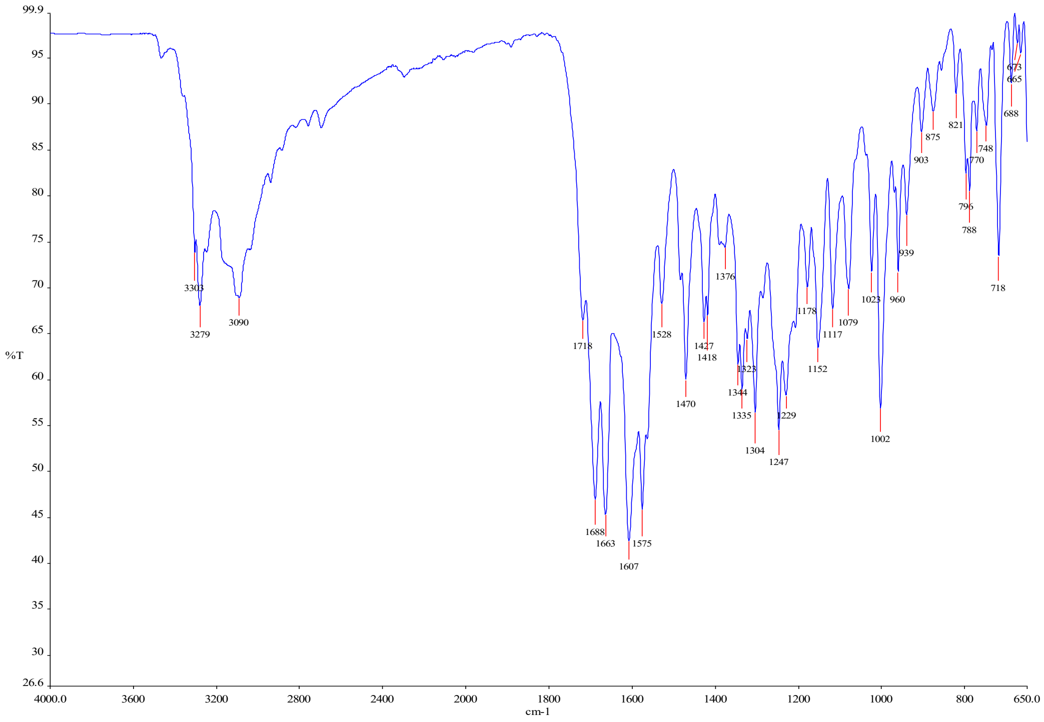

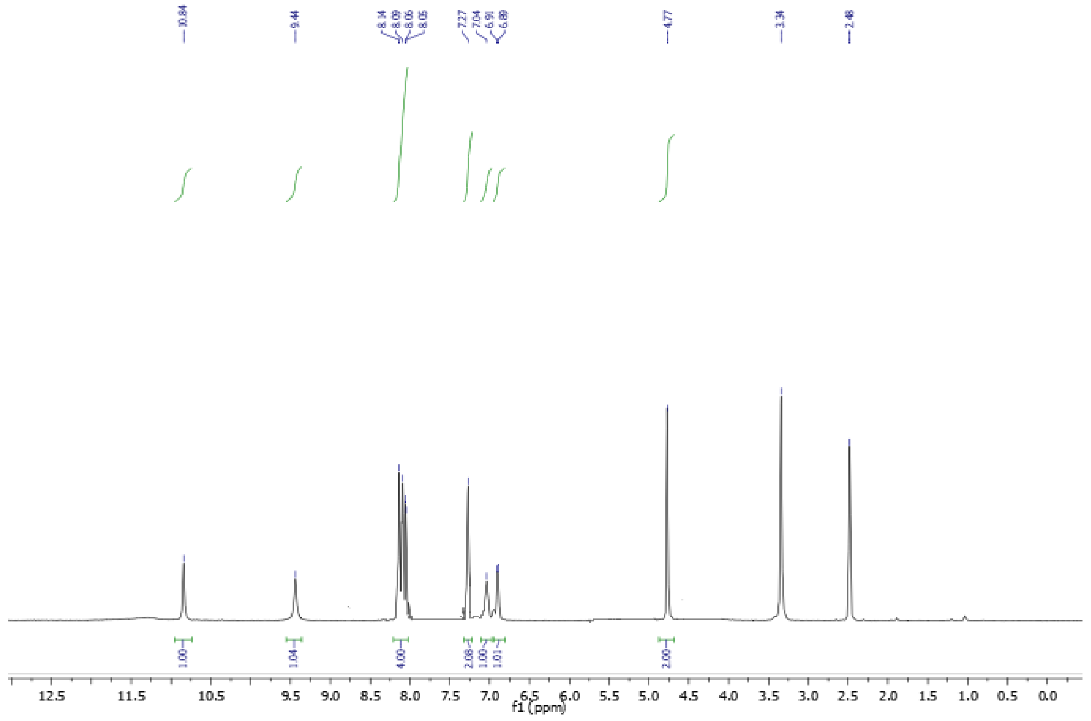
f1 (ppm)

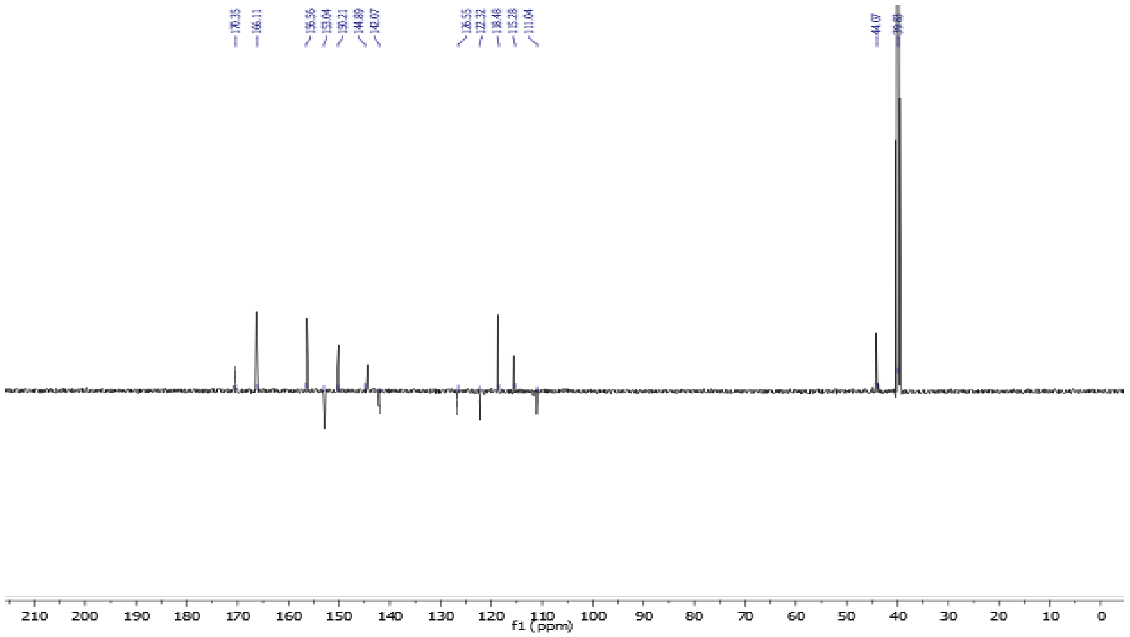
f1 (ppm)

2-(6-Amino-9*H*-purin-9-yl)-*N*'-(5-bromo-2-oxoindolin-3-ylidene)acetohydrazide(3b)

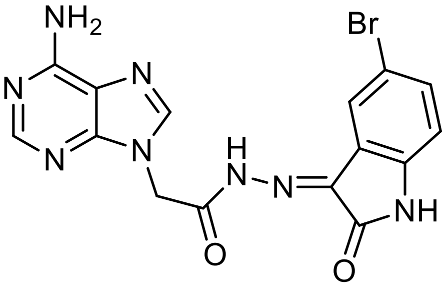

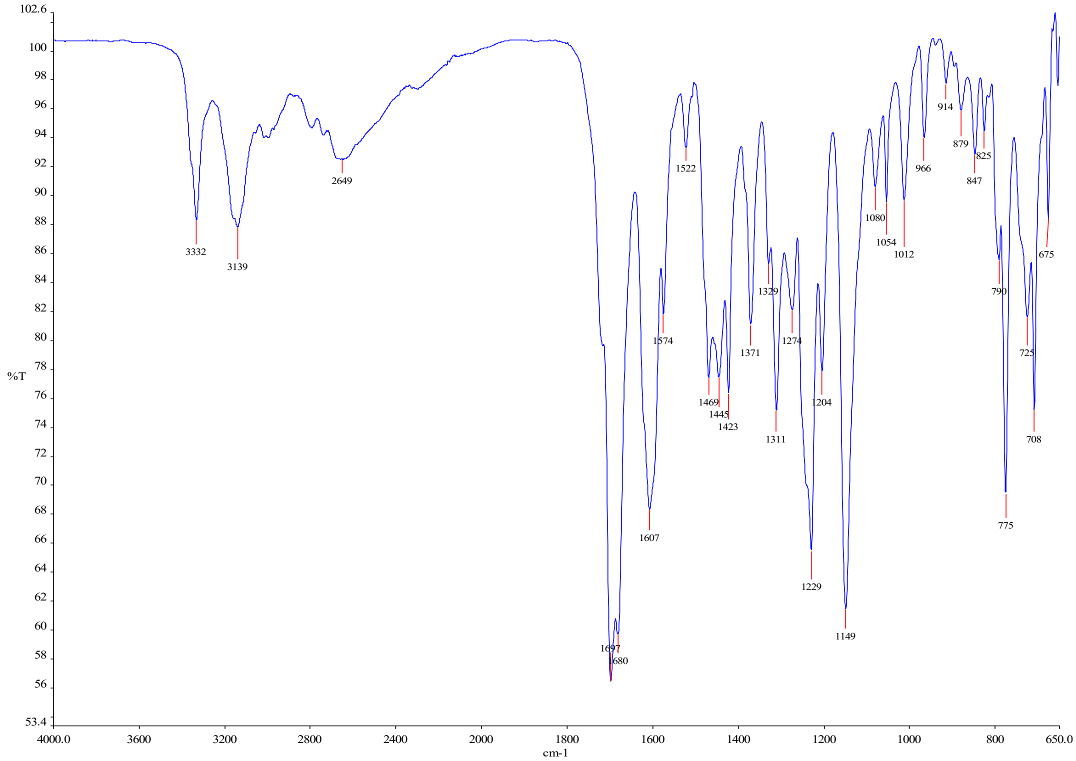

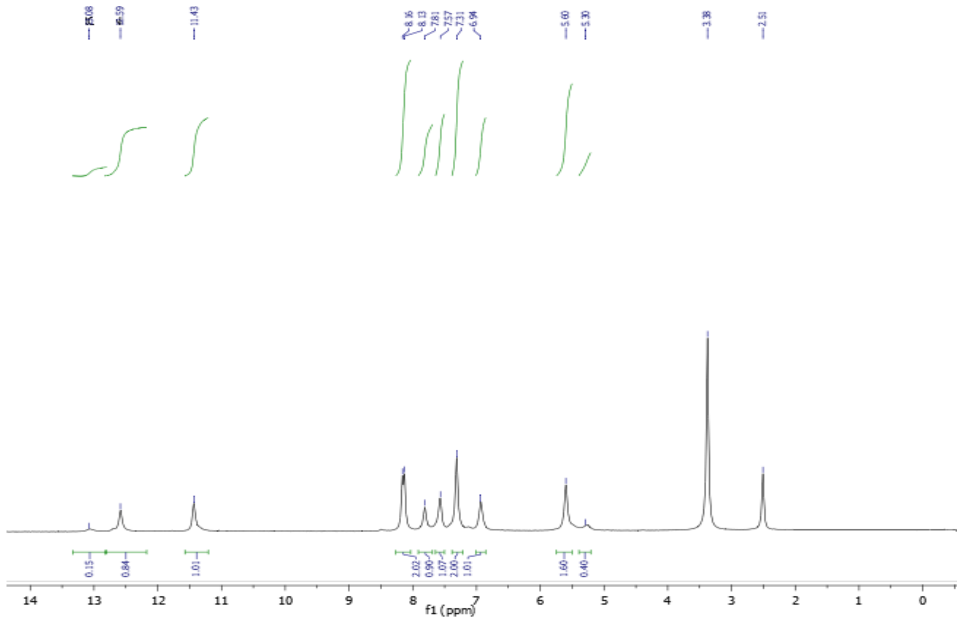

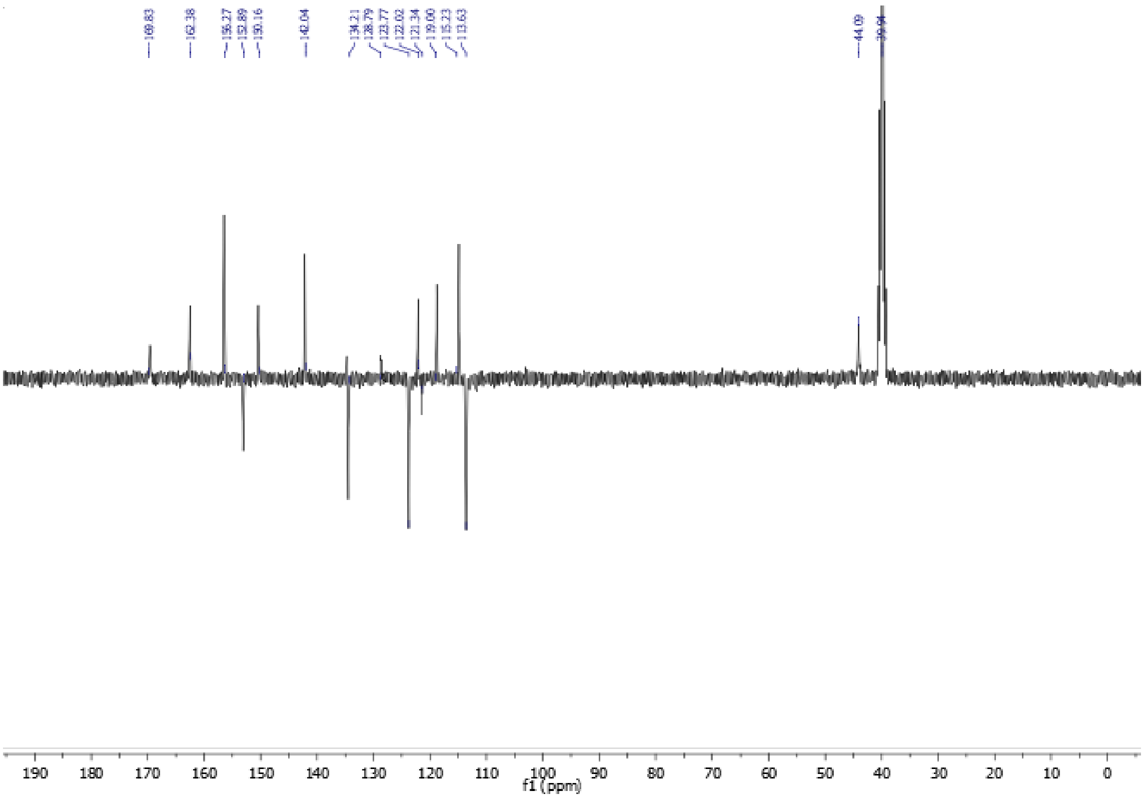
169.83
162.38
156.27
153.89
150.16
142.04
134.21
128.79
123.77
122.02
121.34
119.00
115.23
113.63
44.09
39.94
190 180 170 160 150 140 130 120 110 100 90 80 70 60 50 40 30 20 10 0
f1 (ppm)

2-(6-Amino-9*H*-purin-9-yl)-*N*'-(5-chloro-2-oxoindolin-3-ylidene)acetohydrazide(3c)

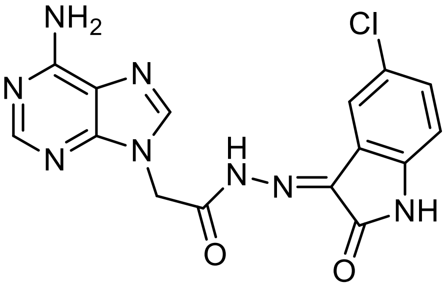

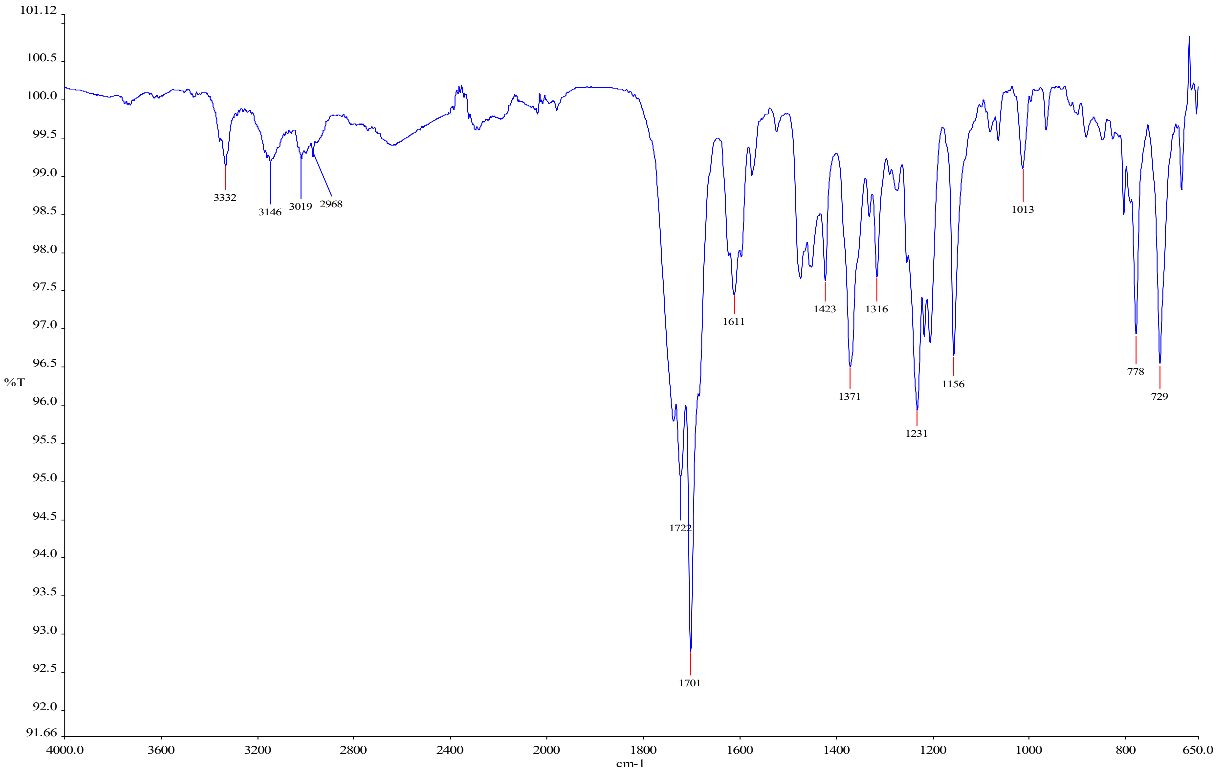

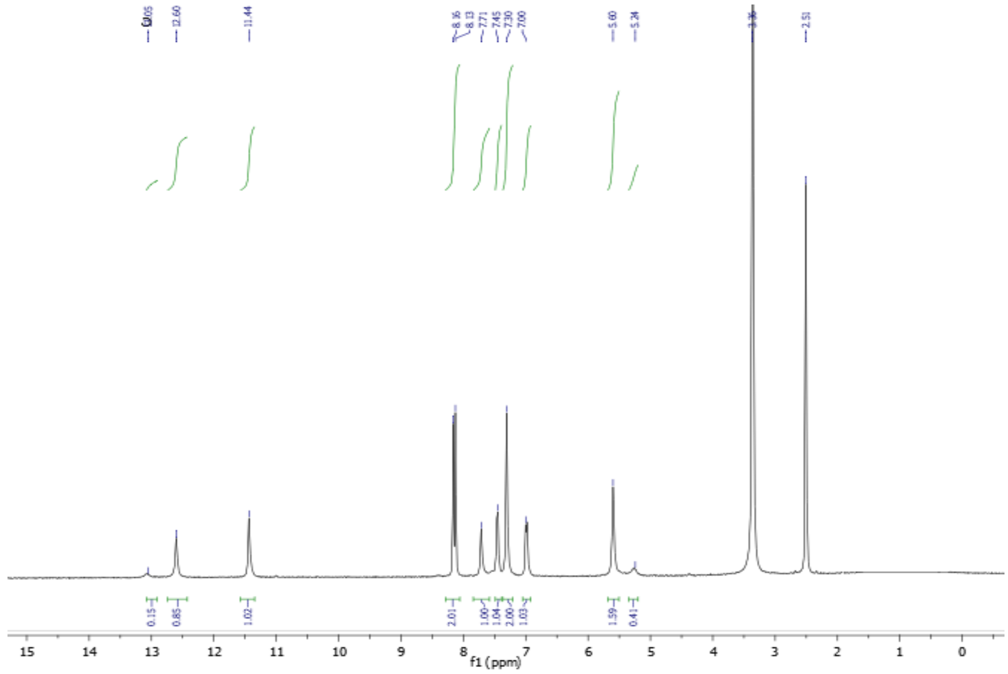
12.60
11.44
8.16
8.13
7.71
7.45
7.30
7.00
5.60
5.24
2.51
0.15
0.85
1.02
2.01
1.00
1.04
2.00
1.03
1.59
0.41
f1 (ppm)

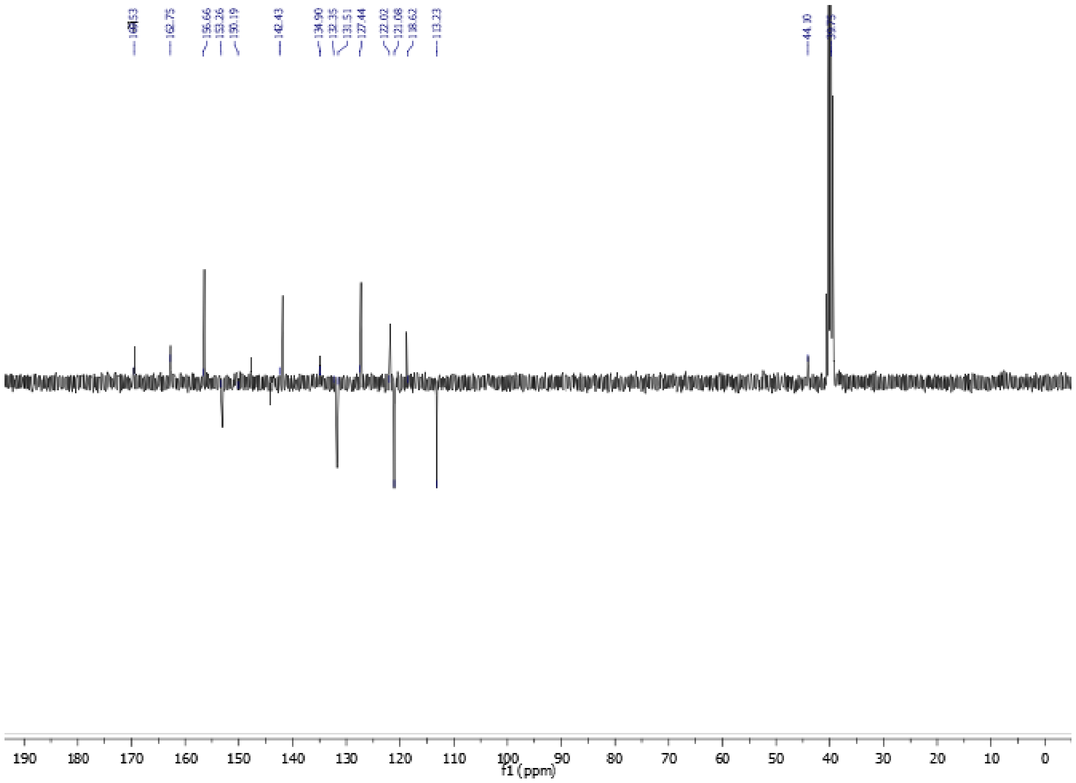
162.75
155.66
153.26
150.19
142.43
134.90
132.35
131.51
127.44
122.02
121.08
118.62
113.23
44.30
f1 (ppm)

2-(6-Amino-9*H*-purin-9-yl)-*N*'-(5-fluoro-2-oxoindolin-3-ylidene)acetohydrazide(3d)

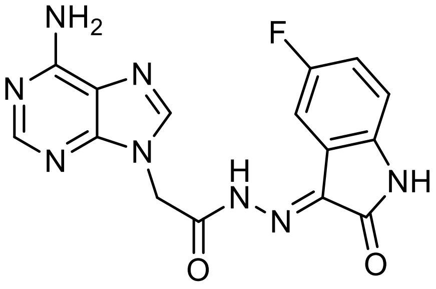

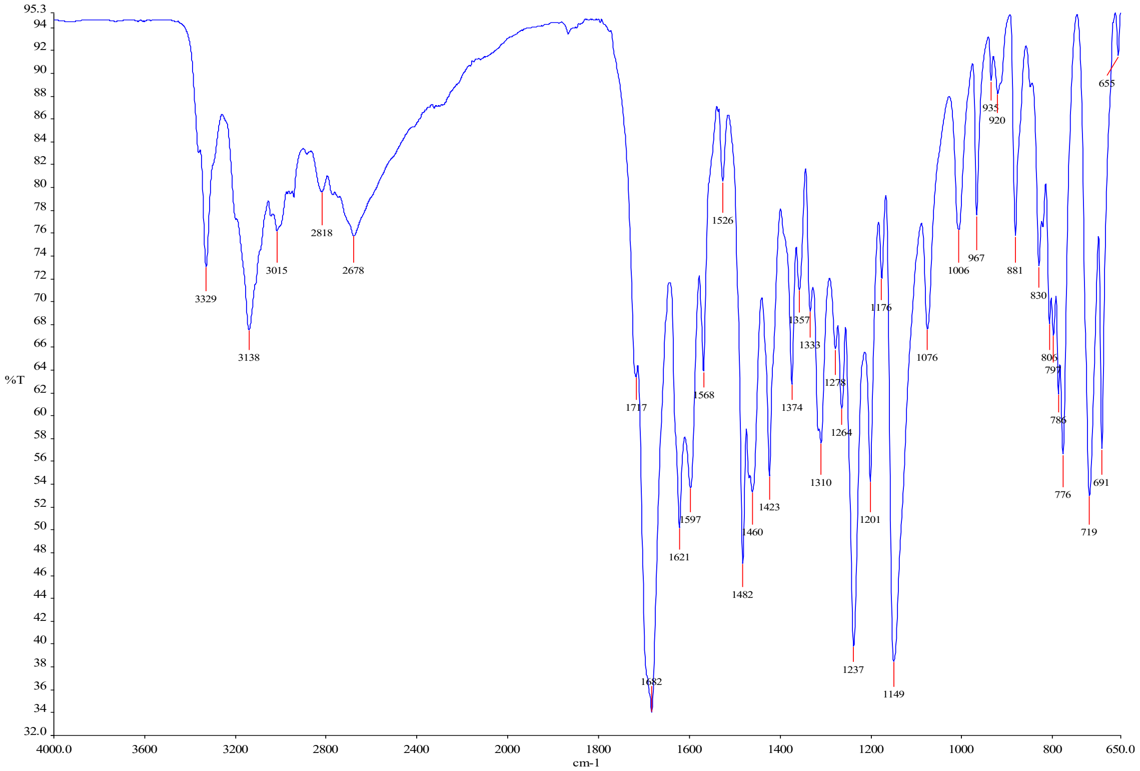

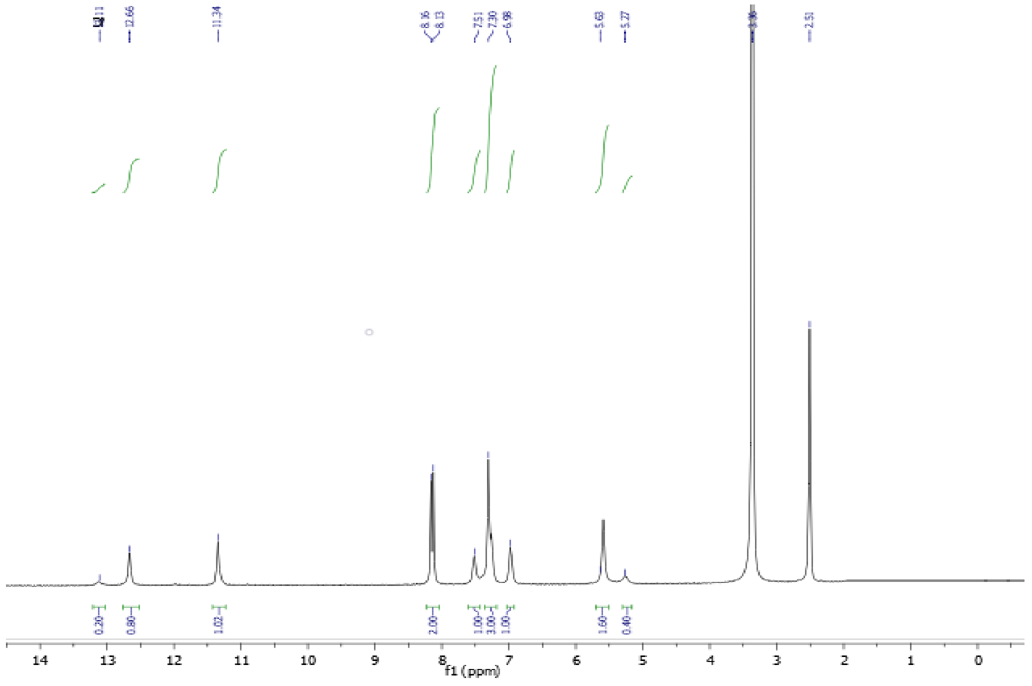

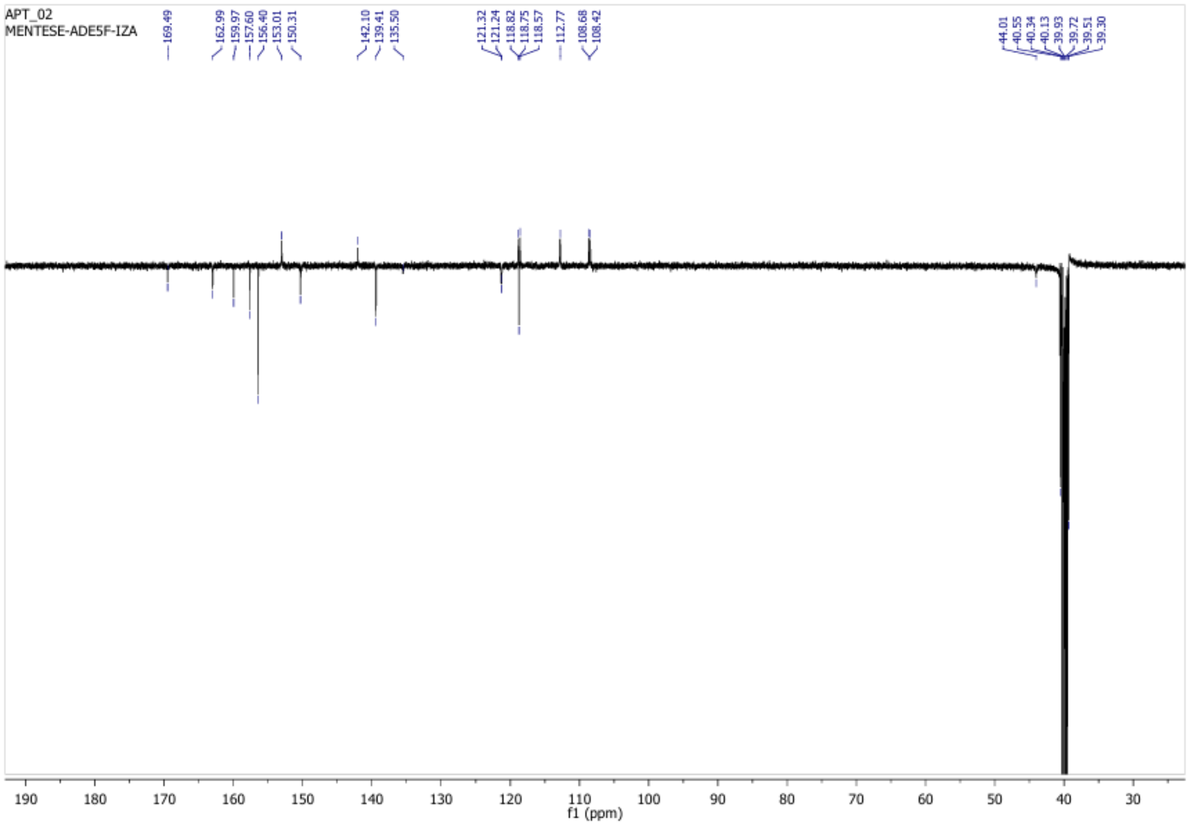
APT_02
MENTESE-ADE5F-IZA
169.49
162.99
159.97
157.60
156.40
153.01
150.31
142.10
139.41
135.50
121.32
121.24
118.82
118.75
118.57
112.77
108.68
108.42
44.01
40.55
40.34
40.13
39.93
39.72
39.51
39.30
f1 (ppm)

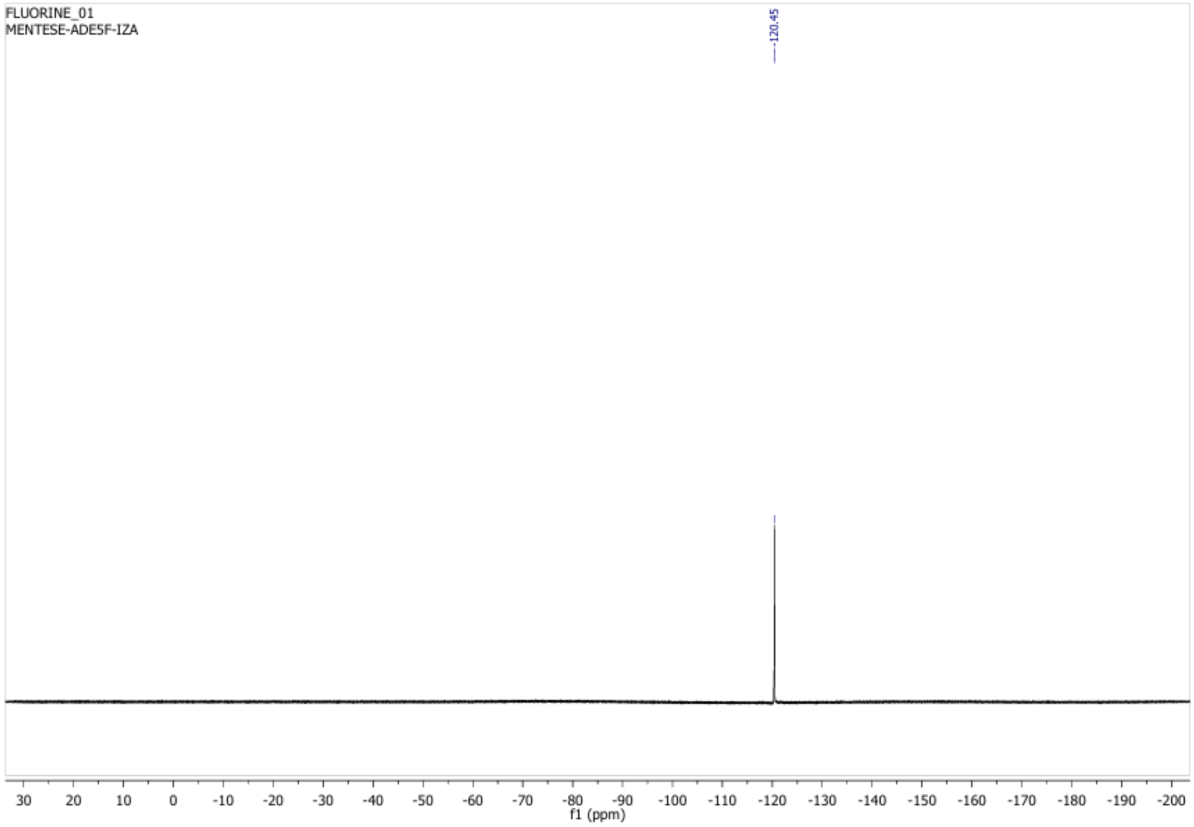
FLUORINE_01
MENTESE-ADE5F-IZA
-120.45
f1 (ppm)

2-(6-Amino-9*H*-purin-9-yl)-*N'*-(5-methyl-2-oxoindolin-3-ylidene)acetohydrazide *(3e)*

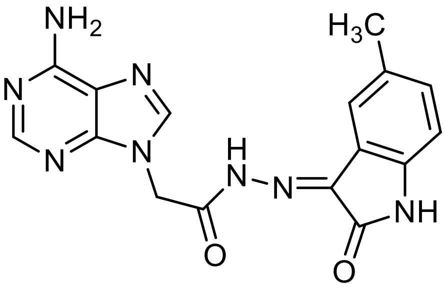

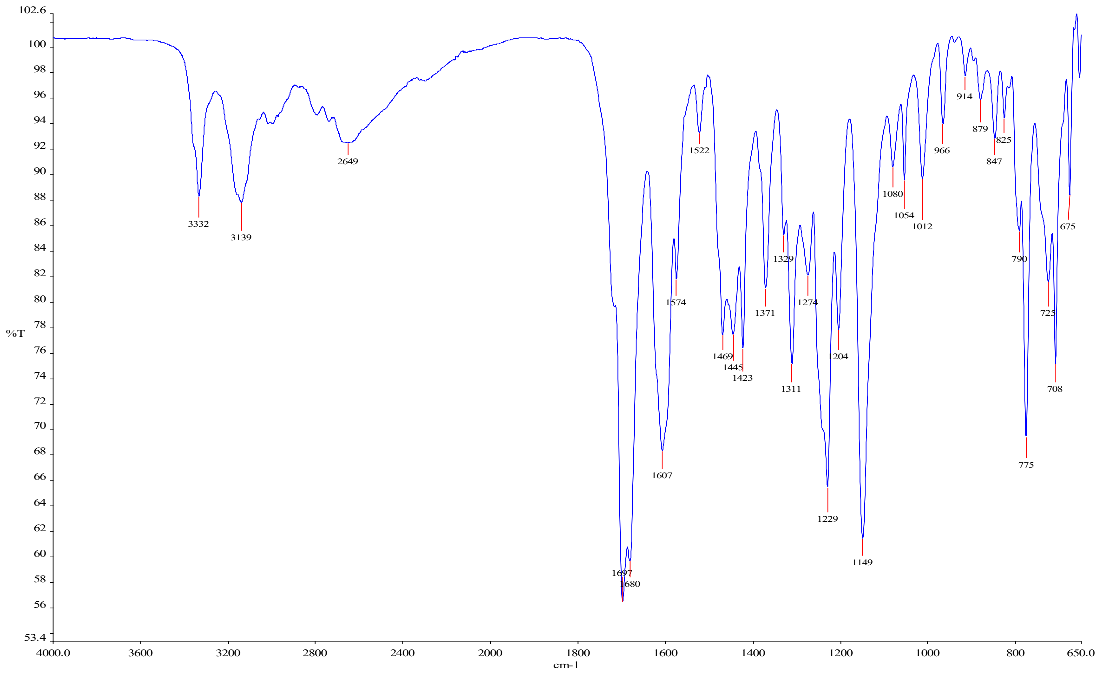

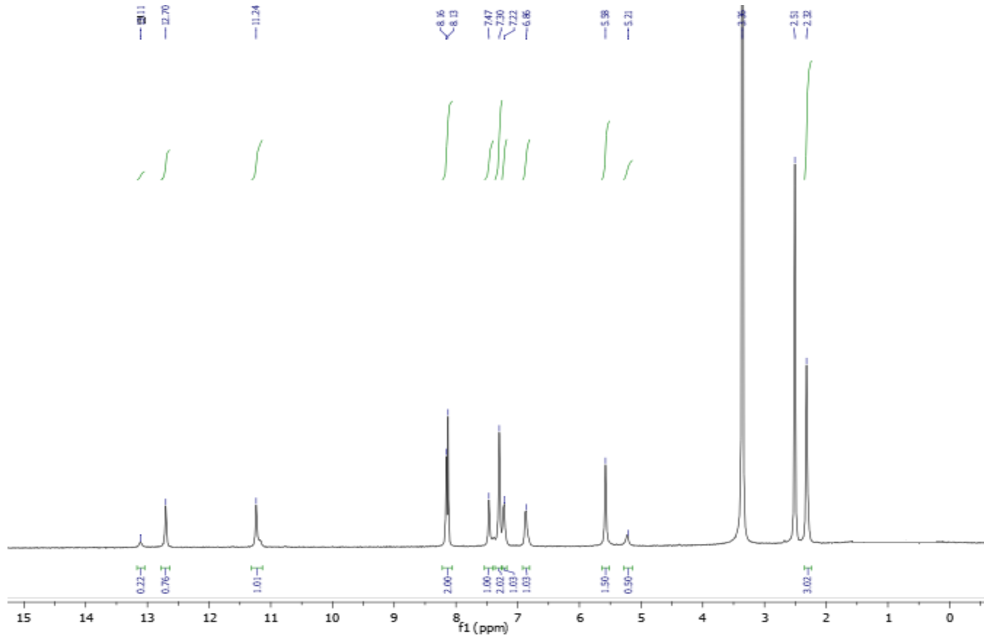

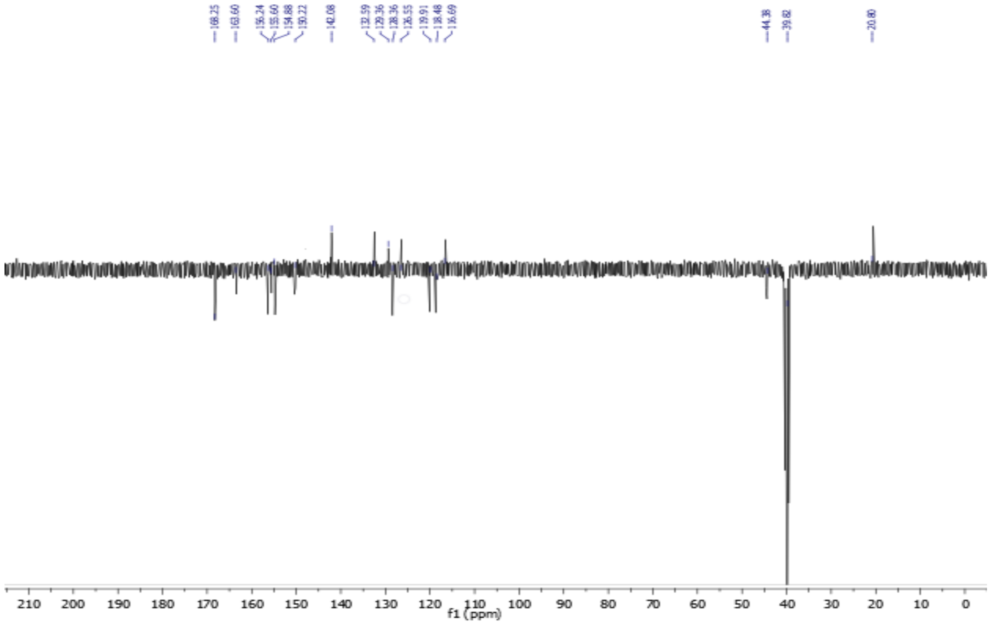
168.25
163.60
156.24
155.60
154.88
150.22
142.08
132.59
129.36
128.36
126.55
119.91
118.48
116.69
44.38
39.82
20.80
210 200 190 180 170 160 150 140 130 120 110 100 90 80 70 60 50 40 30 20 10 0
f1 (ppm)

2-(6-Amino-9*H*-purin-9-yl)-*N*'-(5-nitro-2-oxoindolin-3-ylidene)acetohydrazide (3f)

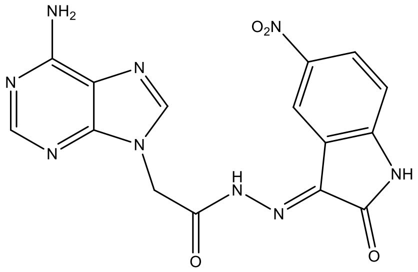

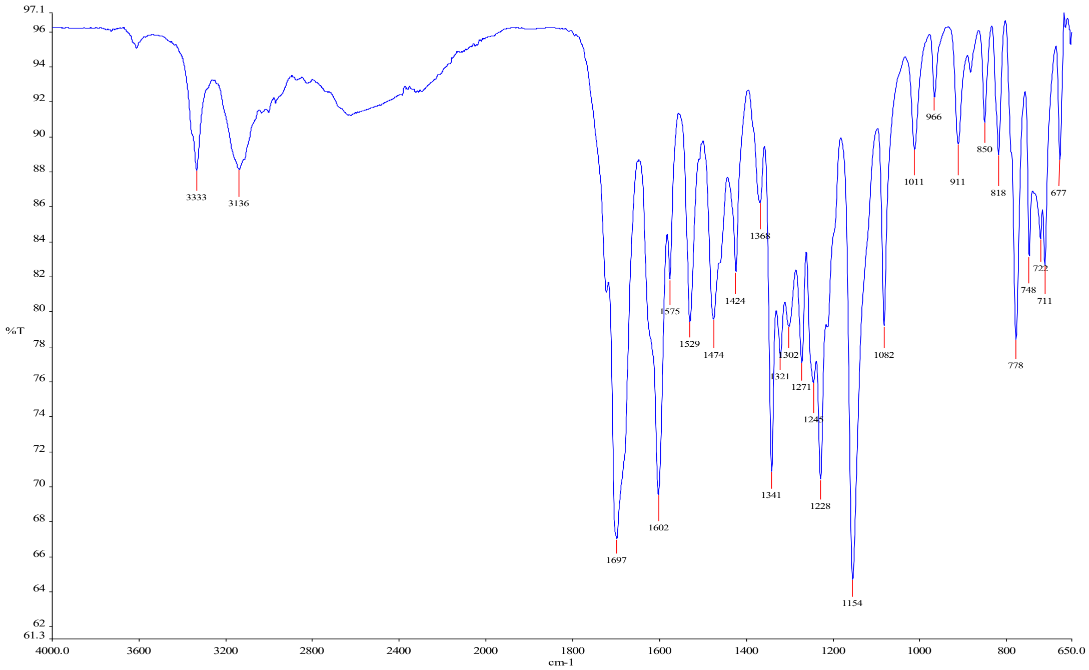

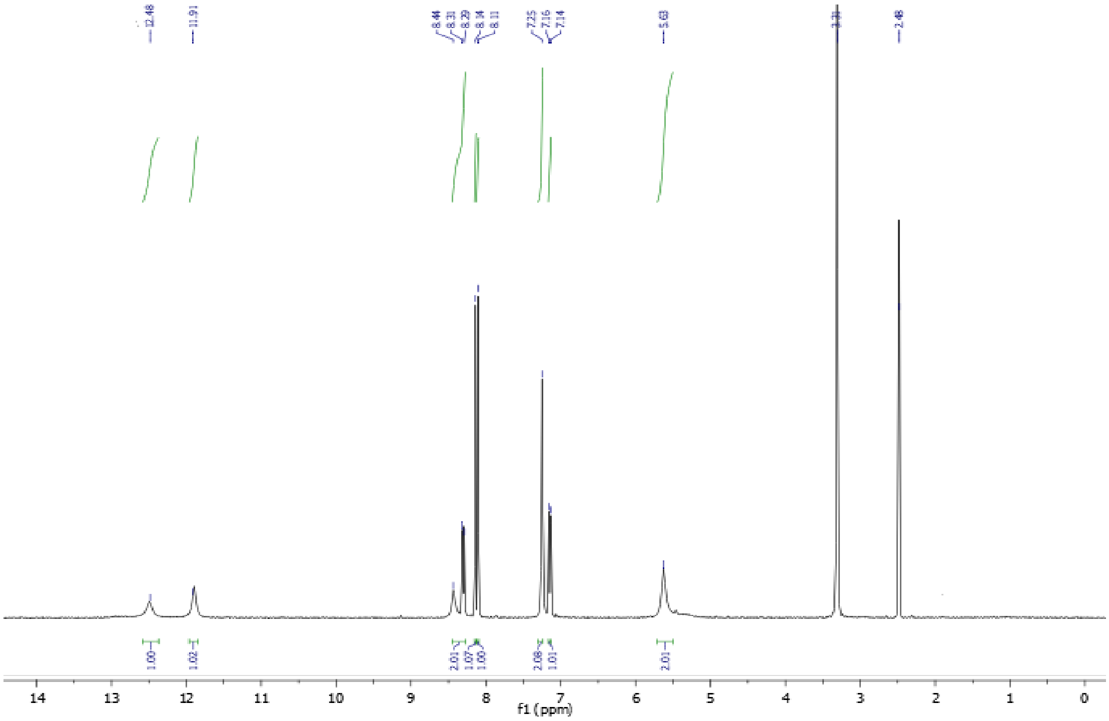

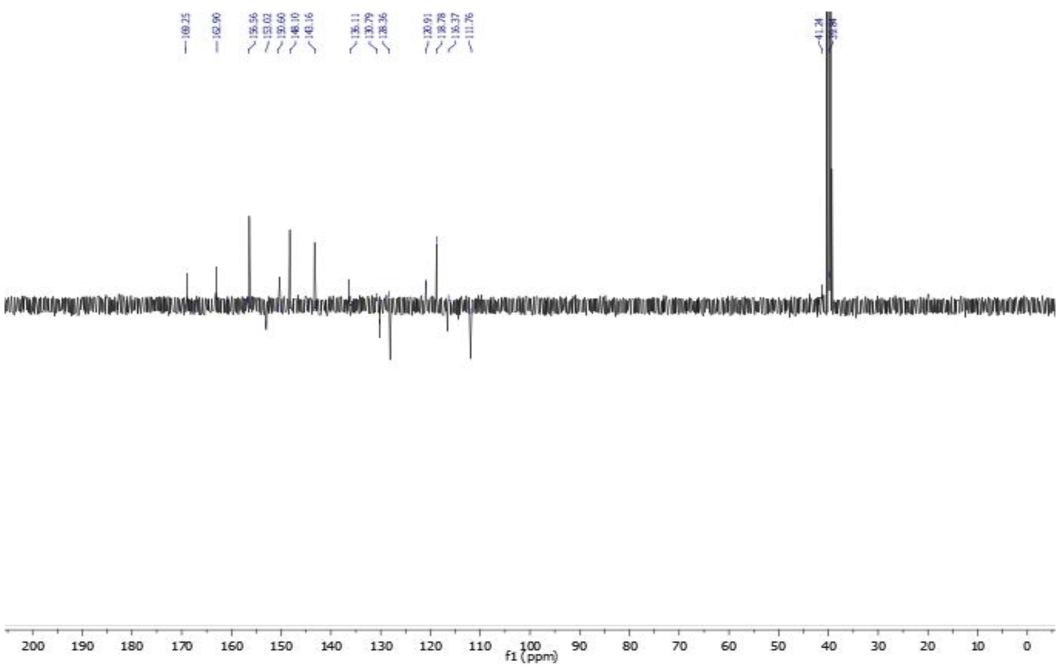

169.25
162.90
156.56
153.02
150.60
148.10
143.16
136.11
130.79
128.36
120.91
118.78
116.37
111.76
41.24
200
190
180
170
160
150
140
130
120
110
100
90
80
70
60
50
40
30
20
10
0
f1 (ppm)

2-(6-Amino-9*H*-purin-9-yl)-*N*'-(5-methoxy-2-oxoindolin-3-ylidene)acetohydrazide (3g)

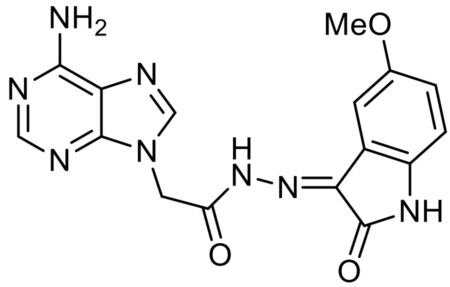

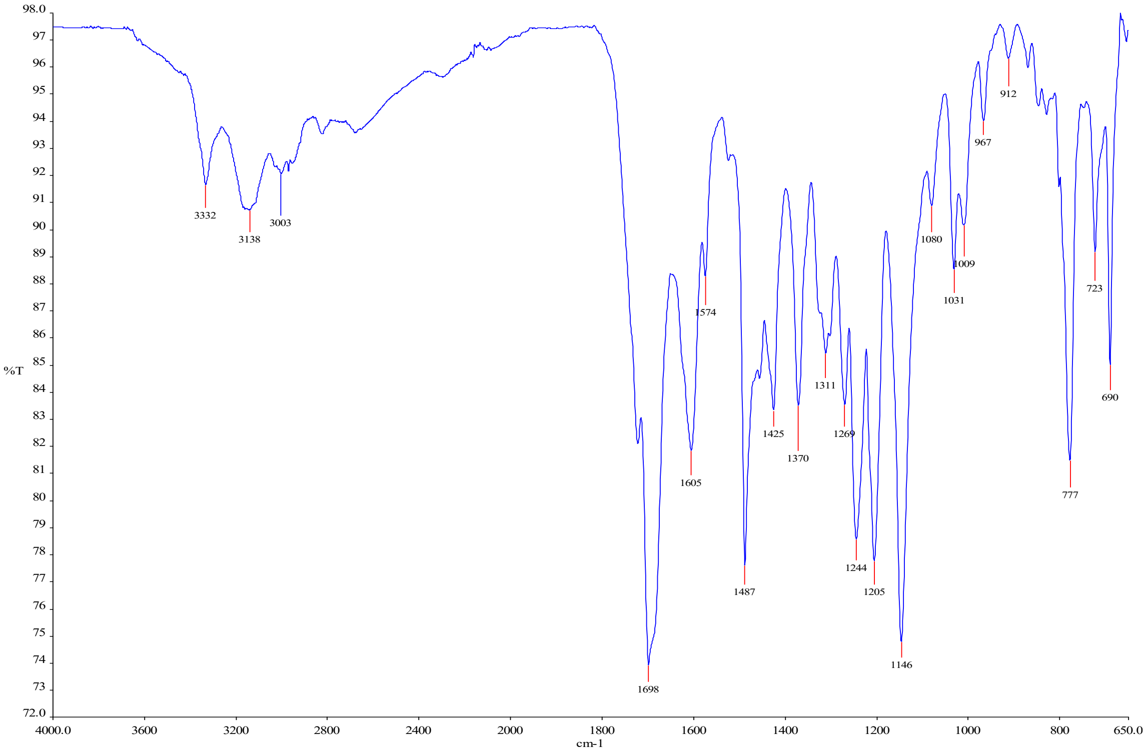

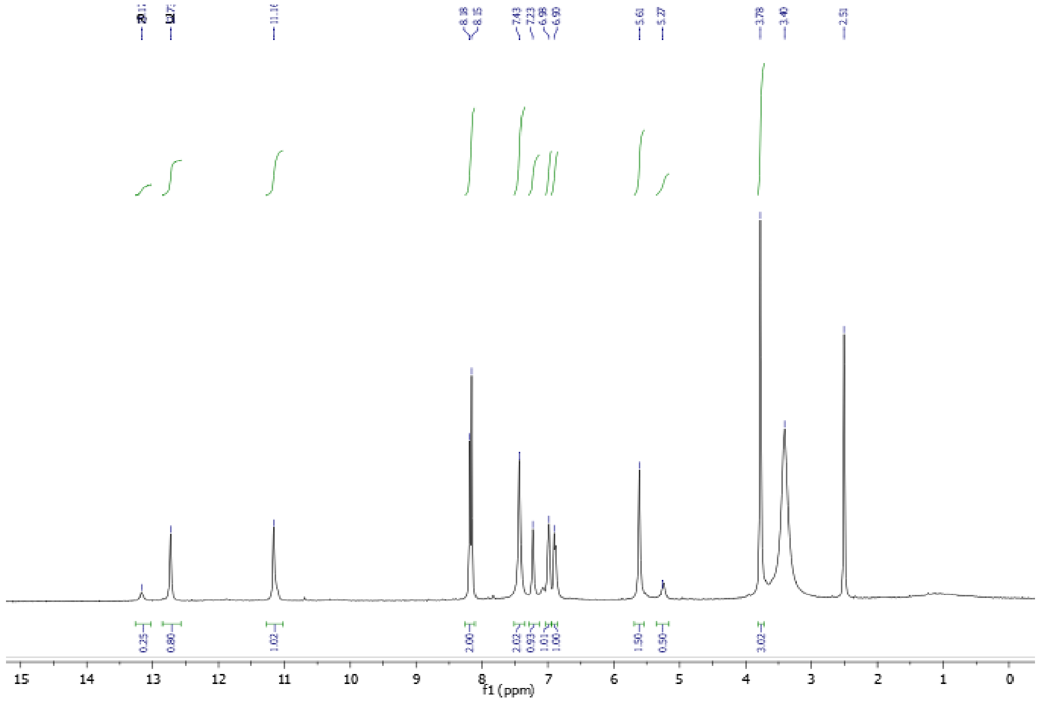
11.16
8.18
8.15
7.43
7.23
6.98
6.90
5.61
5.27
3.78
3.40
2.51
0.25
0.80
1.02
2.00
2.02
0.93
1.01
1.00
1.50
0.50
3.02
f1 (ppm)

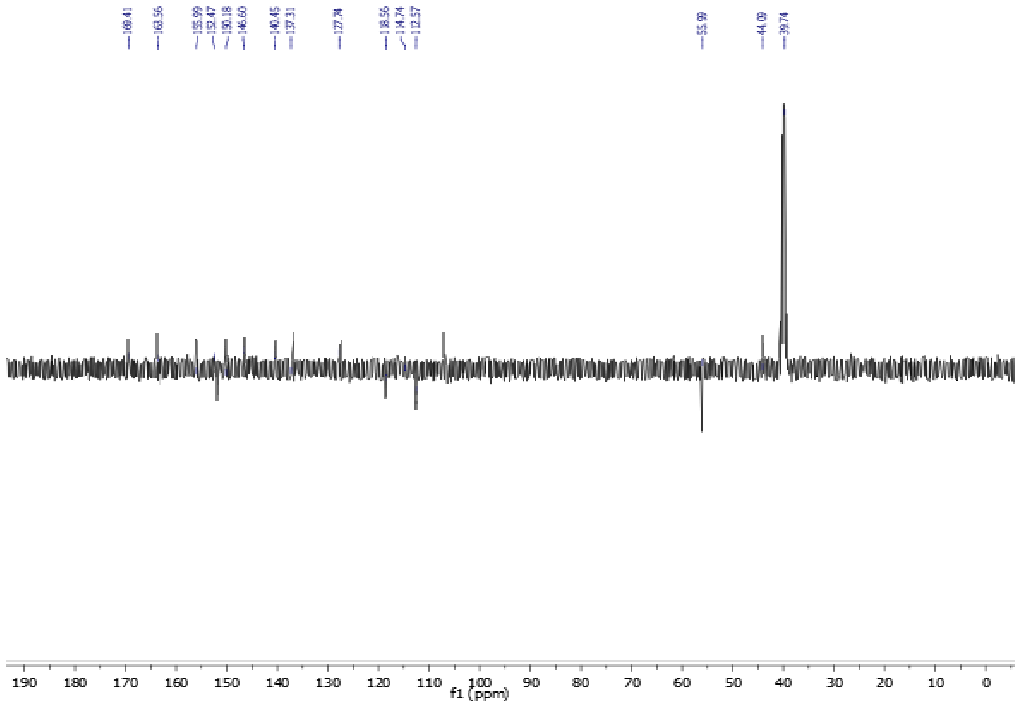
169.41
163.56
155.99
152.47
150.18
146.60
140.45
137.31
127.74
118.56
114.74
112.57
55.99
44.09
39.74
f1 (ppm)

2-(6-Amino-9*H*-purin-9-yl)-*N*'-(2-chlorobenzylidene)acetohydrazide (4a)

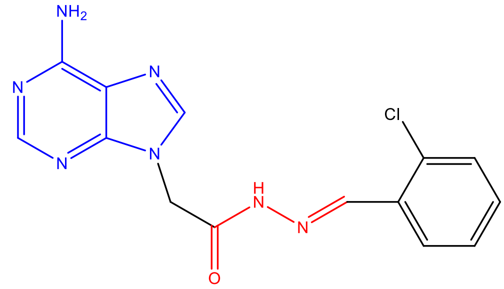

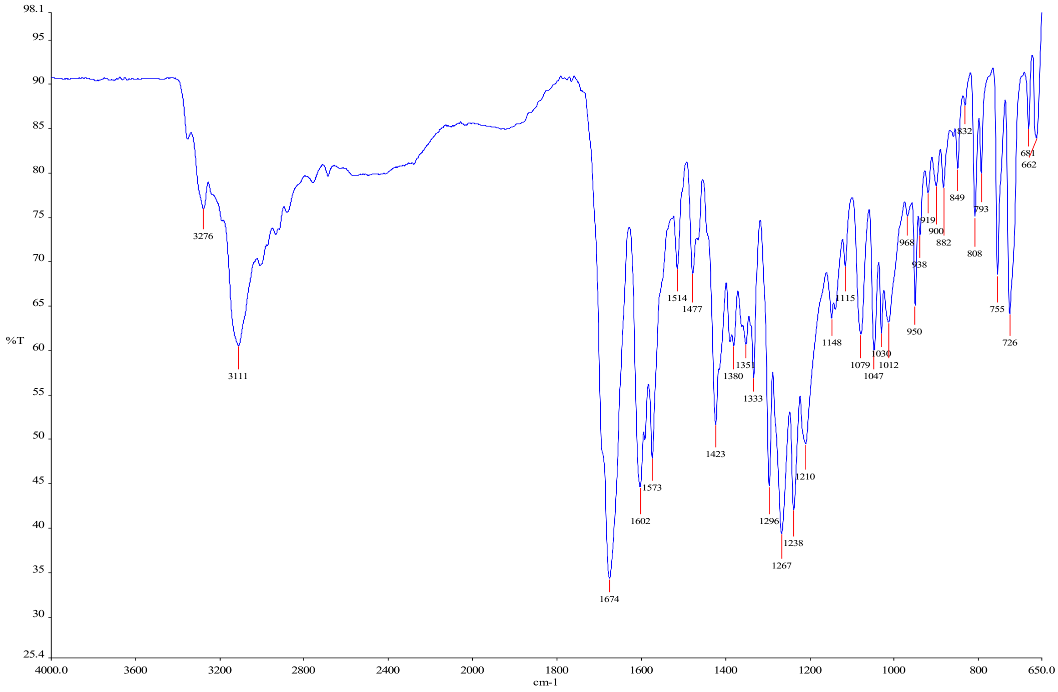

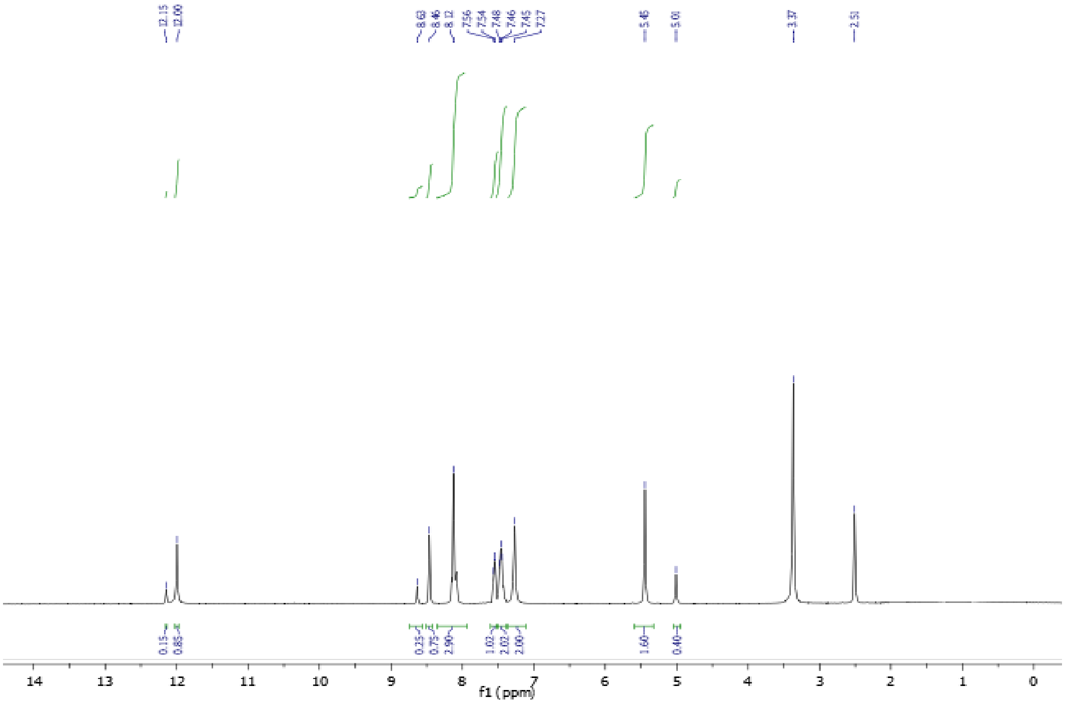

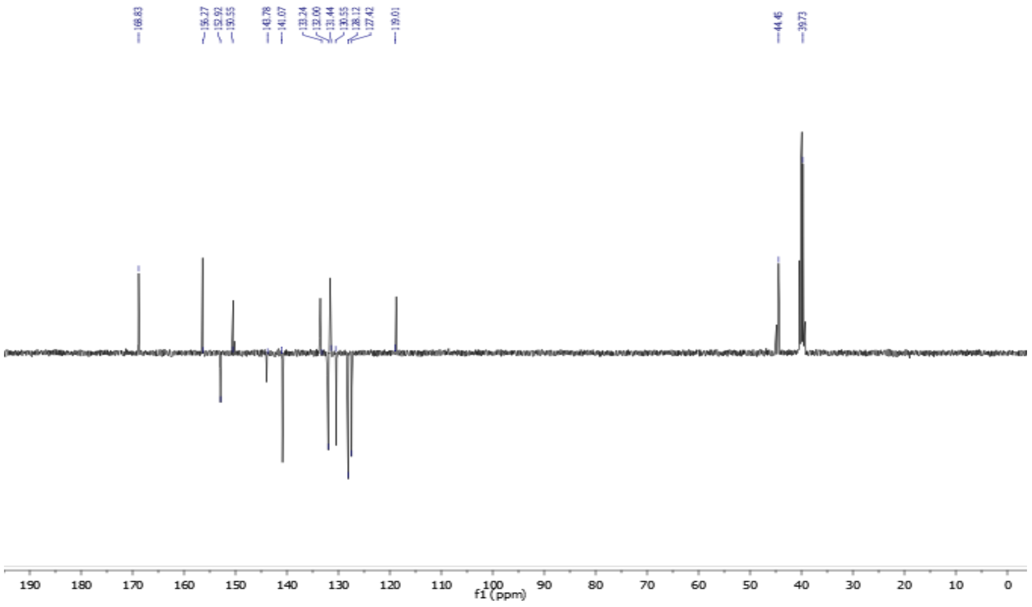

168.83
156.27
152.92
150.55
143.78
141.07
133.24
132.00
131.44
130.55
128.12
127.42
119.01
44.46
39.73
190 180 170 160 150 140 130 120 110 100 90 80 70 60 50 40 30 20 10 0
f1 (ppm)

2-(6-Amino-9*H*-purin-9-yl)-*N*'-(2-methylbenzylidene)acetohydrazide (4b)

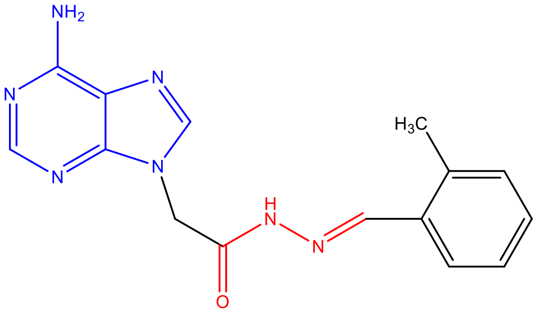

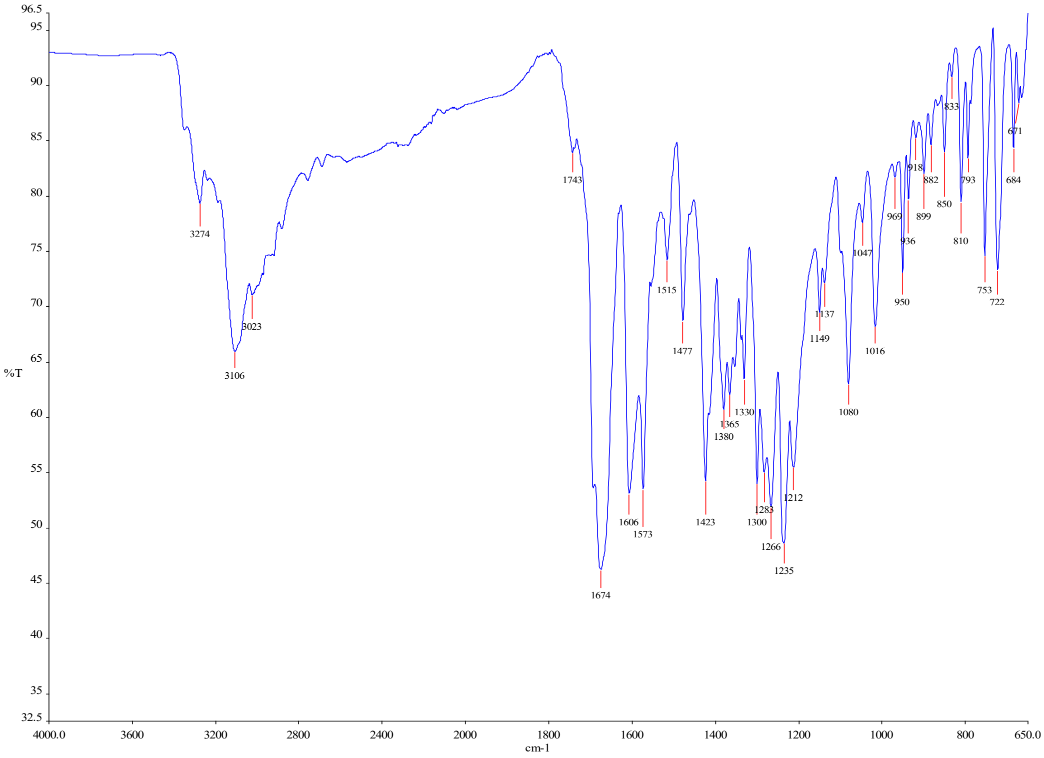

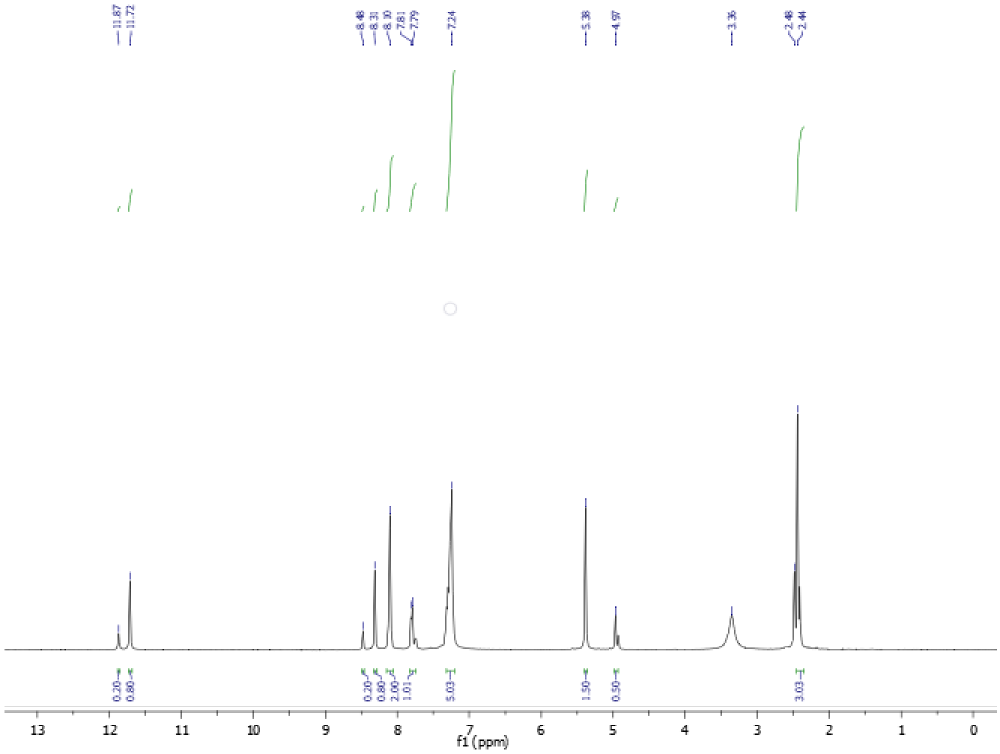
11.87
11.72
8.48
8.31
8.10
7.81
7.79
7.24
5.38
4.97
3.36
2.48
2.44
0.20
0.80
0.20
0.80
2.00
1.01
5.03
1.50
0.50
3.03
13 12 11 10 9 8 7 6 5 4 3 2 1 0
f1 (ppm)

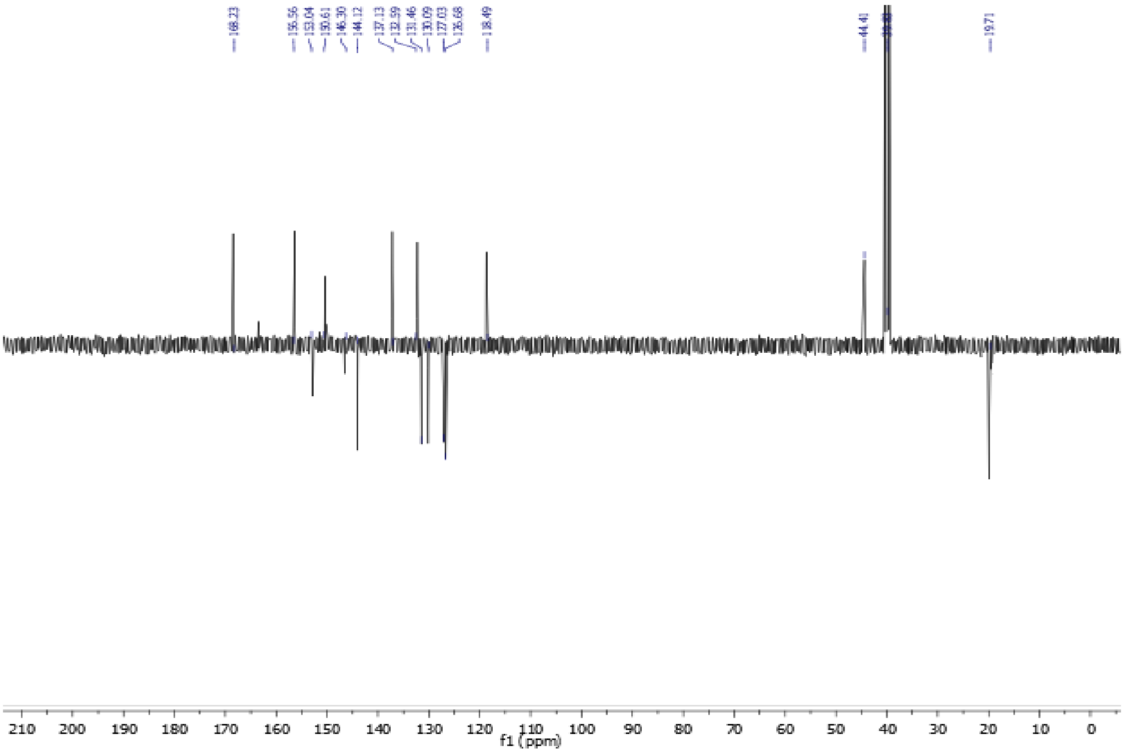
168.23
156.56
153.04
150.61
146.30
144.12
137.13
132.59
131.46
130.09
127.03
126.68
118.49
44.41
19.71
210 200 190 180 170 160 150 140 130 120 110 100 90 80 70 60 50 40 30 20 10 0
f1 (ppm)

2-(6-Amino-9*H*-purin-9-yl)-*N'*-(2,5-dichlorobenzylidene)acetohydrazide (4c)

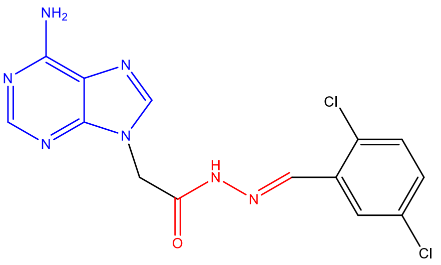

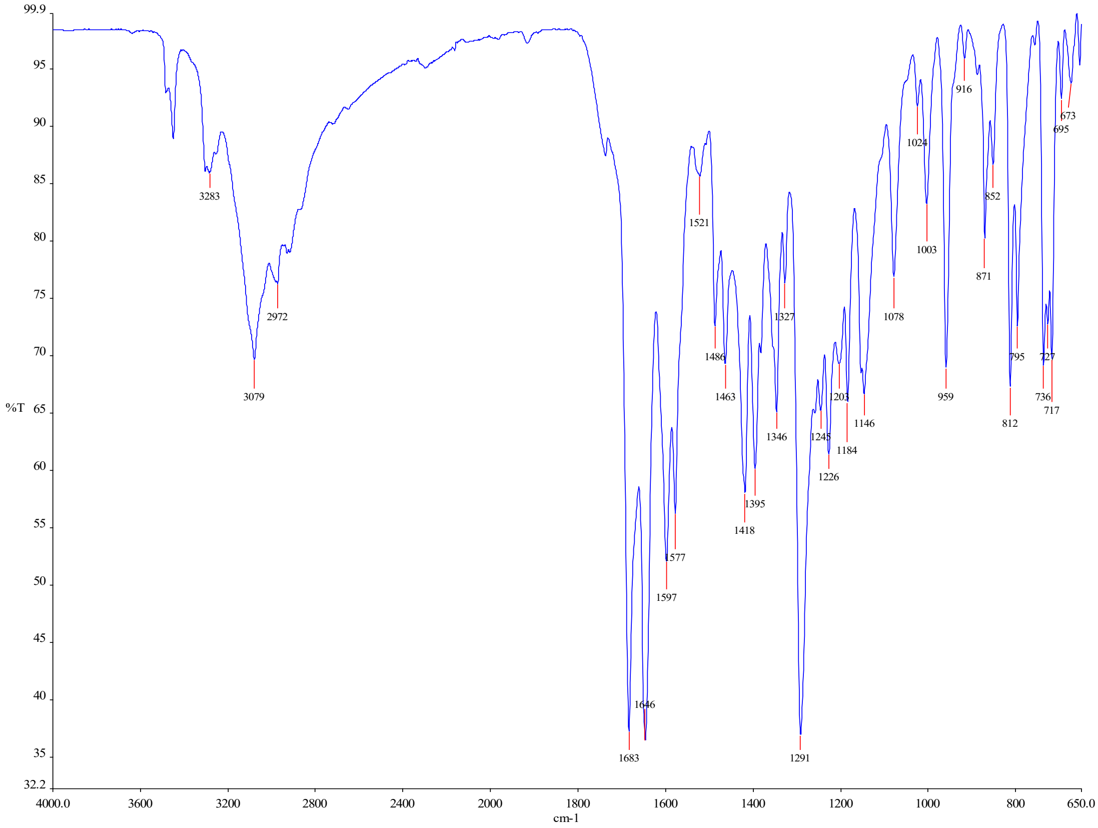

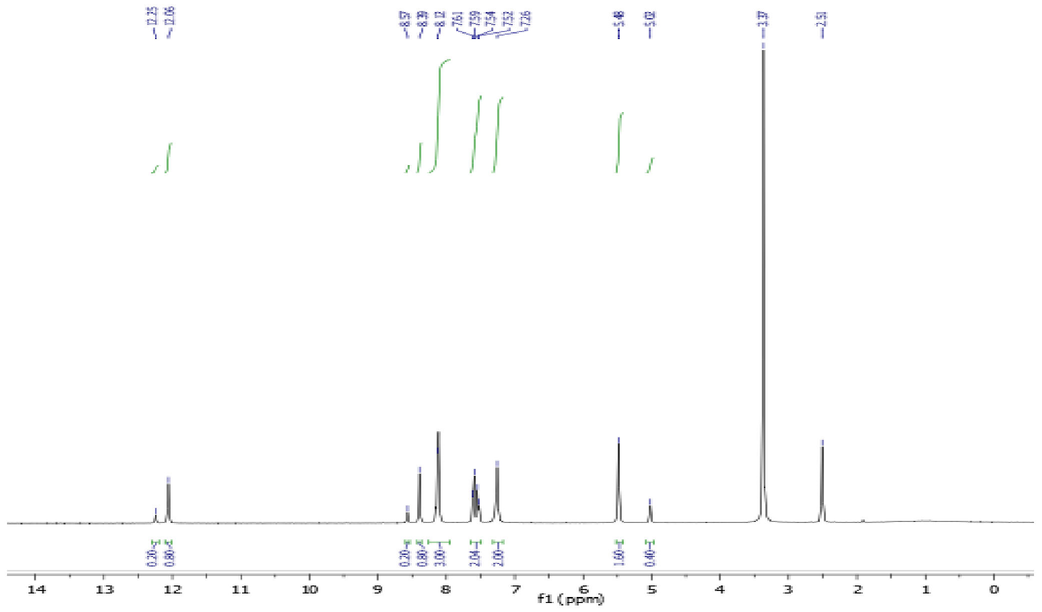

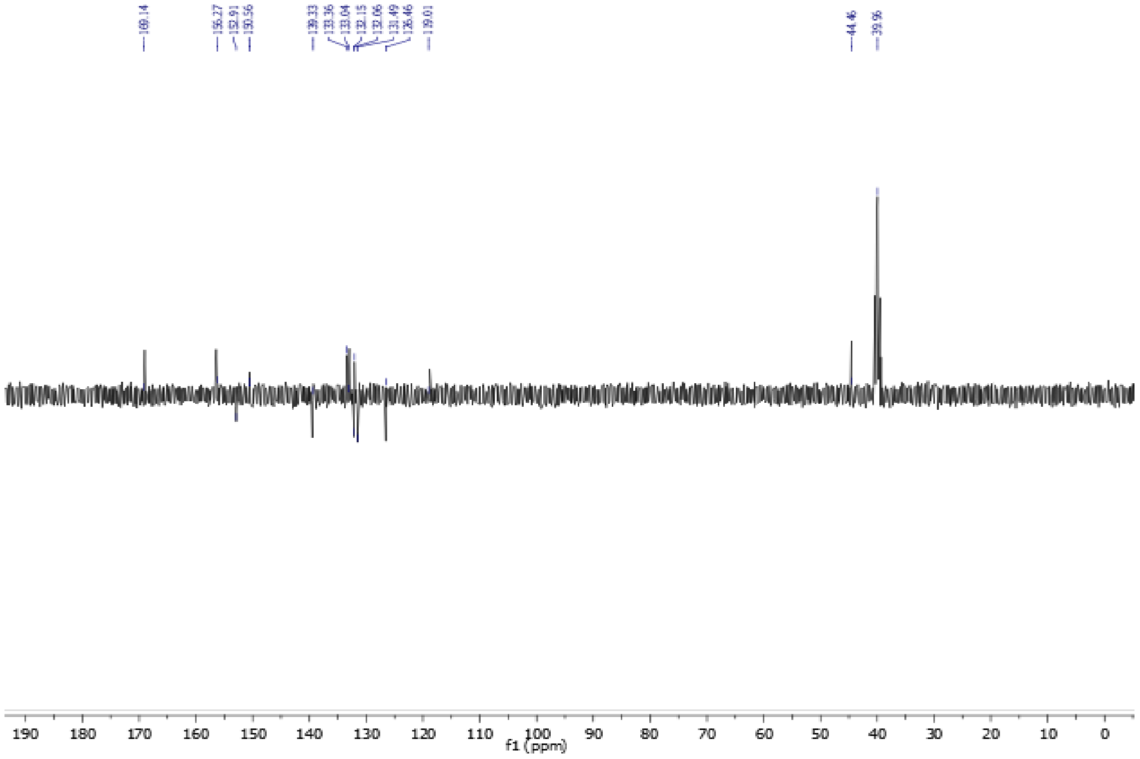

2-(6-Amino-9*H*-purin-9-yl)-*N*'-(2,6-dichlorobenzylidene)acetohydrazide (4d)

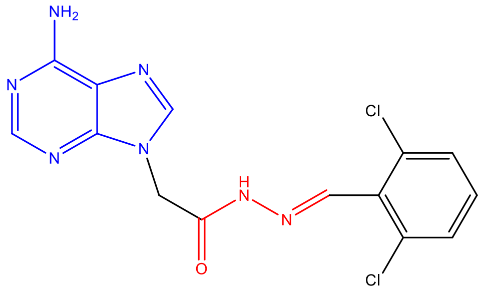

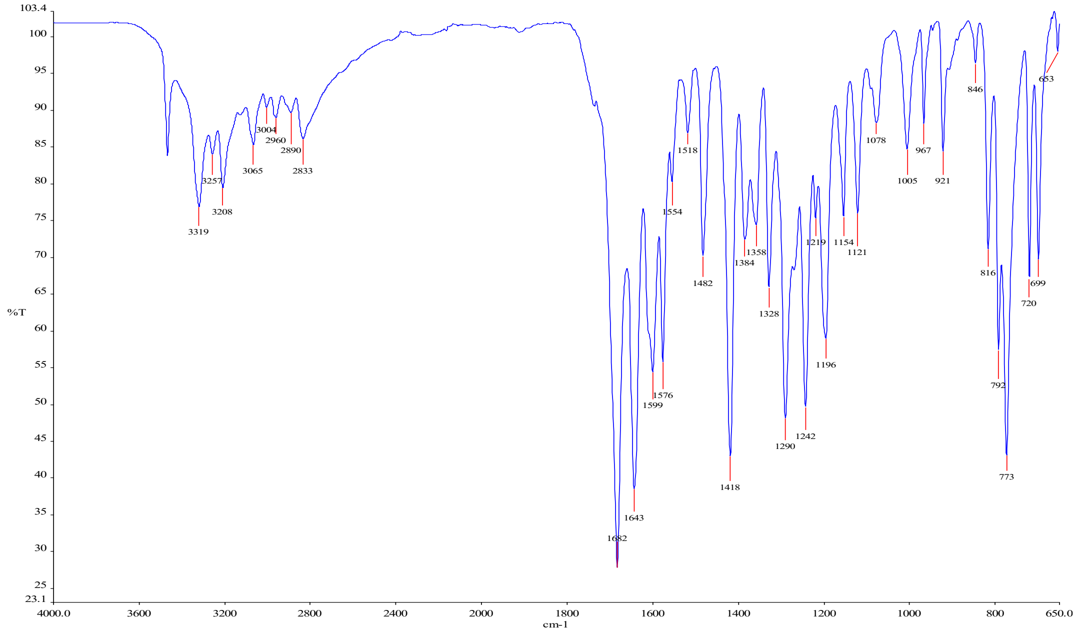

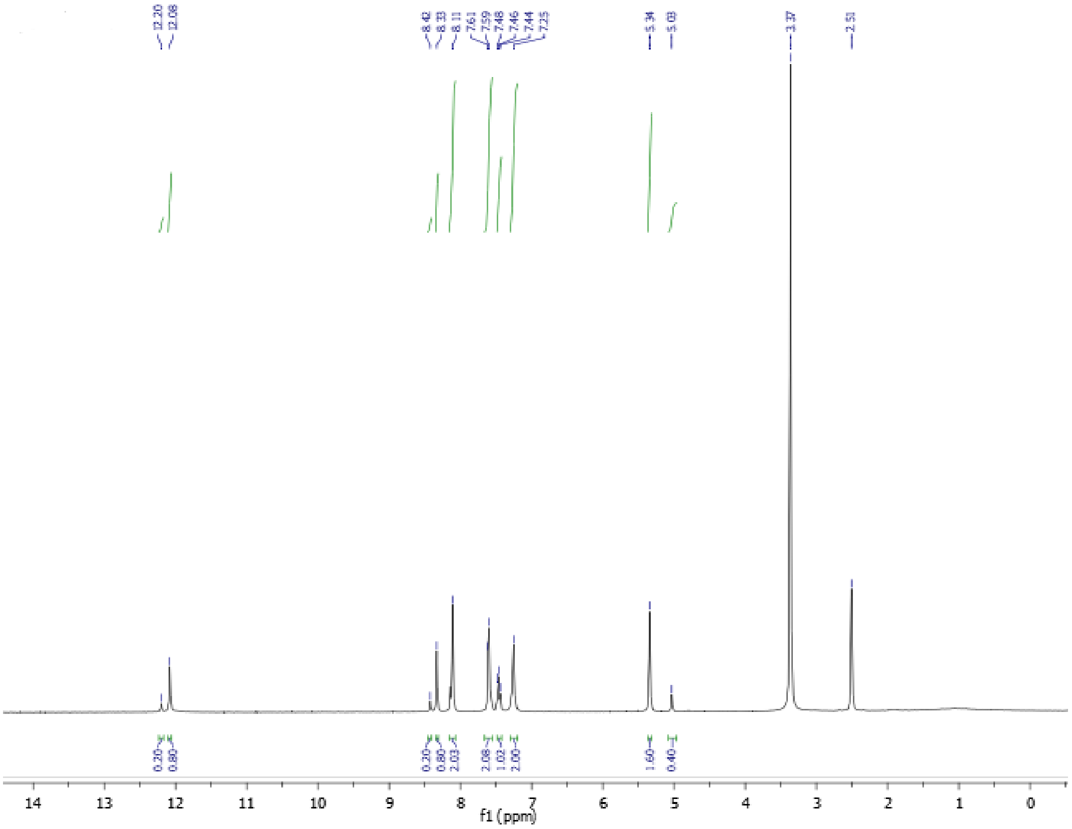
12.20
12.08
8.42
8.33
8.11
7.61
7.59
7.48
7.46
7.44
7.25
5.34
5.03
3.37
2.51
0.20
0.80
0.30
0.80
2.03
2.08
1.02
2.00
1.60
0.40
f1 (ppm)

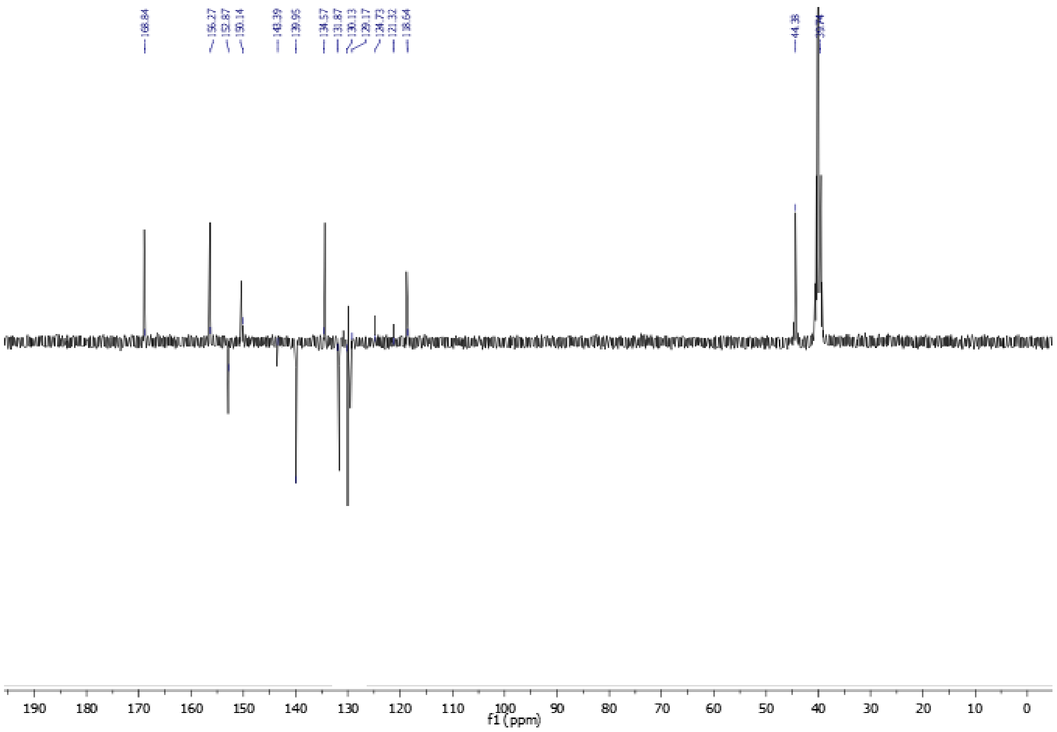
168.84
156.27
152.87
150.14
143.39
139.95
134.57
131.87
130.13
129.17
124.73
121.32
118.64
44.38
39.74
f1 (ppm)

2-(6-Amino-9*H*-purin-9-yl)-*N'*-(3-chlorobenzylidene)acetohydrazide (4e)

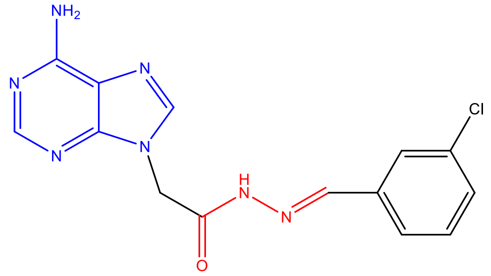

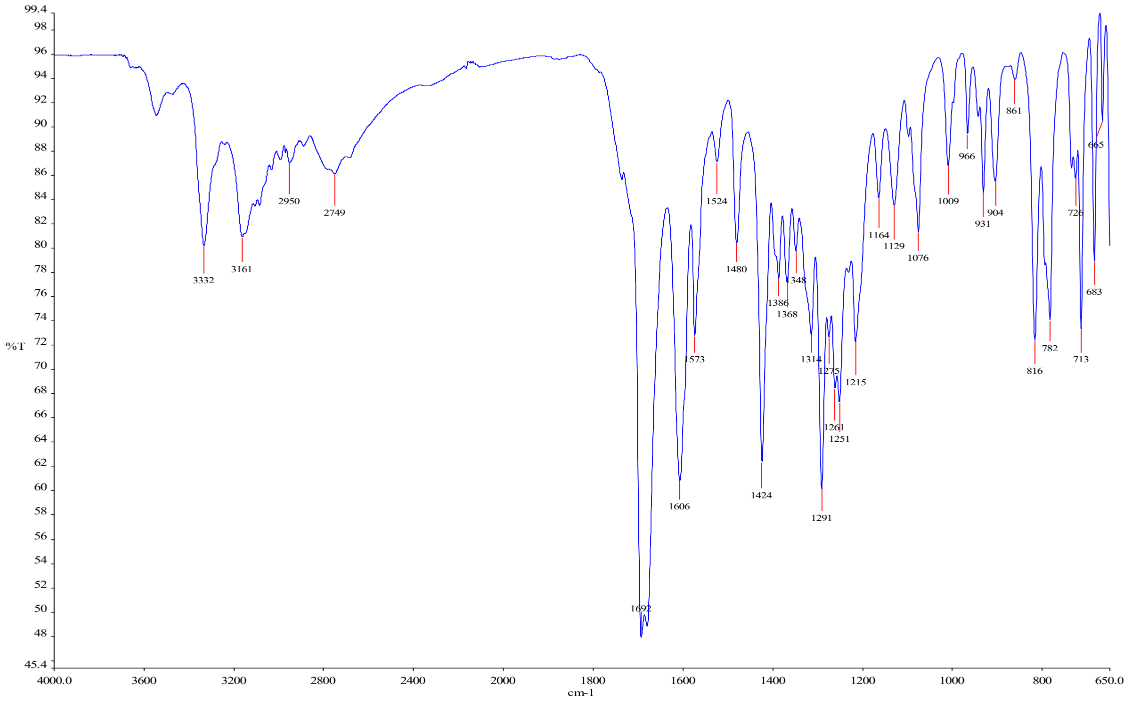

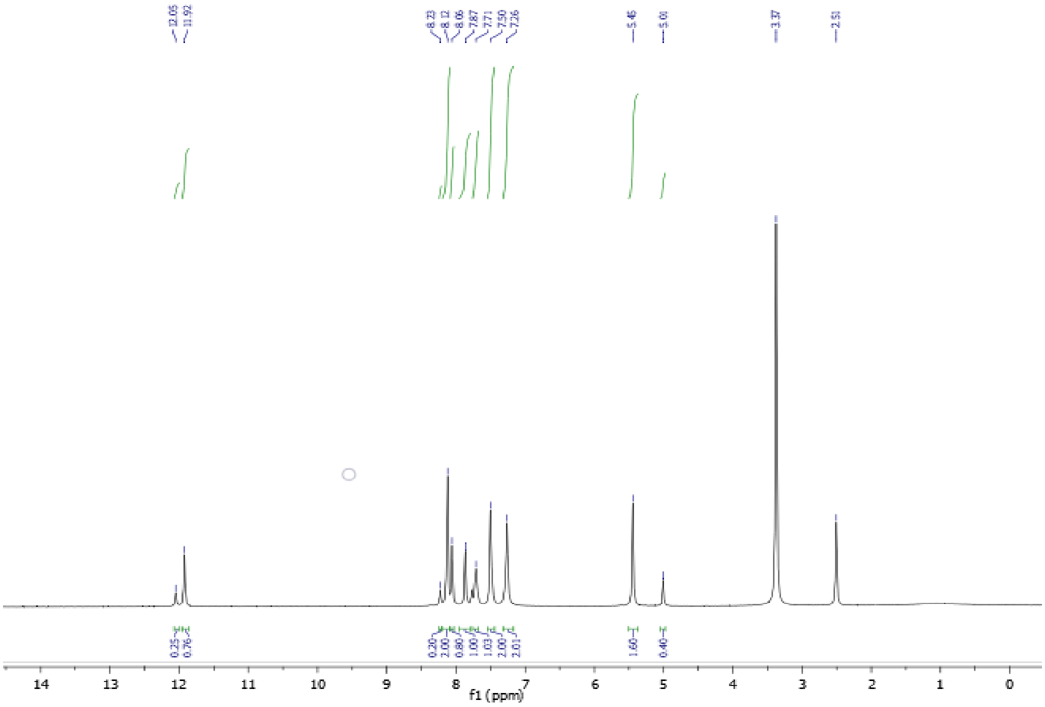

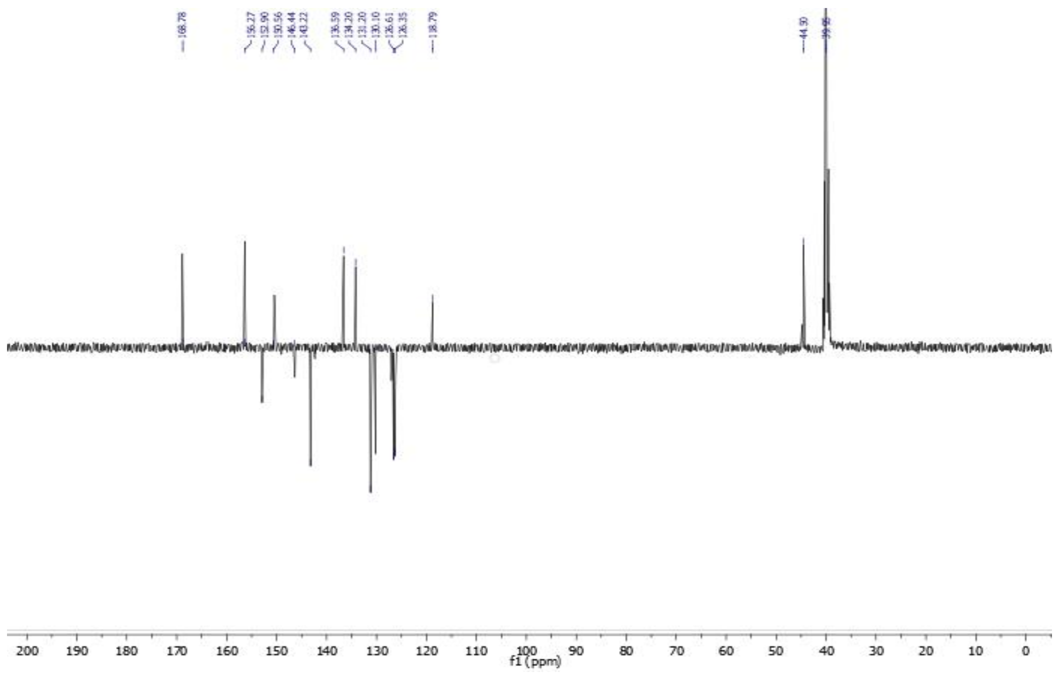
168.78
156.27
152.90
150.56
146.44
143.22
136.59
134.20
131.20
130.10
126.61
126.35
118.79
44.50
200
190
180
170
160
150
140
130
120
110
100
90
80
70
60
50
40
30
20
10
0
f1 (ppm)

2-(6-Amino-9*H*-purin-9-yl)-*N*'-(3-methylbenzylidene)acetohydrazide (4f)

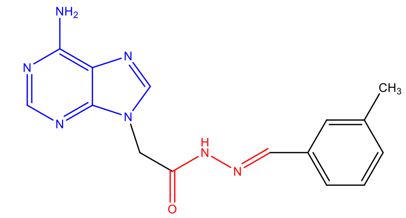

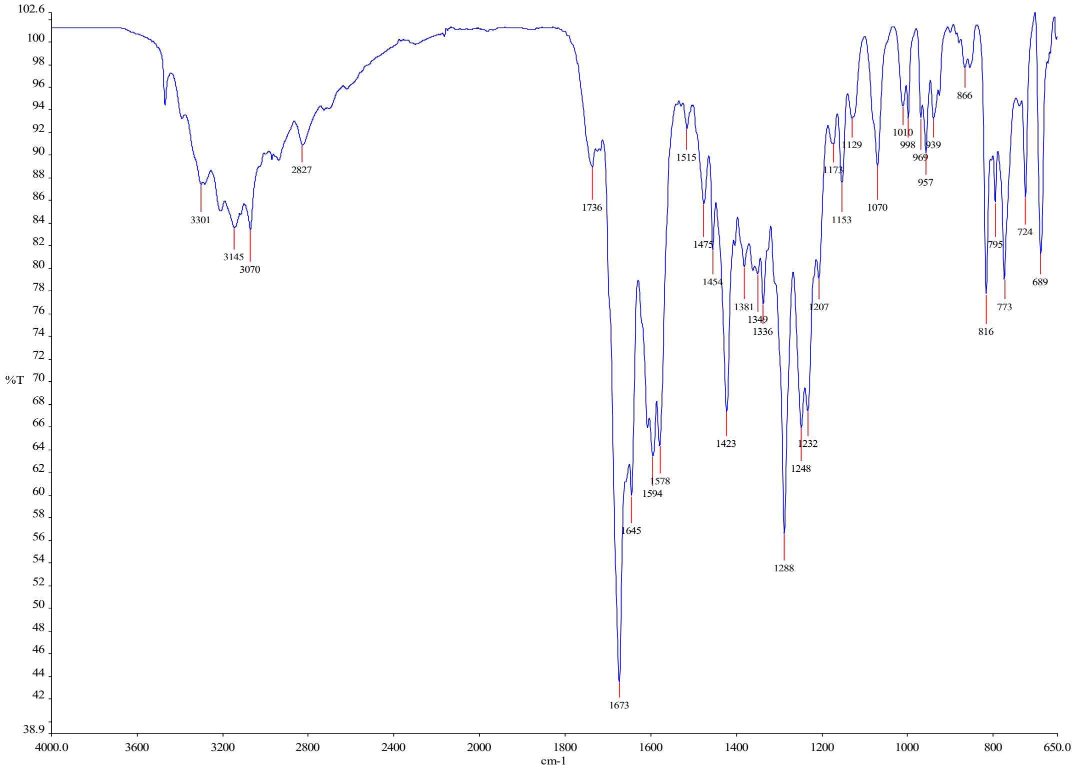

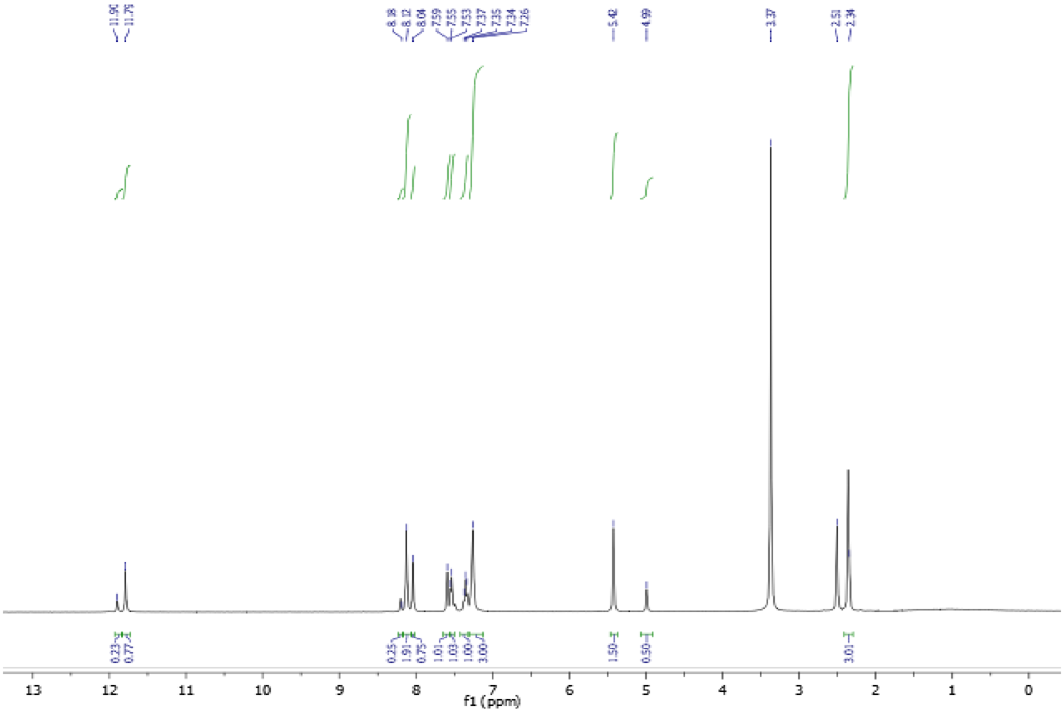

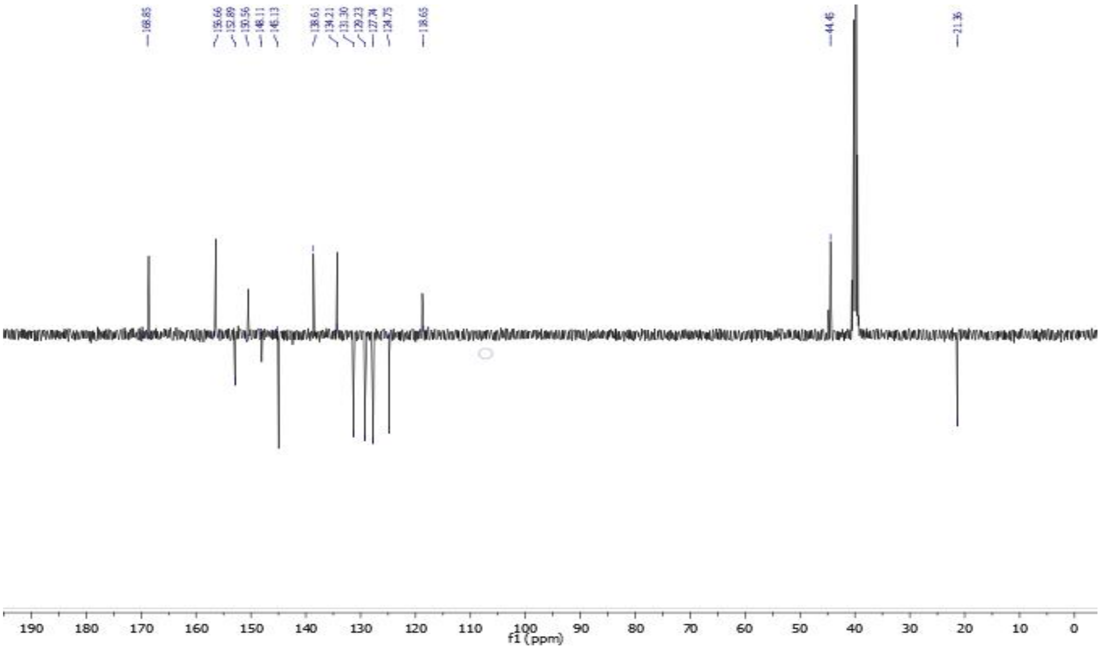

168.85
156.66
152.89
150.56
148.11
145.13
138.61
134.21
131.30
129.23
127.74
124.75
118.65
44.45
21.36
190 180 170 160 150 140 130 120 110 100 90 80 70 60 50 40 30 20 10 0
f1 (ppm)

2-(6-Amino-9*H*-purin-9-yl)-*N*'-(3-nitrobenzylidene)acetohydrazide (4g)

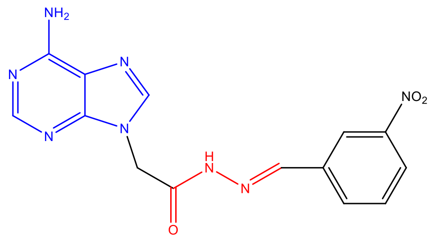

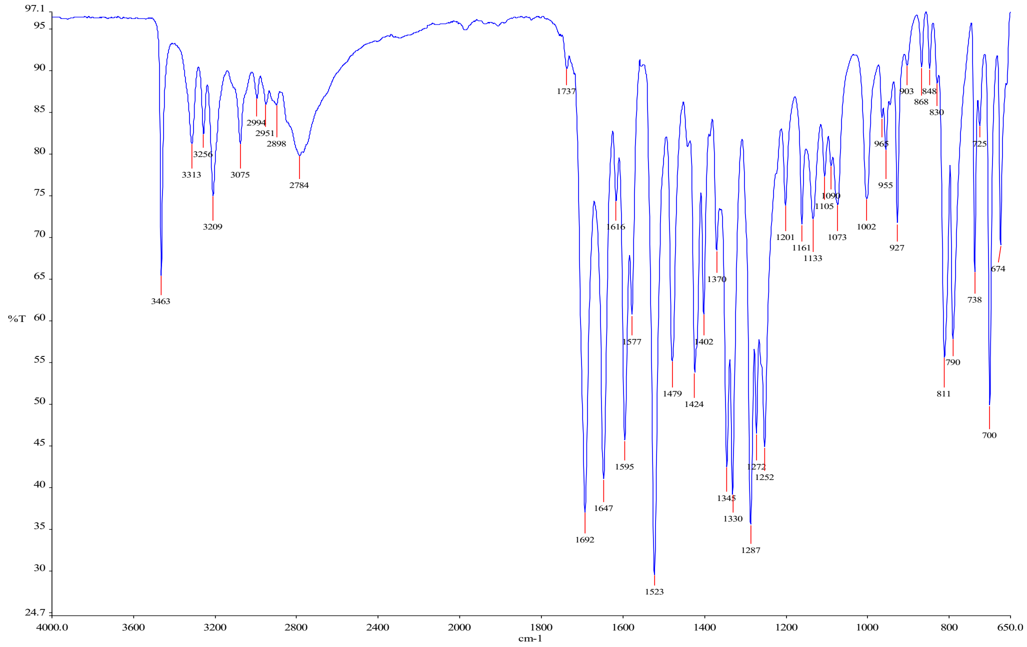

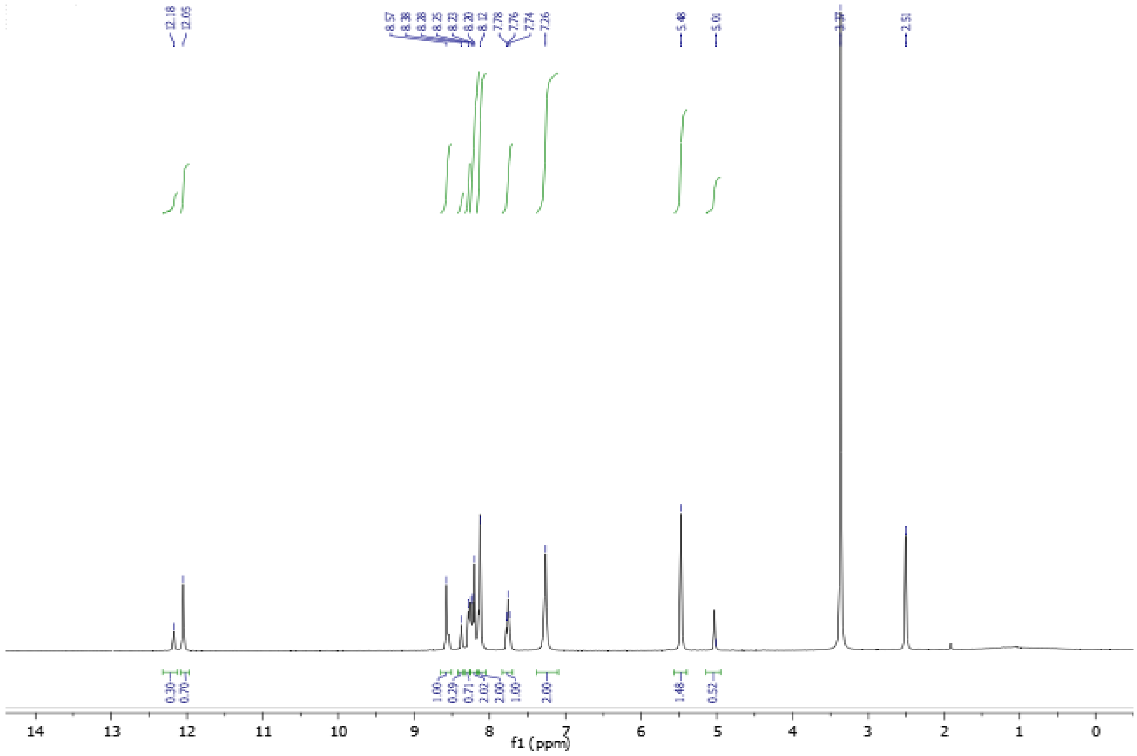

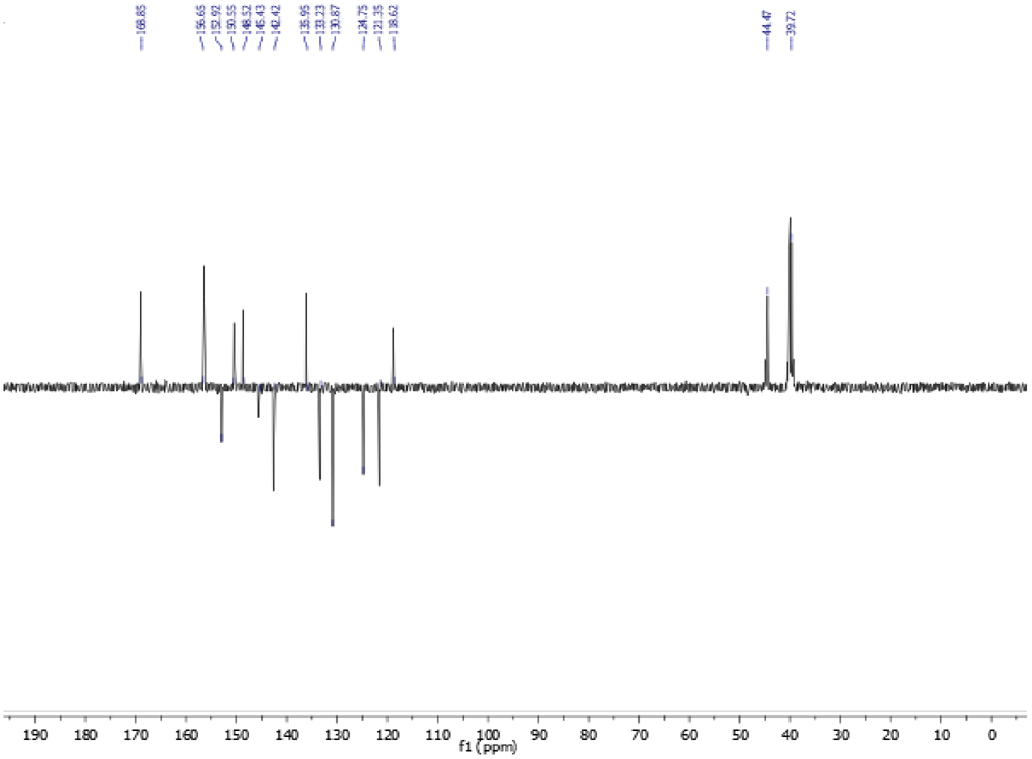
168.85
156.65
152.92
150.55
148.52
145.43
142.42
135.95
133.23
130.87
124.75
121.35
118.62
44.47
39.72
190
180
170
160
150
140
130
120
110
100
90
80
70
60
50
40
30
20
10
0
f1 (ppm)

2-(6-Amino-9*H*-purin-9-yl)-*N'*-(3-hydroxybenzylidene)acetohydrazide (4h)

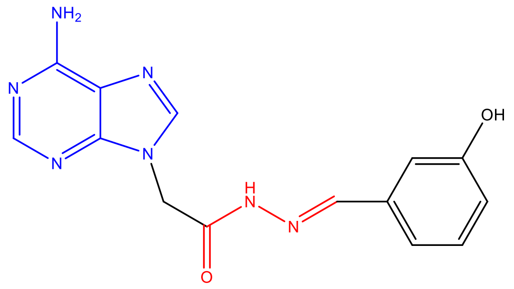

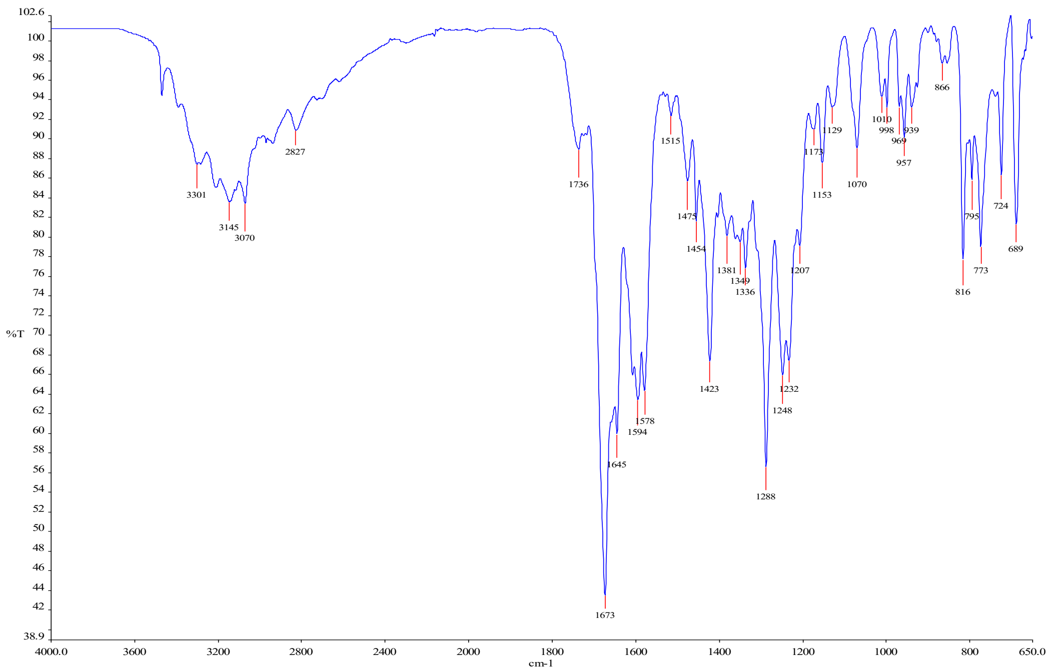

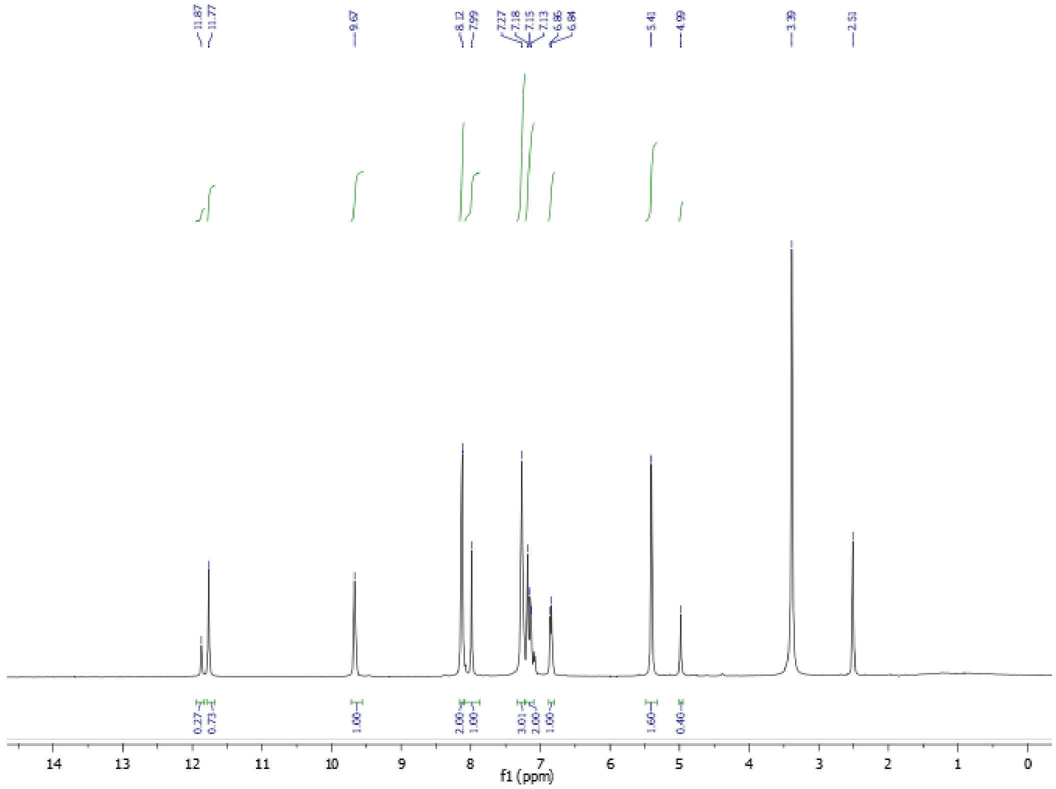

11.87
11.77
9.67
8.12
7.99
7.27
7.18
7.15
7.13
6.86
6.84
5.41
4.99
3.39
2.51
0.27
0.73
1.00
2.00
1.00
3.01
2.00
1.00
1.60
0.40
14 13 12 11 10 9 8 7 6 5 4 3 2 1 0
f1 (ppm)

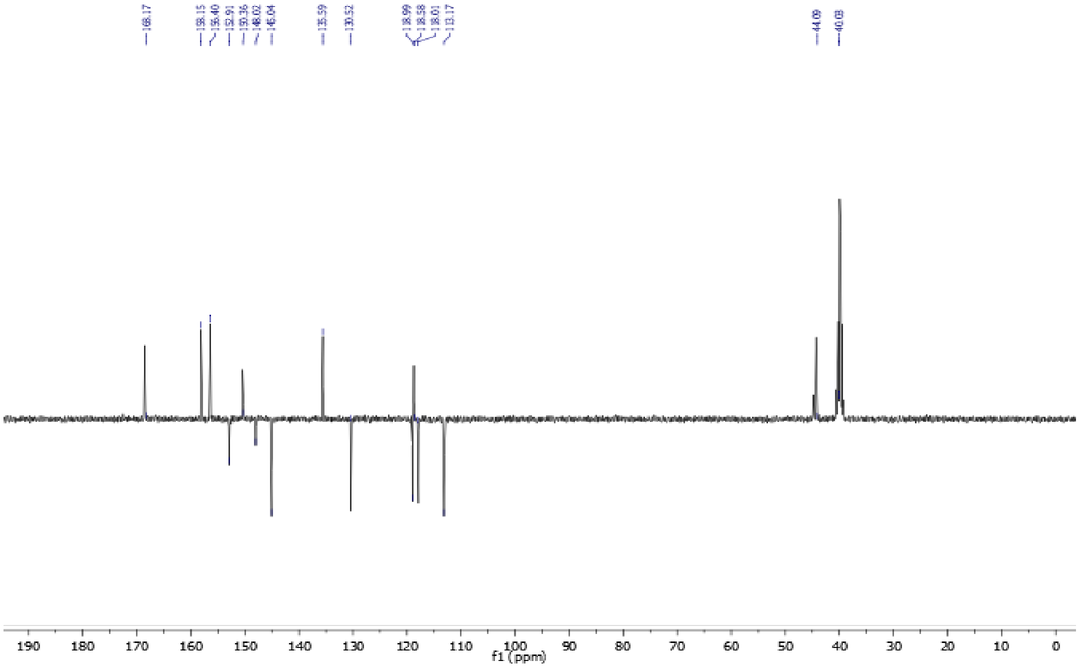

168.17
158.15
156.40
152.91
150.36
148.02
145.04
135.59
130.52
118.99
118.58
118.01
113.17
44.09
40.03
190 180 170 160 150 140 130 120 110 100 90 80 70 60 50 40 30 20 10 0
f1 (ppm)

2-(6-Amino-9*H*-purin-9-yl)-*N*'-(thiophen-2-ylmethylene)acetohydrazide (4i)

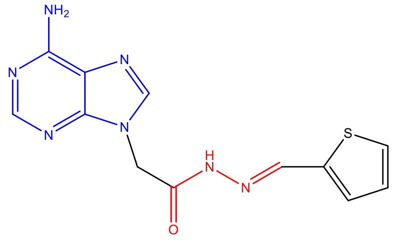

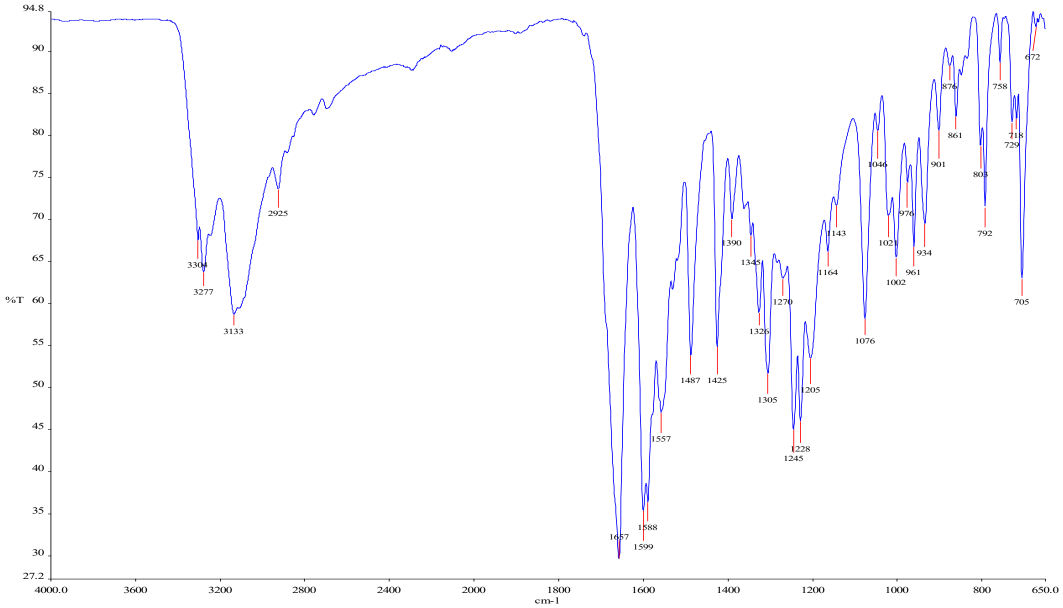

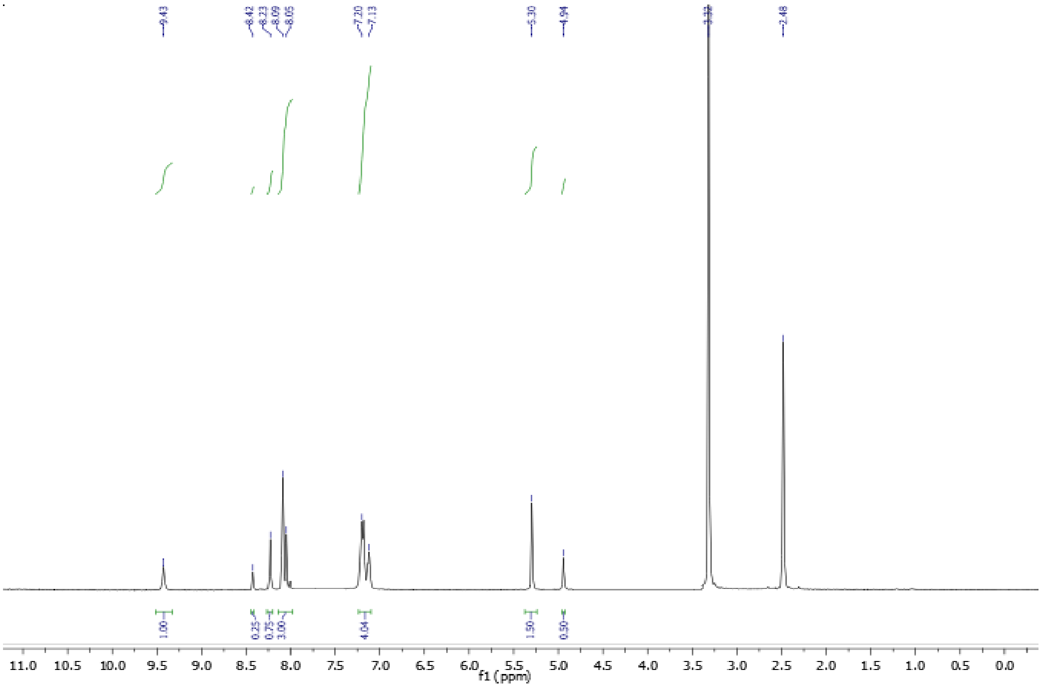

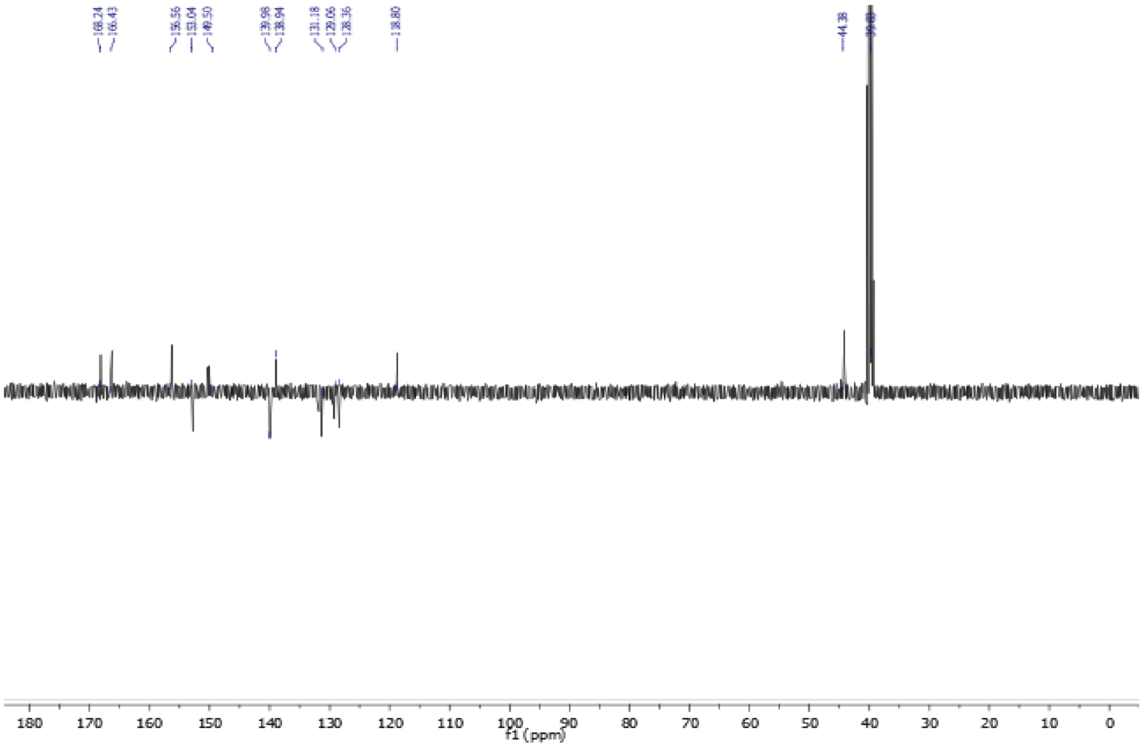

168.24
166.43
155.56
153.04
149.50
139.98
138.94
131.18
129.06
128.36
118.80
44.38
180
170
160
150
140
130
120
110
100
90
80
70
60
50
40
30
20
10
0
f1 (ppm)

2-(6-Amino-9*H*-purin-9-yl)-*N*'-(4-methylbenzylidene)acetohydrazide (4j)

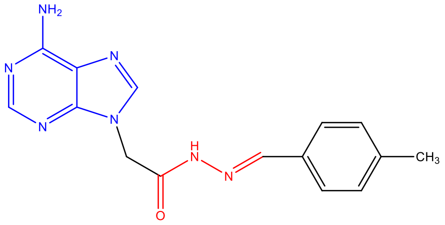

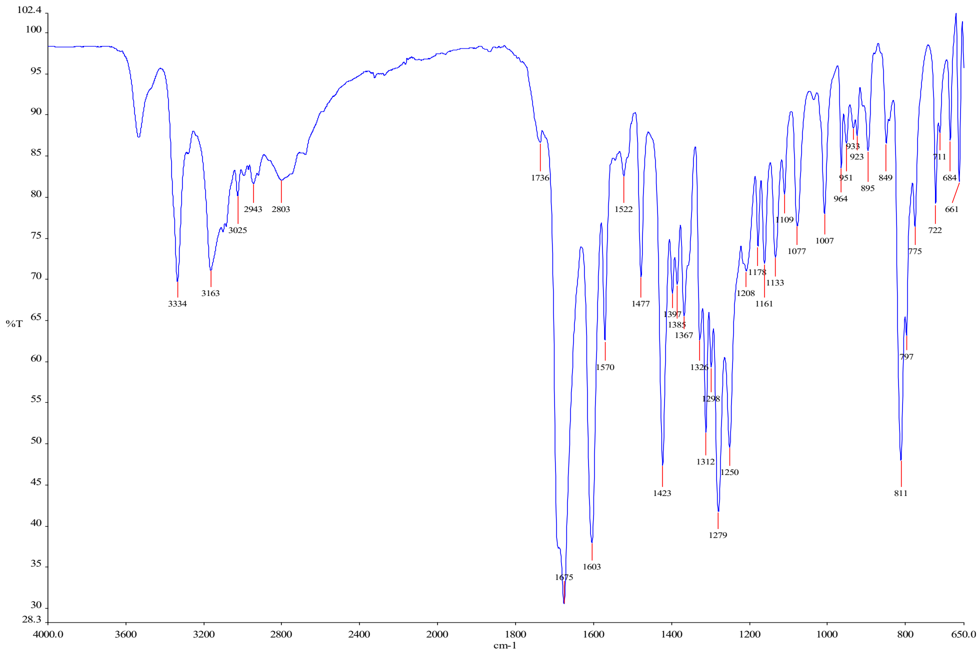

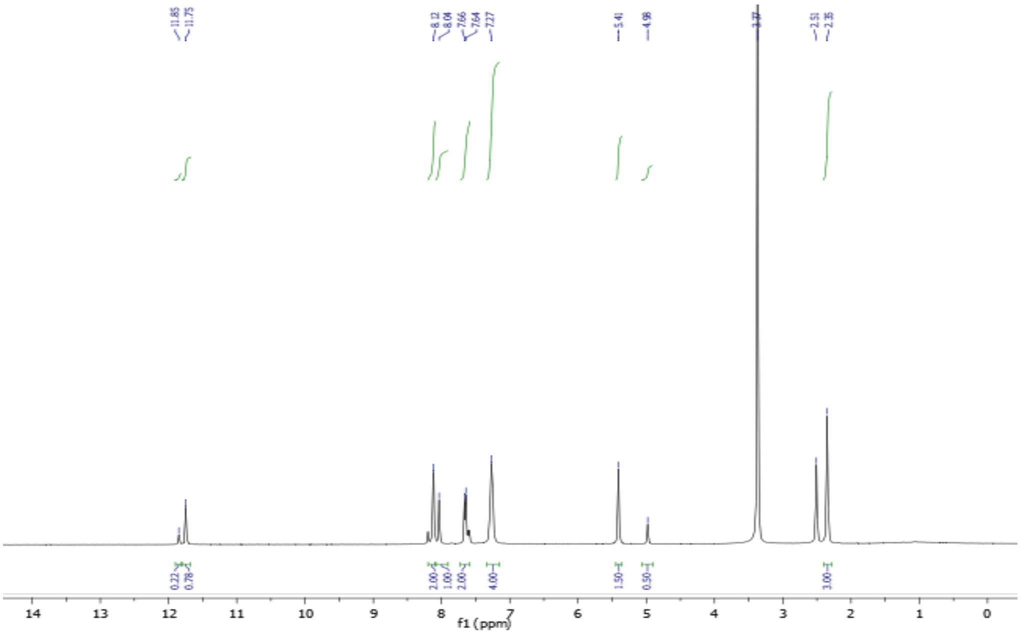

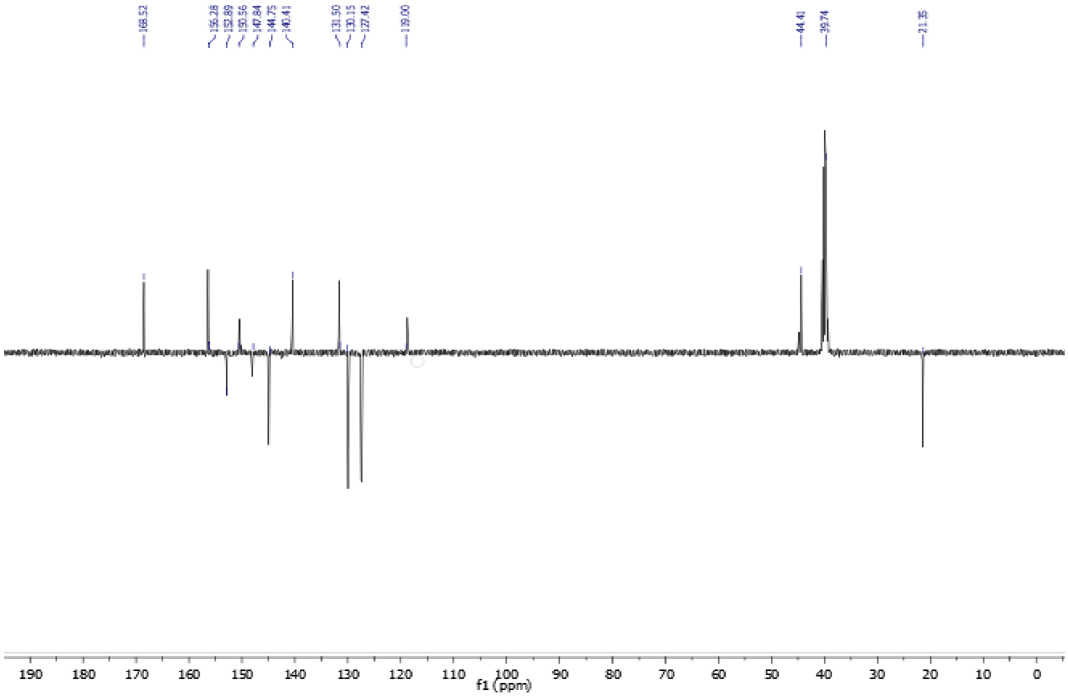
168.52
156.28
152.89
150.56
147.84
144.75
140.41
131.50
130.15
127.42
119.00
44.41
39.74
21.35
190 180 170 160 150 140 130 120 110 100 90 80 70 60 50 40 30 20 10 0
f1 (ppm)

2-(6-Amino-9*H*-purin-9-yl)-*N*'-(pyridin-2-ylmethylene)acetohydrazide (4k)

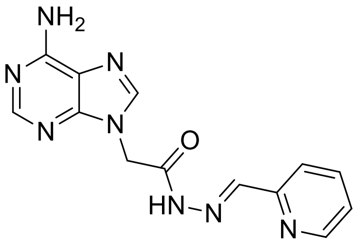

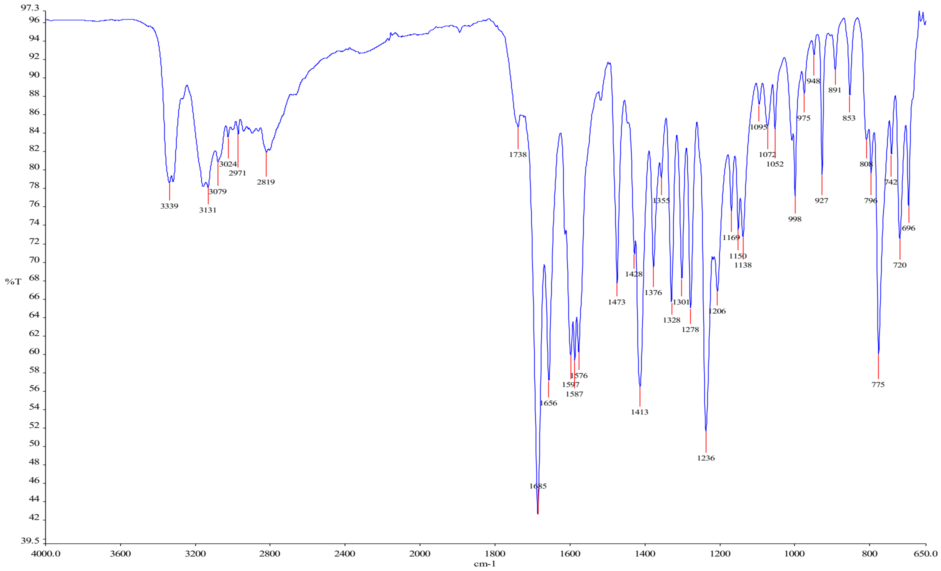

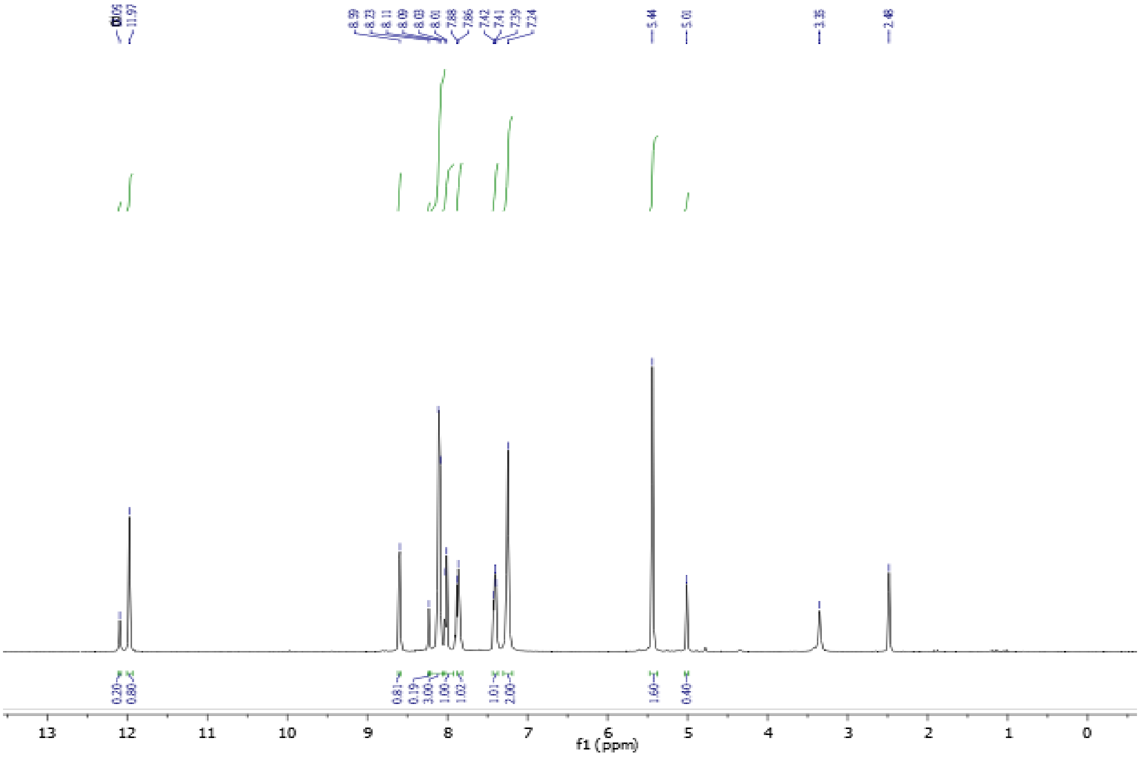

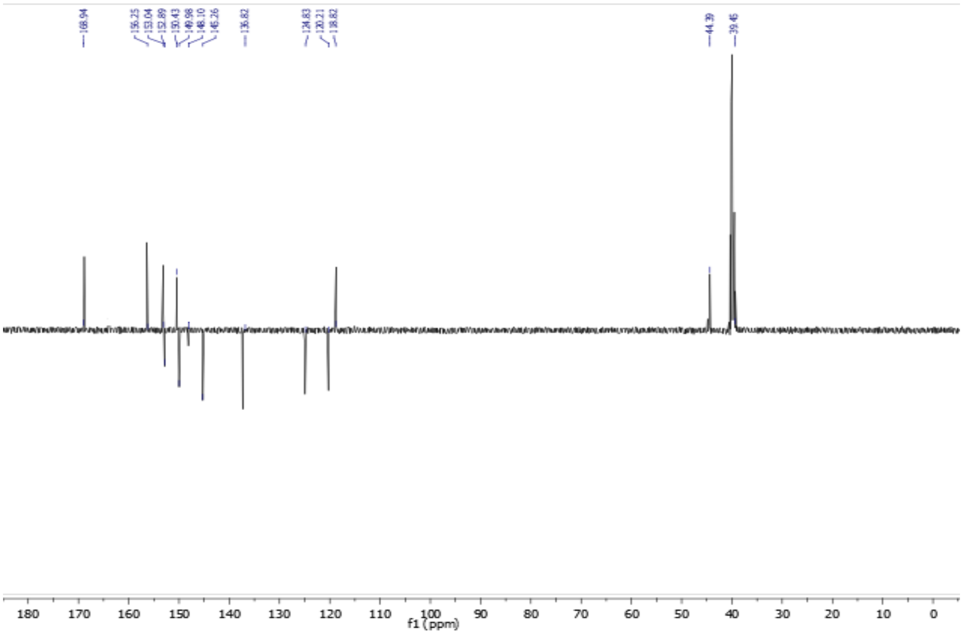
168.94
156.25
153.04
152.89
150.43
149.98
148.10
145.26
136.82
124.83
120.21
118.82
44.39
39.45
180
170
160
150
140
130
120
110
100
90
80
70
60
50
40
30
20
10
0
f1 (ppm)

2-(6-Amino-9*H*-purin-9-yl)-*N*'-(4-hydroxybenzylidene)acetohydrazide (4l)

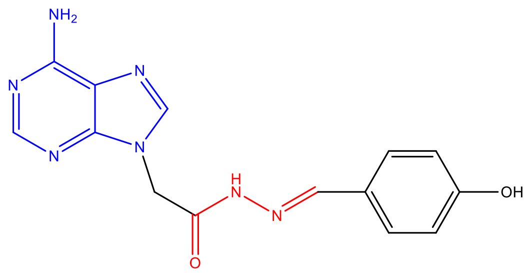

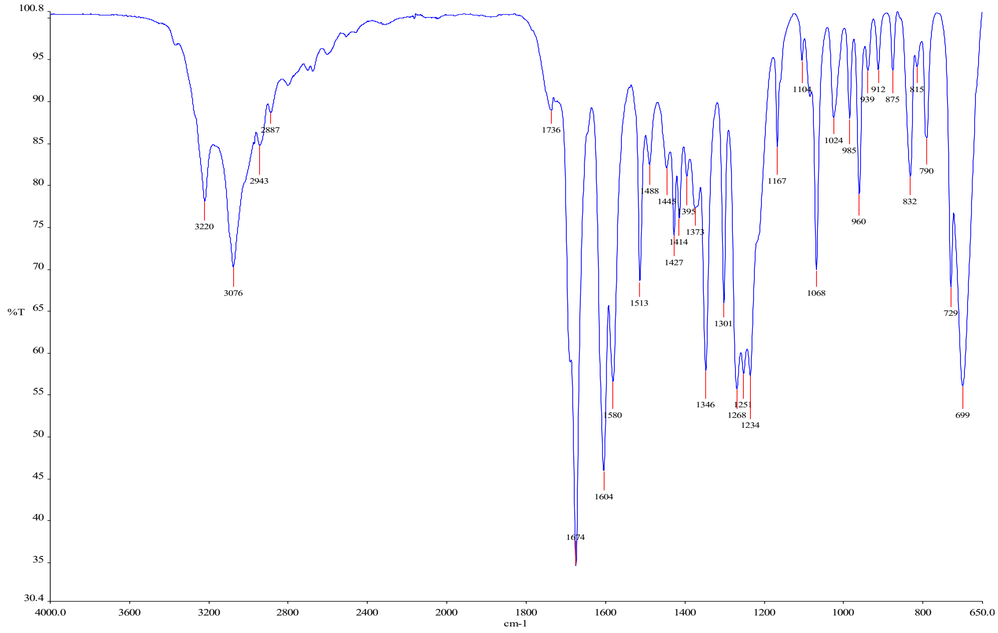

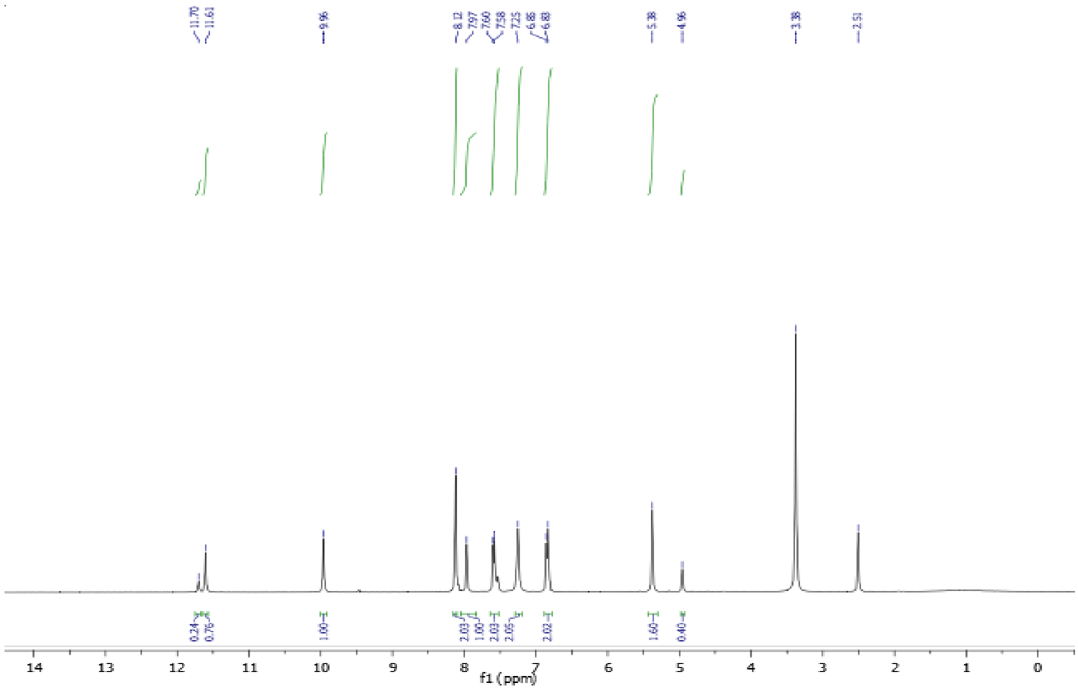

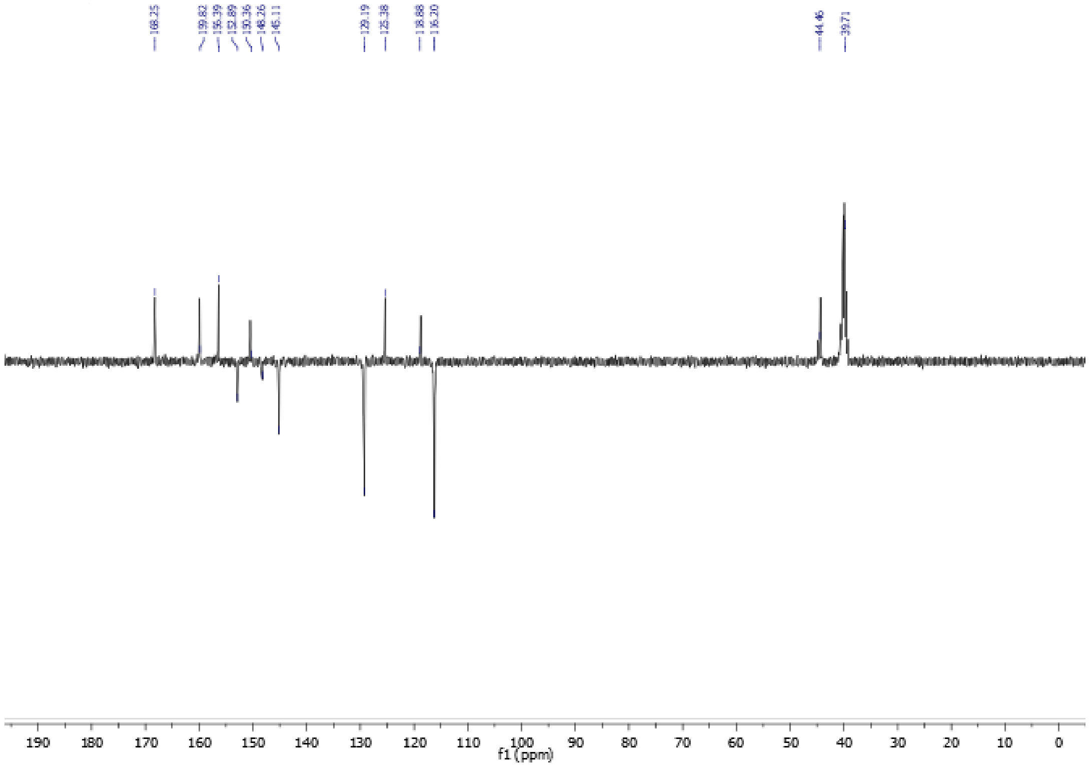
168.25
159.82
156.39
152.89
150.36
148.26
145.11
129.19
125.38
118.88
116.20
44.46
39.71
190
180
170
160
150
140
130
120
110
100
90
80
70
60
50
40
30
20
10
0
f1 (ppm)

2-(6-Amino-9*H*-purin-9-yl)-*N*'-(pyridin-4-ylmethylene)acetohydrazide (4m)

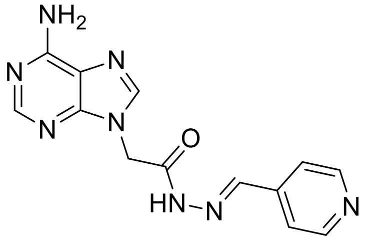

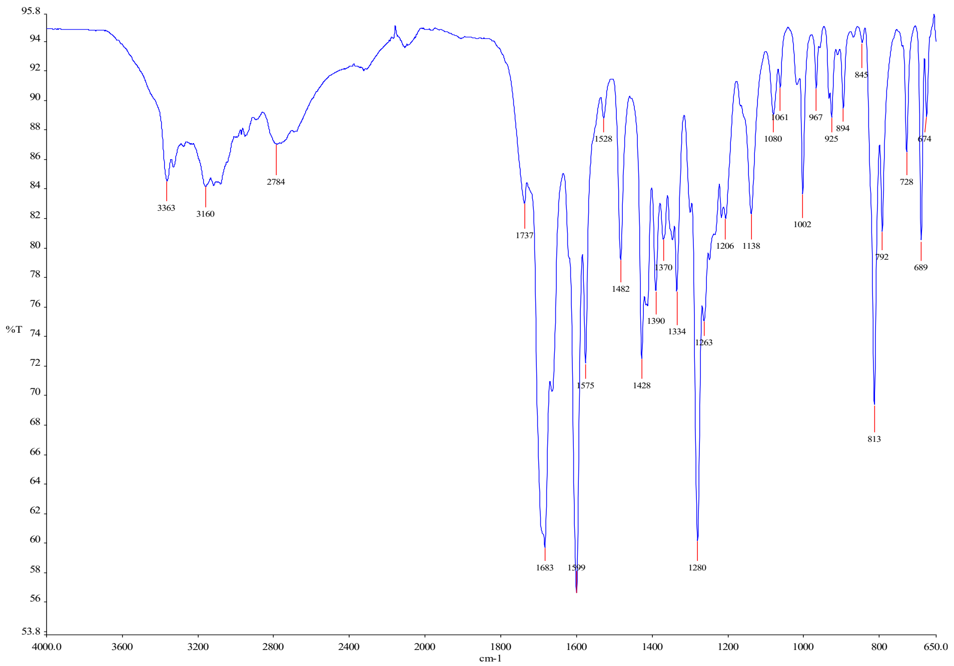

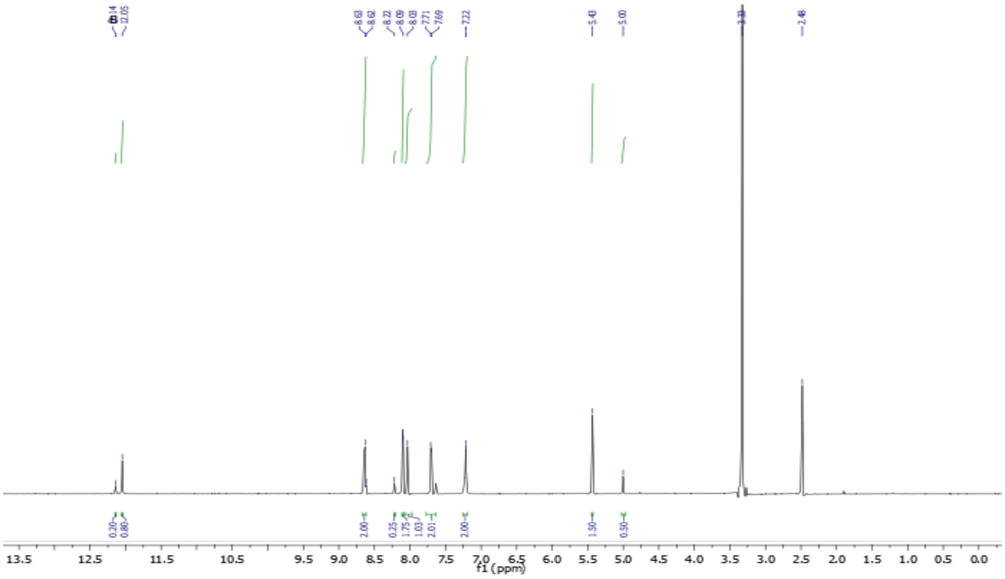

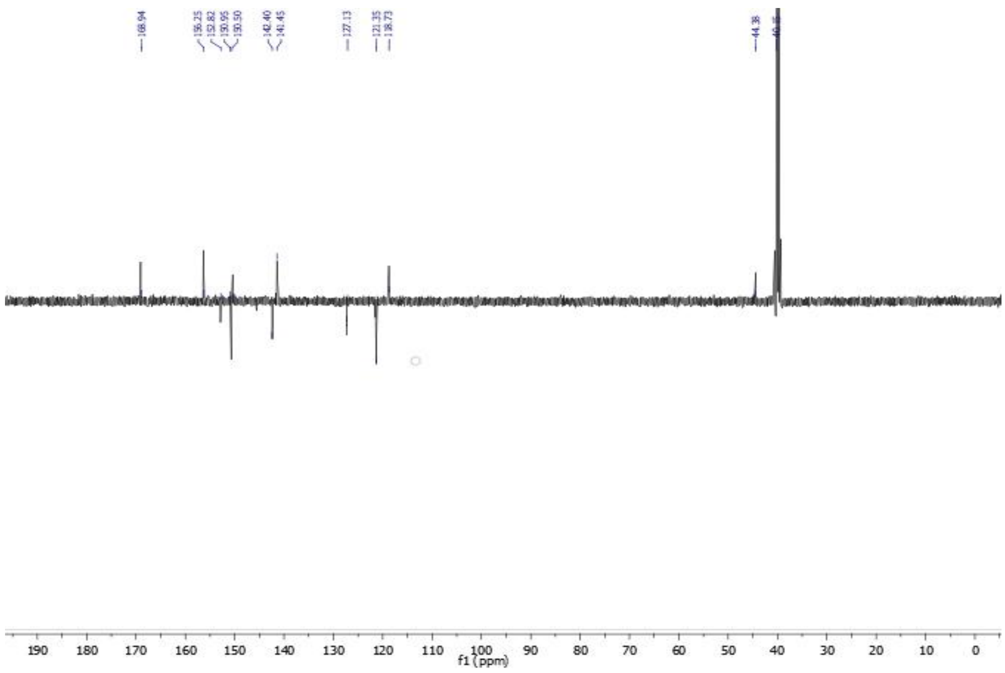
168.94
156.25
152.82
150.95
150.50
142.40
141.45
127.13
121.35
118.73
44.38
190
180
170
160
150
140
130
120
110
100
90
80
70
60
50
40
30
20
10
0
f1 (ppm)

2-(6-Amino-9*H*-purin-9-yl)-*N*'-(furan-2-ylmethylene)acetohydrazide (4n)

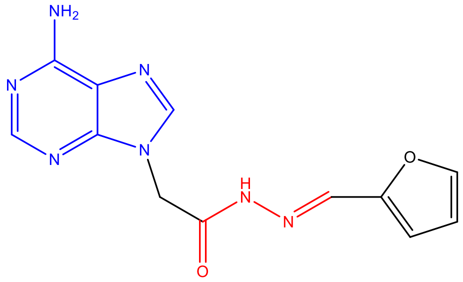

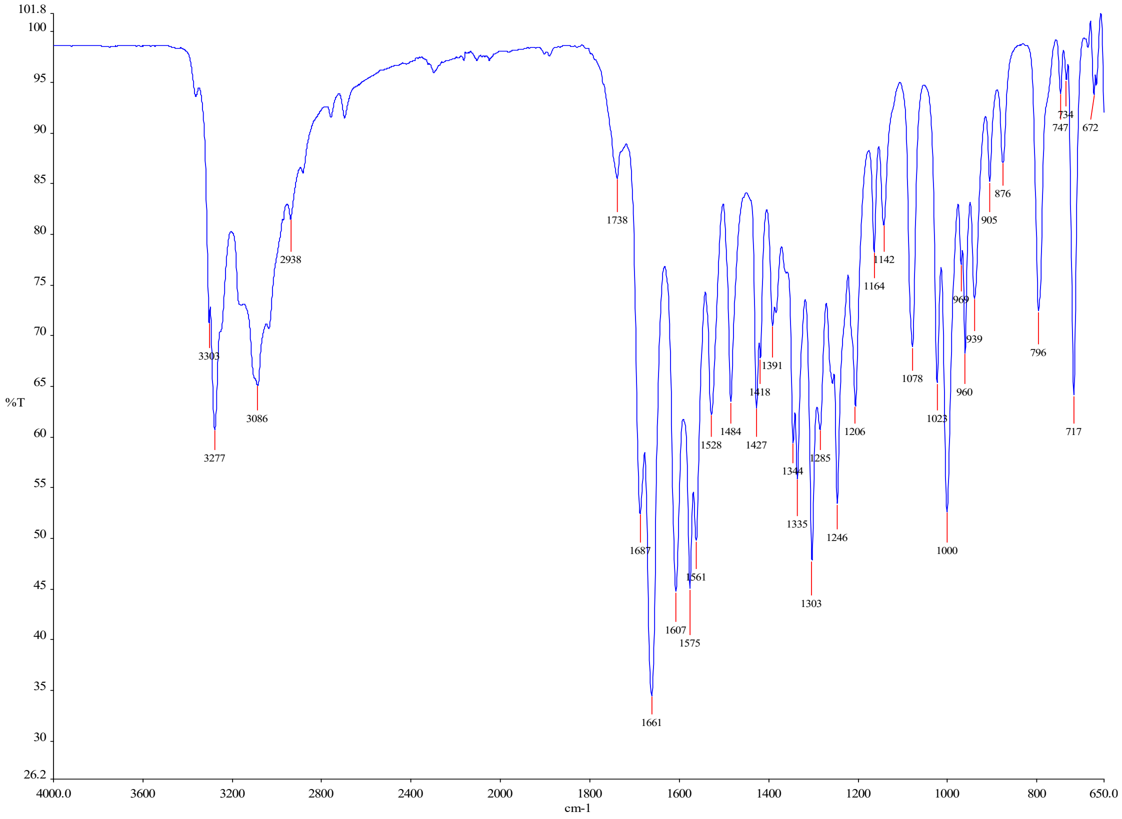

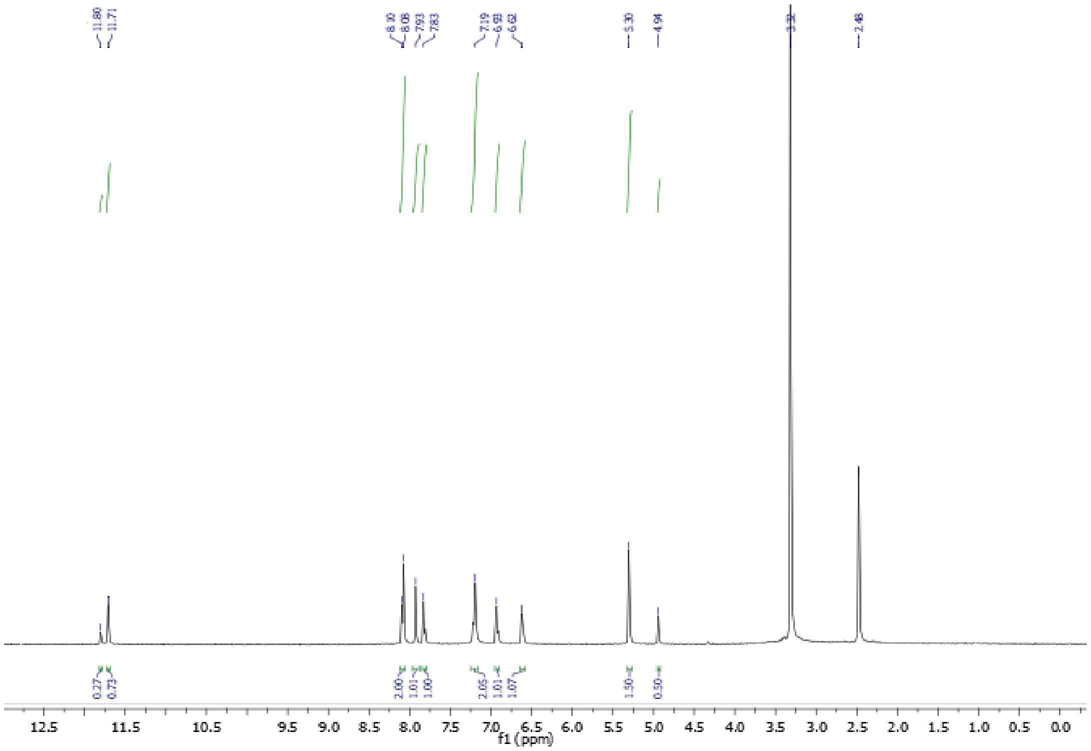
11.80
11.71
8.10
8.08
7.93
7.83
7.19
6.93
6.62
5.30
4.94
3.32
2.48
0.27
0.73
2.00
1.01
1.00
2.05
1.01
1.07
1.50
0.50
f1 (ppm)

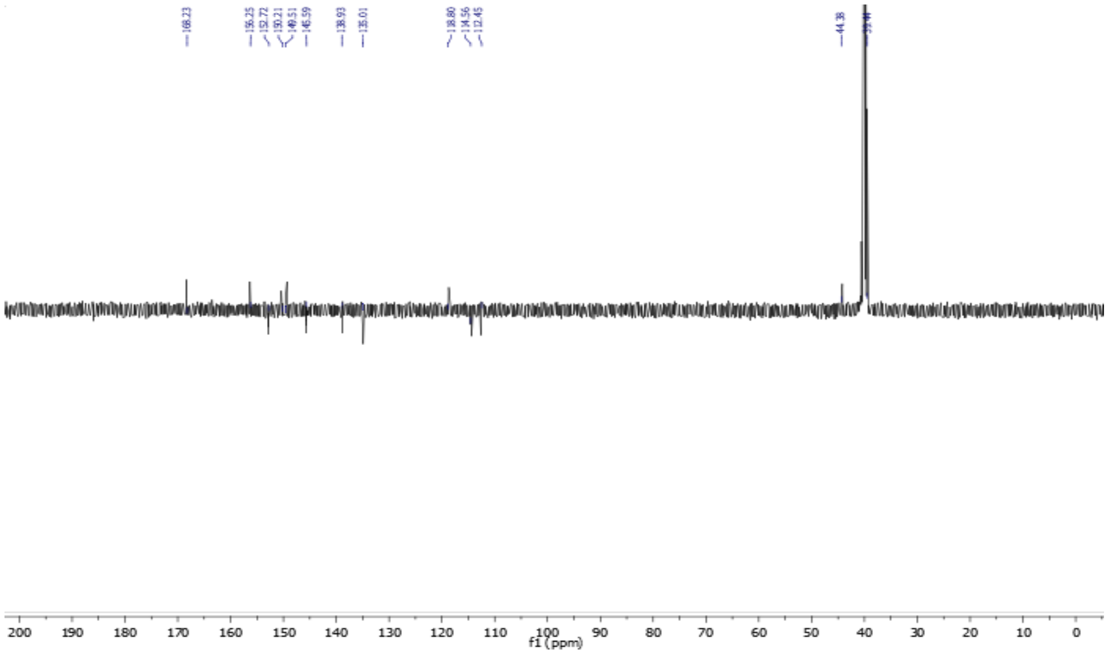
168.23
155.25
152.72
150.21
149.51
145.59
138.93
135.01
118.80
114.56
112.45
44.38
f1 (ppm)

2-(6-Amino-9H-purin-9-yl)-N'-((benzo[d][1,3]dioxol-5-yl)methylene)acetohydrazide (4o)

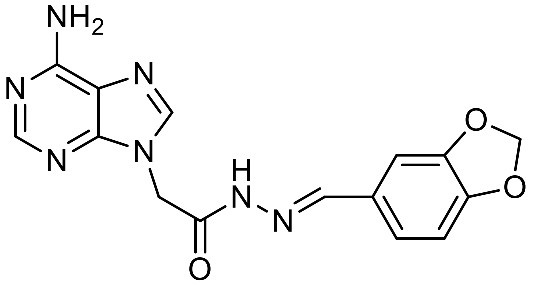

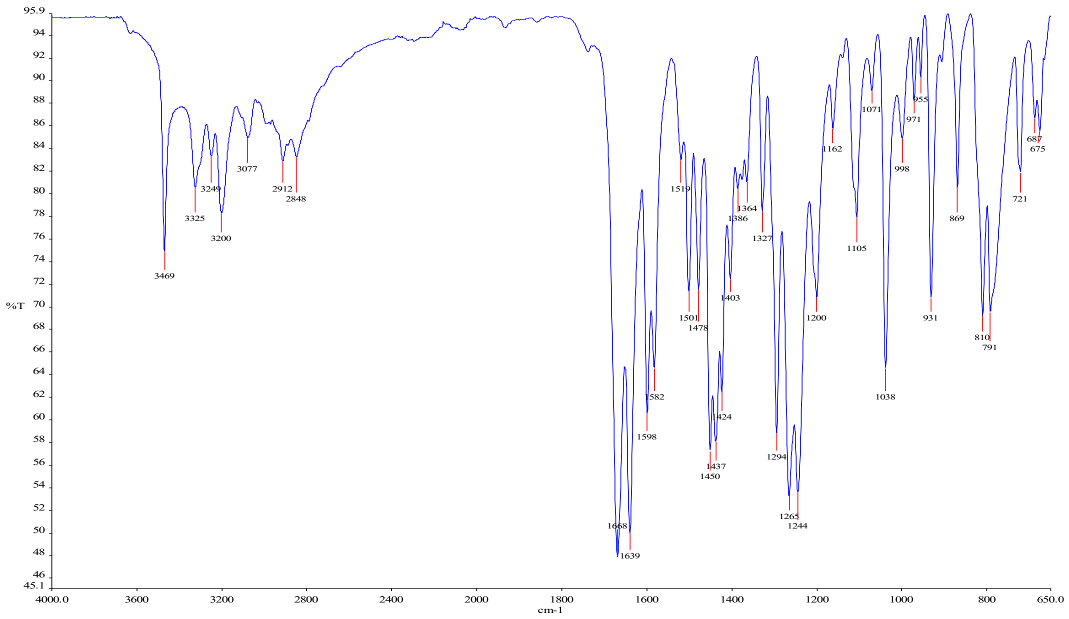

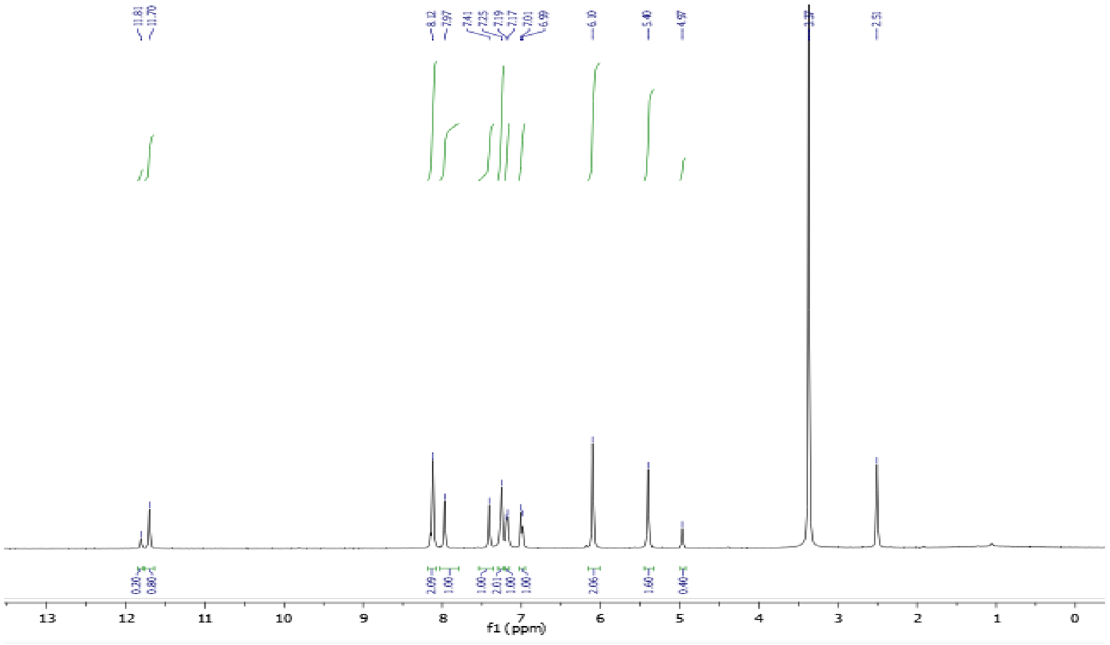

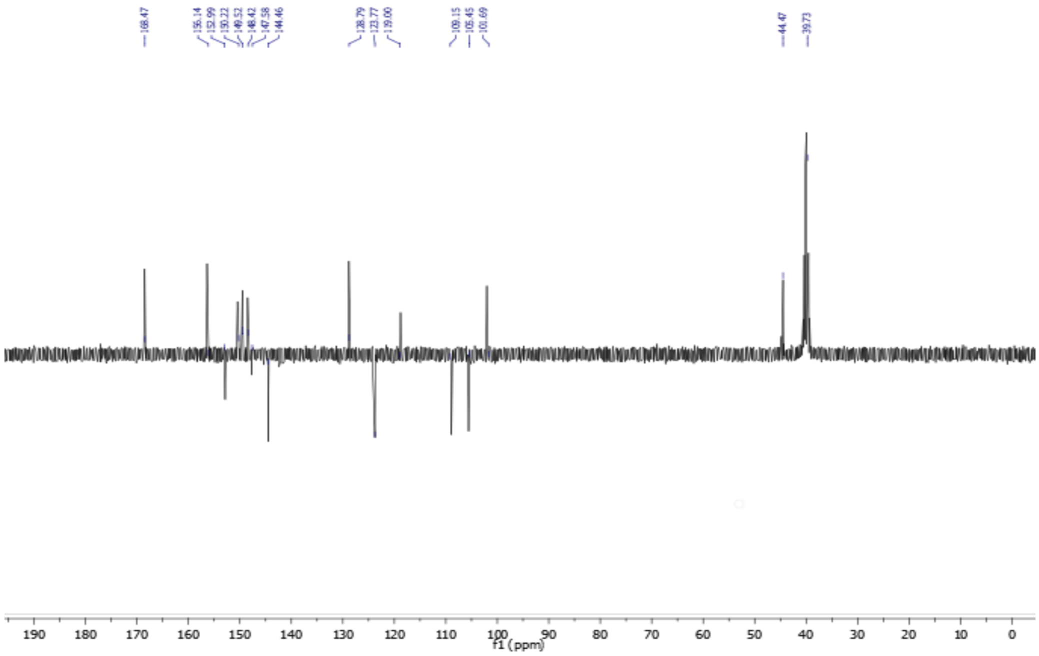
168.47
156.14
152.99
150.22
149.52
148.42
147.58
144.46
128.79
123.77
119.00
109.15
105.45
101.69
44.47
39.73
190
180
170
160
150
140
130
120
110
100
90
80
70
60
50
40
30
20
10
0
f1 (ppm)

2-(6-Amino-9*H*-purin-9-yl)-*N*'-(4-(4-phenylpiperazin-1-yl)benzylidene)acetohydrazide (4p)

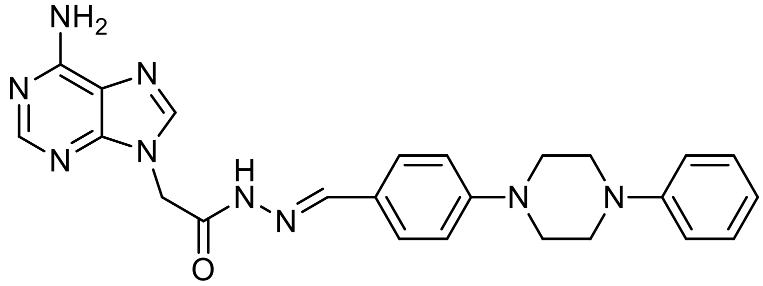

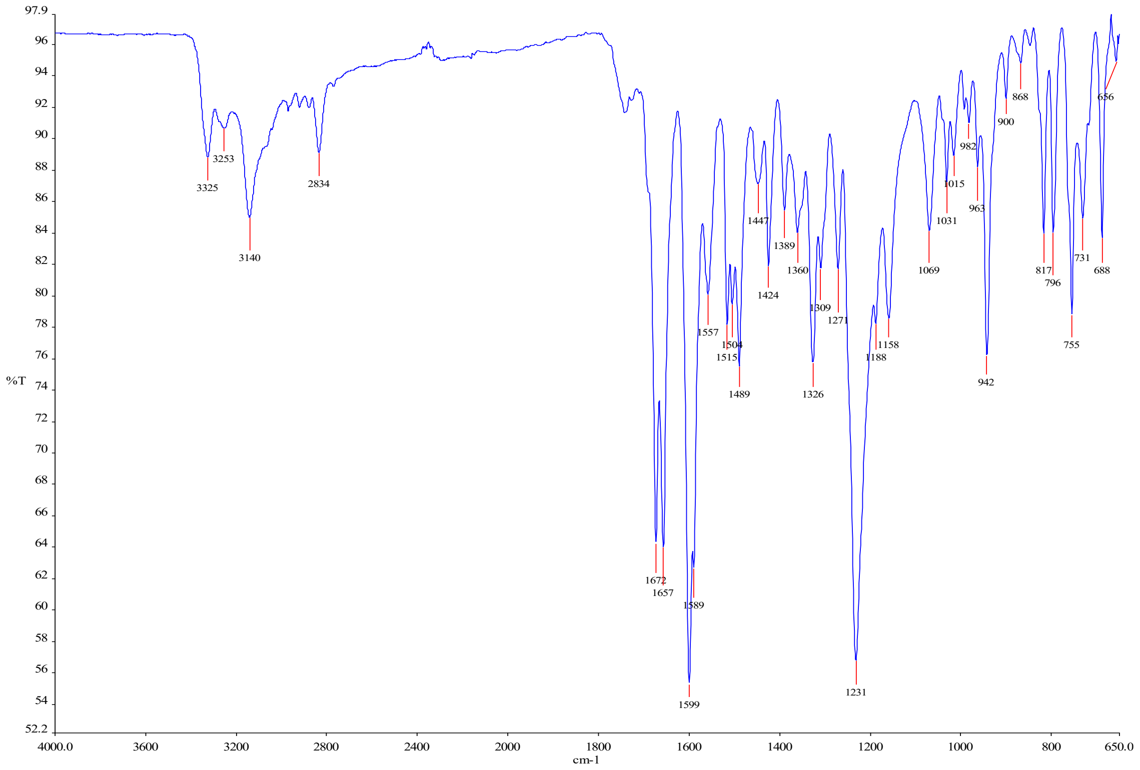

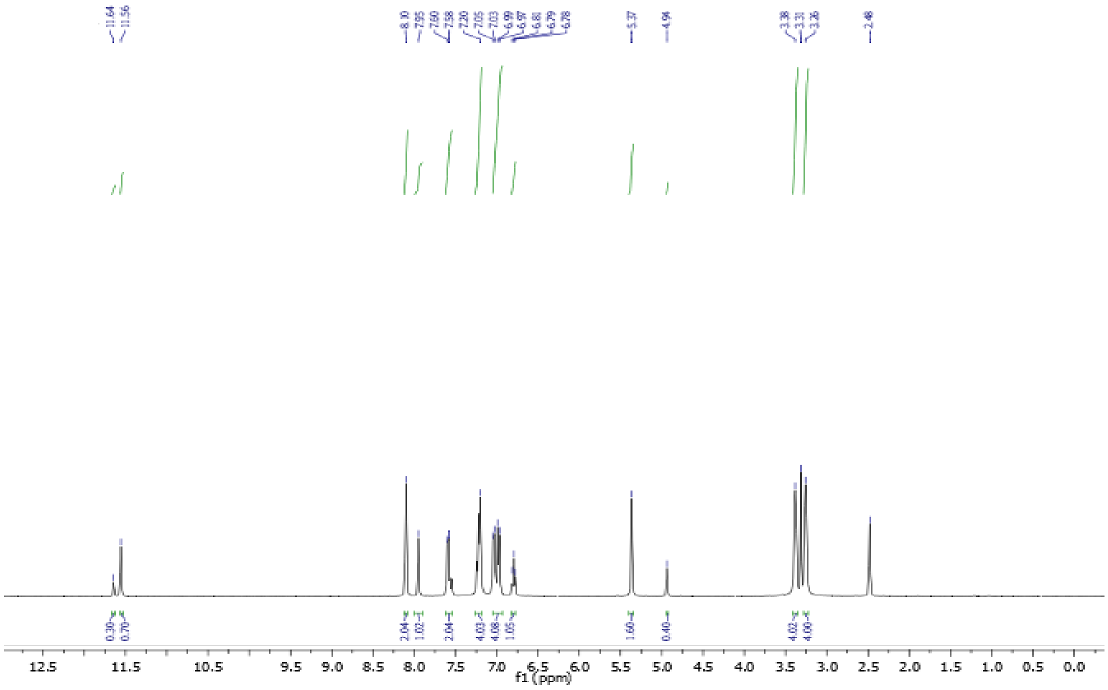
11.64
11.56
8.10
7.95
7.60
7.58
7.20
7.05
7.03
6.99
6.97
6.81
6.79
6.78
5.37
4.94
3.38
3.31
3.26
2.48
0.30
0.70
2.04
1.02
2.04
4.03
4.08
1.05
1.60
0.40
4.02
4.00
f1 (ppm)

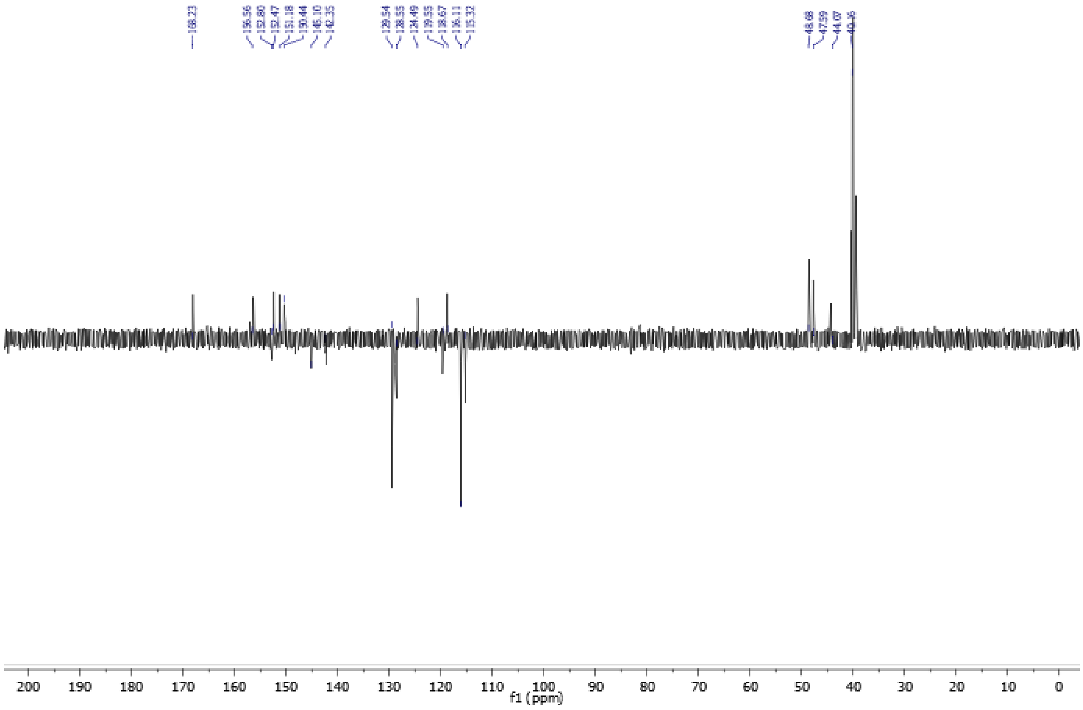
168.23
156.56
152.80
152.47
151.18
150.44
145.10
142.35
129.54
128.55
124.49
119.55
118.67
116.11
115.32
48.68
47.59
44.07
40.16
f1 (ppm)
